# Extreme weather events and Spatio-temporal characterization of climate change variables in Bangladesh during 1975–2019

**DOI:** 10.1016/j.heliyon.2024.e27118

**Published:** 2024-02-27

**Authors:** Shanjana Haider, Md Rezaul Karim, Md Saiful Islam, Tanzilla Aktar Megumi, Quazi Shahnewaz Rahnama

**Affiliations:** Department of Civil and Environmental Engineering, Islamic University of Technology (IUT), Gazipur 1704, Bangladesh

**Keywords:** Extreme indices, Trend, Sub-trend, Innovative trend analysis, Standard anomaly index

## Abstract

Bangladesh is susceptible to climate change, thus a detailed study, including the analyses of trends, sub-trends, extreme events and indices was conducted to obtain a complete picture of the climate change pattern in Bangladesh utilizing daily rainfall, maximum, minimum and average temperature data of 26 stations from 1975 to 2019 using R 4.0.2 software. For the trend analysis Mann Kendal (MK), modified Mann Kendall (mMK), Innovative Trend Analysis (ITA) and Sen’s slope methods were used. Sub-trend analysis was conducted using ITA. Standard Anomaly Index (SAI) has been used to identify the frequency and severity of extreme events. ClimPACT2 software was used to check the homogeneity and calculate the extremes of temperature and rainfall data. Our analysis showed that during the last four decades, climate variables changed their patterns and trend heterogeneously over Bangladesh. Most stations showed decremental rainfall trend when central part of the country showed a substantial decrease. The northern and central parts of the country showed significant growth of trend for annual average temperature. The temperature in the monsoon season increased, whereas those in dry season decreased. The rainfall and maximum temperature were inversely related during monsoon whereas during dry season both of them decrease. The pre-monsoon and post-monsoonal rainfall also showed decreasing trends, indicating prevailing drought conditions especially in northern and central parts of the country. The SAI analysis showed alternating drought and wet years in almost all the stations. In the past 20 years, the country’s western region experienced more drought years than before whereas the coastal region experienced more wet years. The analysis of climate extreme indices suggests that, Consecutive Dry Days (CDD), Hot Days (TX90P) and Hot Nights (TN90P) show significant increasing trend throughout the country. The agricultural productivity, water resource management and food security are anticipated to benefit from this study.

## Highlights

• Comprehensive study of the climate change pattern of entire Bangladesh.
• Climate variables changed their patterns and trends heterogeneously over Bangladesh during the last four decades.
• Decremental rainfall trend was found in majority stations.
• Alternating drought and wet year were found in almost all the stations.
• The extreme indices related to temperature showed more statistically significant results.

## 1 Introduction

Bangladesh is a small developing country in South Asia that has been greatly impacted by climate change in recent years. At present, Bangladesh experiences less rainfall during the rainy season, a warmer winter or dry season, the dominance of warm years, abnormal seasonal variation, drought conditions during pre- and post-monsoon seasons, making the country more prone to the effects of climate change. Intergovernmental Panel on Climate Change (IPCC) has also recognized Bangladesh as one of the most threatened countries in the world by the unfortunate impacts of climate change [1]. According to the IPCC 2007 report, severe rainfall events in the future will be more intense, although the yearly number of rainy days will decrease overall by up to 15 days. Additionally, an increase of 3.3 °C in the annual mean temperature is expected over a large part of South Asia by the end of the 21st century [1]. Thus, the micro-level analysis of detailed characteristics of crucial climatic variables namely, rainfall and temperature to identify the impact and pattern of climate change and the occurrence of extreme events is of great importance for the management of water resources, crop production and the life of the general public [2].

Statistical models are typically utilized to comprehend the frequency of extreme events and climate change patterns using historical data. These models are a useful tool for analyzing the trends in temperature and rainfall [2,3]. The main idea behind focusing on historical weather data is to achieve a comprehensive understanding of the past weather conditions which helps to identify long-term climate trends, regional variations, and potential climate change effects, vulnerability of a geographic area or a river basin to extreme events. Until now, different statistical nonparametric models, such as Mann-Kendall (MK) test [[4], [5], [6]], Pettitt test [7], Spearman’s rho [8] and Sen’s slope estimator [9], have been applied to assess the changes in the trends of rainfall and temperature. Many studies [[10], [11], [12], [13], [14]], have been conducted to determine the trends of rainfall and temperature data for different parts of the world.

In recent years, several studies have been conducted to analyze the trends of rainfall and temperature using parametric (i.e., ANOVA, linear regression etc.) and non-parametric methods (i.e., MK, mMK etc.) in Bangladesh. Bari et al. [15] assessed rainfall variability, seasonality index and trends using MK and sequential MK methods over northern Bangladesh from 1964 to 2013 using monthly rainfall data. Khan et al. [16] identified the trends of monthly rainfall, temperature, humidity, and wind speed data using MK and Sen’s slope methods and determined the correlation between them using Pearson correlation and Spearman’s rho test for the entire Bangladesh from 1988 to 2017. Mehzabin and Mondal [17] assessed the rainfall and temperature data of Khulna using linear regression models, coefficient of variation (CV), mean standardized anomaly (Z), and precipitation concentration index (PCI) and MK test. Rahman et al. [18] used Mk, mMK, Sen’s slope, and Spearman’s rho test to identify rainfall trends, sequential MK to identify the turning points of trend over Bangladesh and used the autoregressive integrated moving average (ARIMA) model to analyze and predict rainfall trends from 1954 to 2013. Rahman and Lateh [19] used linear regression, CV and inverse distance weighted interpolation techniques to analyze the trends, variability, and spatial patterns of temperature and rainfall of the entirety of Bangladesh from 1971 to 2010. Noorunnahar and Hossain [20] analyzed rainfall data from 1952 to 2016 using non-parametric methods, such as MK, Sen’s slope, and Sen’s T test to detect the trends of seven divisions of Bangladesh. Bhuyan et al. [21] analyzed rainfall and temperature data from the Bangladesh Meteorological Department (BMD) and Coupled Model Inter-comparison Project phase 5 (CMIP5) of the north-western region of Bangladesh from 1981 to 2008 using MK, Sen’s slope, Pearson’s r, Spearman’s ρ and Kendall’s τ correlation methods.

A novel trend method to identify any hidden trends in a time-series dataset was proposed by Şen [22] and has garnered significant attention in the numbers of global regions [[23], [24], [25], [26]]. This method enables more detailed interpretations of trend identification, which is advantageous for identifying hidden sub-trends and the graphical representation of trend variability of extreme events, such as "high" and "low" values of climatic variables [27]. Previously, Das et al. [2] and Islam et al. [28] applied the Innovative Trend Analysis (ITA) method but no study has used the ITA method for both rainfall and temperature data to analyze both trends and sub-trends of these variables, which is essential to understand the detailed patterns and inter-relationship of these two climatic variables.

SAI is another important statistical analysis method to determine the drought and wet years, and cold and warm years. It can be used to measure the frequency and severity of extreme events. Additionally, several indices have been defined by the World Meteorological Organization (WMO) expert panel on climate change to create a consistent framework for climate change analysis at various spatial and temporal scales [29]. Studies [[30], [31], [32], [33], [34]] have been undertaken at different areas to understand the changes in climatic indices for the adaptation plan. These indicators are used as analytical instruments for describing the climatic system of a region [32].

To the best of our knowledge, none of the previous studies analyzed the trends, sub-trends, extreme events and indices of both temperature and rainfall to get a comprehensive picture of the climate change pattern of the entirety of Bangladesh. To fill this gap in knowledge, an extensive statistical analysis was performed using meteorological data to explore the coherent trends and sub-trends of related climatic variables, explain their changes in the time series, and identify the pattern of occurrence of extreme climatic events and indices. Additionally, the results of different statistical models were compared to obtain a better understanding of their applicability and relative accuracy. As the extreme climatic indices proposed by the expert panel of WMO will be assessed in our study, our results will contribute to understanding weather and climate extremes worldwide.

Our study will contribute to answer the following research questions:

i. What is the interrelationship between the rainfall and temperature over Bangladesh or how they change with every season?
ii. Is there any hidden trend or sub-trend of rainfall and temperature?
iii. Is there any alarming trend of extreme events and indices related to rainfall and temperature?

## 2 Study area

This study was conducted for the entire Bangladesh to understand the climate change scenarios based on the observed data. Bangladesh is a country with tropical monsoon climate in Southeast Asia. Bangladesh is located between 20° 34′ N and 26° 38′ N latitude and 88° 01′ E and 92° 41′ E longitude, with an area of 144,000 km^2^. Compared with that of other tropical regions, the climate of Bangladesh has unusual characteristics of heavy rainfall, high temperatures, and seasonal variation constitutes the unusual characteristics of the climate of Bangladesh from other tropical regions. It has the largest delta in the world, with the Brahmaputra, Ganges, and Meghna river systems flowing toward the Bay of Bengal, and a huge area of Bangladesh namely, the Sylhet and Chattogram divisions, experiences frequent flooding, especially flash floods. Conversely, the Barind track on the northern and northeastern sides of Bangladesh experiences frequent drought [35].

**Fig. 1:**
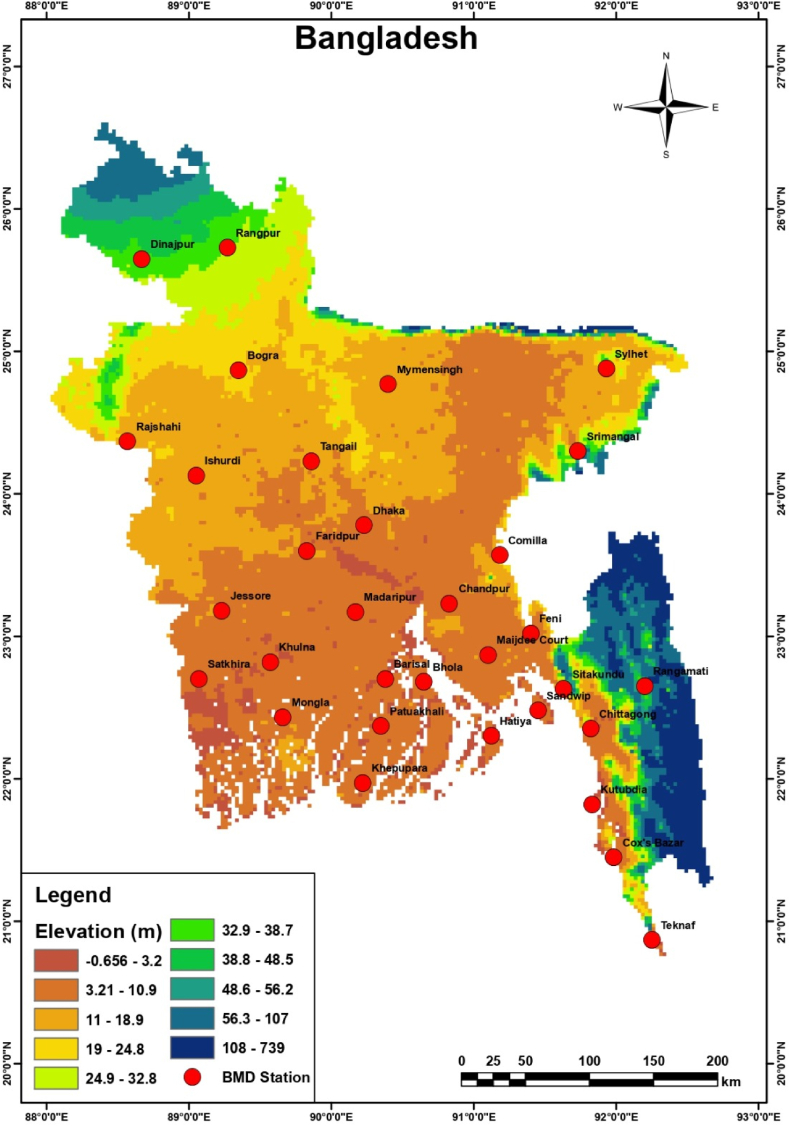
Location and topographical map of Bangladesh showing meteorological stations.

Bangladesh is characterized by four distinct climatic seasons, namely, Pre-monsoon (March-April-May), Monsoon (June-July-August-September), Post-monsoon (October–November), and Winter or Dry (December-January-February) seasons. The annual temperature varies between 9.4 °C and 36.6 °C. The temperature varies between 15.9 °C and 36.6 °C, 24.7 °C and 34.6 °C, 17.8 °C and 32.6 °C, and 9.4 °C and 29.0 °C during the pre-monsoon, monsoon, post-monsoon and winter seasons, respectively. The amounts of rainfall during the pre-monsoon, monsoon, post-monsoon, and winter seasons are 369, 1506, 267, and 5 mm, respectively, according to BMD as of 2020. The topography and meteorological stations of the study area is shown in Fig. 1.

## 3 Data and methodology

For climate change analysis, daily rainfall, maximum, minimum and average temperature data has been collected from Bangladesh Meteorological Department (BMD). There are 34 BMD stations all over Bangladesh but some of them were recently introduced and data of some stations are not consistent. As a result, we excluded the stations who had >10% missing data and considered meteorological data of 26 stations which have <10% missing data from 1975 to 2019 which covers entire Bangladesh. Location and topographical map of Bangladesh showing meteorological stations is shown in Fig. 1. Arc GIS 10.3 and R 4.0.2 software have been used for mapping and data analyzing. The missing data in the time series has been calculated using the Random Forest method. After that the time series has been pre-whitened to eliminate serial correlation. There are two types of trend test i.e., parametric and non-parametric trend tests. Parametric tests need to satisfy both distributional and independent or uncorrelated assumptions, whereas nonparametric tests need to satisfy the second assumption only. Thus, we applied non-parametric trend tests as they can deal with non-normal and missing data. ITA has been used to identify trends along with sub-trend analysis to evaluate the climate pattern of annual and seasonal rainfall and temperature data. The reliability of ITA method has been checked with the traditional approach i.e., MK and mMK, Sen’s slope estimator to find the magnitude of trend and compare with the results of ITA. Standard Anomaly Index (SAI) has been used to identify frequency, severity and occurrence of extreme natural events i.e., drought and flooding in Bangladesh. Lastly, ClimPACT2 software has been used to check homogeneity and calculate extremes of temperature and rainfall using daily observed data. The research flow diagram has been shown in Fig. S1. The detailed descriptions of the various techniques used in this work are described in the sections below.

### 3.1 Missing data calculation

Daily rainfall as well as daily maximum, minimum, and average temperature records, were collected from the Meteorological Department of Bangladesh. However, some data were missing (<5%) which could have led to inconsistent results from the analysis. Owing to that, the Random Forest method was utilized to eliminate the discrepancy. RStudio’s MICE algorithm package was used for approaching the random forest method.

### 3.2 Serial correlation

In time series data, serial dependence or existence of autocorrelation creates confounding effect. It should be removed in order to obtain the Mann-Kendall test's true results because it may indicate an unusual trend. The non-parametric test will specifically imply a significant trend in a time series that is actually random more frequently than allowed by the significance level if there is a positive serial correlation in the time series [36]. In this regard, Von Storch [37] recommended that the time series be "pre-whitened" to remove the impact of serial correlation before conducting the Mann-Kendall test.

### 3.3 Mann Kendall (MK) test

The MK Trend Test is a non-parametric test proposed by Mann [4] and Kendall [5,6] and can be used with any distribution but it must be free of serial correlation. The null hypothesis (H_0_) for the test is that the series does not have a monotonic trend. Alternative hypothesis (H_A_) is that there is a trend which can be neutral, positive, or negative. The idea of this assumption is that if a pattern exists, the symbol values should tend to increase or decrease on a regular basis. This is the most common method to identify trend in hydro-meteorological time series. Package “trend” of R Studio was used to conduct the analysis.

The Z is the true trend indicator of the test statistics and if falls beyond level ±1.96 then indicates significant trend at 5% significance level. Trends that are increasing or decreasing are represented by positive and negative Z values, accordingly. There is no trend in the time series, according to the null hypothesis (H_0_) or 0 value.

### 3.4 Modified Mann Kendall (mMK) test

Modified Mann Kendall method is originally the updated form of traditional MK method. Due to the auto correlation function, the traditional MK method sometimes may have some false positive error. Thus, modified MK approach which was proposed by Hamed and Rao [38] have been used to eliminate the error using the modified VAR(S) statistic. For this analysis “trend” package of R studio was used.

### 3.5 Sen's slope estimator

Sen's slope estimator proposed by Sen [9] was used to calculate the magnitudes of the rainfall and temperature trend (slope Q). Positive and negative value of slope estimator indicates ascending and descending rate of magnitude respectively. Package “trend” of R studio was used for this analysis.

### 3.6 Innovative trend analysis (ITA) method

Şen [22] was the first to suggest the visual non-parametric ITA approach. This method follows dividing the time series in two equal parts and plotting them against each other on the Cartesian coordinate system where the first half of data is plotted on the x-axis and the other on the y-axis. If all the data point falls along the 1:1 (45°) line then there is no trend. But if they fall above the 1:1 (45°) line and in the upper triangular area then there is positive trend and vice versa [22]. It can also deal with autocorrelation and outliers. As we took data of 45 years (1975–2019) so we omitted the data of the year 1975 and then divided it into two halves. Package “trendchange” was used for ITA analysis.

The high, medium, and low values of the given data can be graphically evaluated in this approach and they can be classified in “low”, ‘’medium” and “high” value groups as per Fig. S2. The data is classified based on the data range of a station as all the stations don’t receive same amount of rainfall due to geographic and land use condition. Two 5% and 10% confidence bands have been plotted along the 1:1 line to understand the significance of the sub-trend and difference between the trend line and the data points which was also followed by Alifujiang et al. [39]. Sub-trend analysis helps to identify the underlying trend (non-monotonic trend) in a time series data by removing the seasonal and cyclical components. It is useful in identifying the long-term trend in a variable, which can be obscured by short-term fluctuations. It is essential for forecasting and decision-making as trend analysis only shows results considering the majority data.

### 3.7 Standardized Anomaly Index (SAI)

Standardized Anomaly Index (SAI) is a frequently used index introduced by Kraus [40] to identify the fluctuations of rainfall and temperature over the years which is a measure of deviation in standard units, between a data value and its mean. For temperature negative values represents cold years and positive values represent warm years. Similarly, positive values refer to wet years and negative value refer to dry years in case of using rainfall data. It measures the frequency and severity of droughts. SAI value classification is provided in Table S1. Package “precintcon” was used to conduct SAI analysis in R studio.

### 3.8 Data homogeneity

The homogeneity test has been undertaken and adjustment for possible multiple change points, specially shifts in the mean that may have first order autoregressive errors have been conducted using RHtestsV4 software package which was proposed by Wang and Feng [41]. Daily rainfall and temperature data were used to undertake the homogeneity test. This test is important to check the consistency of any data series before under taking any statistical analysis. It is based on the penalized maximal *t*-test proposed by Wang et al. [42] and the penalized maximal F test proposed by Wang [43].

### 3.9 Extreme climate indices

A core set of 27 extreme indices for temperature and precipitation was developed by the Expert Team on Climate Change Detection and Indices (ETCCDI). The indices describe particular characteristics of extremes, including frequency, amplitude and persistence [29]. Zhang et al. [44] stated that, it is possible to assimilate the results of studies of different parts of the world to attain a coherent picture of global extreme changes by using such extreme metrics. The extremes of temperature and precipitation have been calculated using ClimPACT2 software using 45 years (1975–2019) of daily recorded data. We accounted 21 extreme indices as they are relevant to the climatic condition of the study area in identifying extremes related to maximum and minimum temperatures and precipitation. Considered Extreme temperature and precipitation indicators are provided in Table S2.

## 4 Result

During initial analysis of data mean, standard deviation, variance and co-efficient of variation (CV) was analyzed. The results indicate that mean annual rainfall, maximum and minimum temperature data varied between 1435.44 and 4168.6 mm, 31.43 °C–35 °C, and 16 °C–19.4 °C, respectively. CV of the annual rainfall dataset indicates low (13.63%) to medium (25.64%) variability of distribution. CV of maximum and minimum temperature varies between 1.09% to 2.64% and 2.56%–4.29%, respectively. Stations with low yearly rainfall, minimum and maximum temperature variability are primarily located in the country's southern, southeast, and northeast parts, while the stations with higher rainfall, minimum and maximum temperature variability are discretely scattered throughout the study area. Mean, Standard Deviation, Variance and CV of average rainfall, maximum and minimum temperature of all the stations are provided in Table S3, S4 and S5, respectively.

### 4.1 Trend analysis

#### 4.1.1 Trend analysis of annual rainfall

From ITA (Table 1 and Fig. S3), negative and positive trends were observed in 50% and 35% of the stations at a significance level (SL) of 5%. The increasing trend was mainly observed in the south-western, southern, south-eastern and eastern regions of the country. The decreasing trend was observed in the western, northern and central parts of the country. The highest increasing and decreasing trends were observed in Sandwip and Faridpur, respectively. Faridpur showed the maximum rate of rainfall decrease of 11.84 mm/yr, and Dhaka showed a rate of rainfall decrease 9.31 mm/yr. The maximum growth rate of rainfall was 13.42 mm/yr at Sandwip and the posterior growth rate of rainfall was 10.9 mm/yr at Hatiya.

**Table 1:**
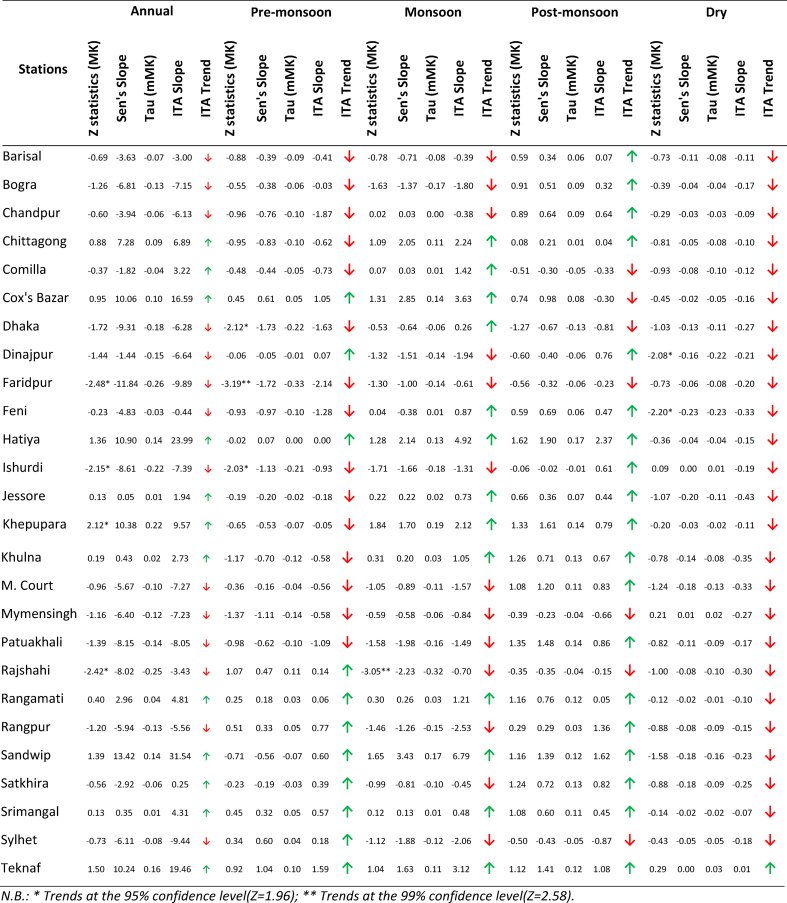
Details of annual and seasonal rainfall trend in Bangladesh.

From MK and mMK tests, approximately 27% of the stations showed increasing trends, among which only Khepupara showed significant increasing trends at SL of 5%, with a rate of increase of 10.375 mm/yr. The remaining 73% of stations showed decreasing trends, among which only Faridpur (11.84 mm/yr), Ishurdi (8.61 mm/yr) and Rajshahi (8.02 mm/yr) showed significant decreasing trends at 95% confidence level (CL).

#### 4.1.2 Trend analysis of seasonal rainfall

##### 4.1.2.1 Pre-monsoon

The ITA results reveal that 46% of the stations located in western, south western and central parts of the country showed decremental trends, where only Faridpur shows a statistically significant decrease at 95% CL. Cox’s Bazar, Rangpur, Sandwip, Srimangal, and Teknaf which are located at the very north western, northeastern and very south eastern sides of the country, showed nonsignificant increasing trends. The MK and mMK results showed similar decreasing trends in more districts (58%), among which Dhaka, Faridpur, and Ishurdi showed statistically significant decreasing trends at 95% CL. Cox’s Bazar, Rajshahi, Rangpur, and Teknaf showed increasing trends but none of them were significant. The highest magnitude of increase was 1.04 mm/yr in Teknaf and the lowest magnitudes of increase were 1.73 and 1.72 mm/yr at Dhaka and Faridpur respectively. The MK, mMK, ITA and Sen’s slope of rainfall during pre-monsoon have been shown in Table 1 and Fig. S3.

##### 4.1.2.2 Monsoon

The ITA slope for rainfall during the monsoon season showed (Table 1 and Fig. S3) increasing trends in 46% of the stations located in the southern belt near the Bay of Bengal. The decreasing trend dominates in 38% of the stations located in northern and central parts of Bangladesh. Only Sylhet showed significant decreasing trends in rainfall during the monsoon season. The MK and mMK tests showed similar results. The maximum magnitude of increase was 3.43 mm/yr in Sandwip and the maximum magnitude of decrease was 2.23 mm/yr in Rajshahi.

##### 4.1.2.3 Post-monsoon

The ITA results of the post-monsoon season showed (Table 1 and Fig. S3) increasing trends in 62% of the stations, wherein only Hatiya showed significant increase at 95% CL. Other stations, present mostly in the north eastern, south eastern, and central parts, showed insignificant decreasing trends. MK and mMK tests showed approximately similar results for both the increasing and decreasing trends. The maximum magnitude of increase was 1.90 mm/yr in Hatiya and maximum magnitude of decrease was 0.67 mm/yr in Dhaka.

##### 4.1.2.4 Winter or dry season

The ITA results indicated (Table 1 and Fig. S3) that all the stations, except Teknaf showed decreasing trends in rainfall. The increasing trend in Teknaf is not significant. According to the MK and mMK tests, Dinajpur and Feni showed significant decreasing trends in rainfall at 95% CL. The maximum magnitude of decrease was 0.23 mm/yr in Feni from Sen’s slope analysis.

#### 4.1.3 Trend analysis of annual average temperature

**Fig. 2:**
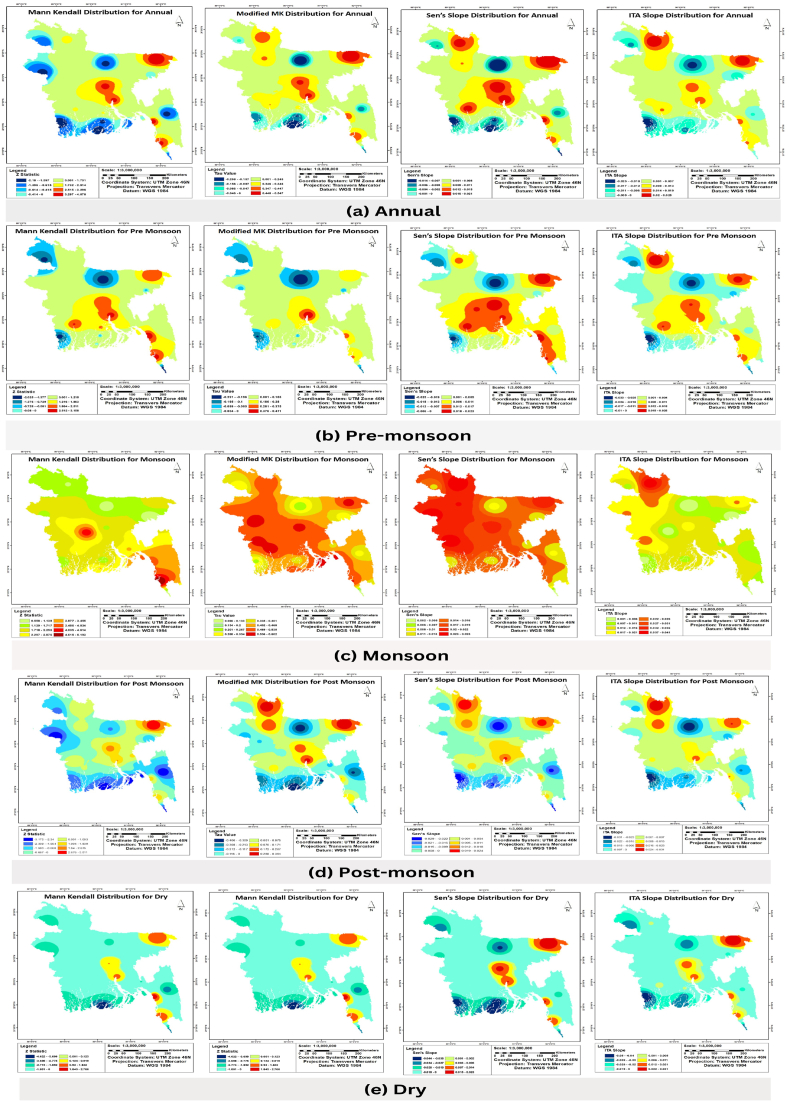
Spatial distribution of (a) Annual, (b) Pre-monsoon, (c) Monsoon, (d) Post-monsoon and (e) Dry average temperature trend using MK (1st column), mMK (2nd column), ITA (3rd column) and Sen’s slope (4th column) analysis.

The ITA and mMK test results showed (Table 2 and Fig. 2) increasing trends in temperature 54% of the stations, and only certain small regions in north central, north western, and south western sides showed decreasing trends. MK analysis showed slightly different results, namely, significant increasing trends in 23% of the stations located in the central, south eastern and south western parts of the country. The highest increasing and decreasing trends were observed in Chittagong and Teknaf, respectively. According to Sen’s slope analysis, Chittagong and Dhaka showed maximum rates of temperature increase of 0.023 °C/yr and Mymensingh showed a maximum rate of temperature decrease of 0.026 °C/yr.

**Table 2:**
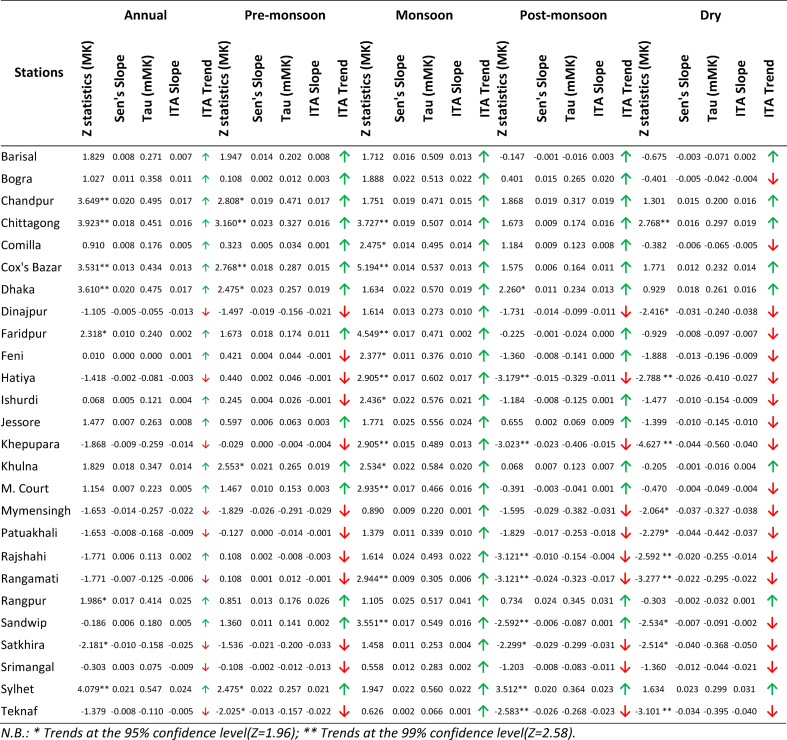
Details of annual and seasonal average temperature trend in Bangladesh.

#### 4.1.4 Trend analysis of seasonal average temperature

##### 4.1.4.1 Pre-monsoon

The ITA and mMk analysis results showed (Table 2 and Fig. 2) increasing trends in 54% of the stations located in the north central, north western and south western side. However, MK analysis showed slightly different results. MK analysis showed significant increasing trends in 23% of the stations in the central, south eastern, and south western parts of the country and significant decreasing trends in Teknaf. According to Sen’s slope analysis, Chittagong and Dhaka showed maximum rates of temperature increase of 0.023 °C/yr and Mymensingh showed maximum rate of decrease of 0.026 °C/yr.

##### 4.1.4.2 Monsoon

The ITA and mMK analysis results showed (Table 2 and Fig. 2) increasing trends at all the stations during the monsoon season. MK analysis showed significant increasing trends at 95% CL in Chittagong, Comilla, Cox’s Bazar, Faridpur, Feni, Hatiya, Ishurdi, Khepupara, Khulna, M. Court, Rangamati, and Sandwip which are located in the central and southern part of the country; Cox’s Bazar showed the highest increasing trend. From Sen’s slope analysis, Jessore and Rangpur showed maximum rates of temperature increase of 0.025 °C/yr.

##### 4.1.4.3 Post-monsoon

The ITA analysis results showed (Table 2 and Fig. 2) increasing trends in 54% of the stations, decreasing trends in 38% of the stations, and no trends in two stations i.e., Faridpur and Feni. But However, MK analysis showed decreasing trends in 62% stations, mainly in the southern part of the country, and Dhaka and Sylhet showed significant increasing trends at 95% CL. Sylhet showed a maximum rate of temperature increase of 0.020 °C/yr and Mymensingh and Satkhira showed maximum rates of temperature decrease of 0.029 °C/yr.

##### 4.1.4.4 Dry

The ITA, MK and mMK results showed (Table 2 and Fig. 2) decreasing trends in 69% of the stations and increasing trends in Chandpur, Chittagong, Cox’s Bazar, Dhaka, Khulna, Rangpur, and Sylhet. Dinajpur, Hatiya, Khepupara, Mymensingh, Patuakhali, Rajshahi, Rangamati, Sandwip, Satkhira, and Teknaf showed statistically significant decreasing trends and Chittagong showed significant increasing trends at 95% CL. According to Sen’s slope analysis, Sylhet showed a maximum rate of increase of 0.023 °C/yr and Khepupara and Patuakhali showed maximum rates of decrease of 0.044 °C/yr.

#### 4.1.5 Trend analysis of annual maximum temperature

Except for Satkhira all the other stations showed increasing trends in annual maximum temperature, from ITA,MK and mMK resultsanalysis. Most stations showed significant increasing trends and only Satkhira showed a decreasing trend, which is not statistically significant at 95% CL. According to Sen’s slope analysis, Satkhira showed a maximum rate of decrease of 0.007 °C/yr and Sandwip showed a maximum rate of increase of 0.052 °C/yr (Table 3 and Fig. S4).

**Table 3:**
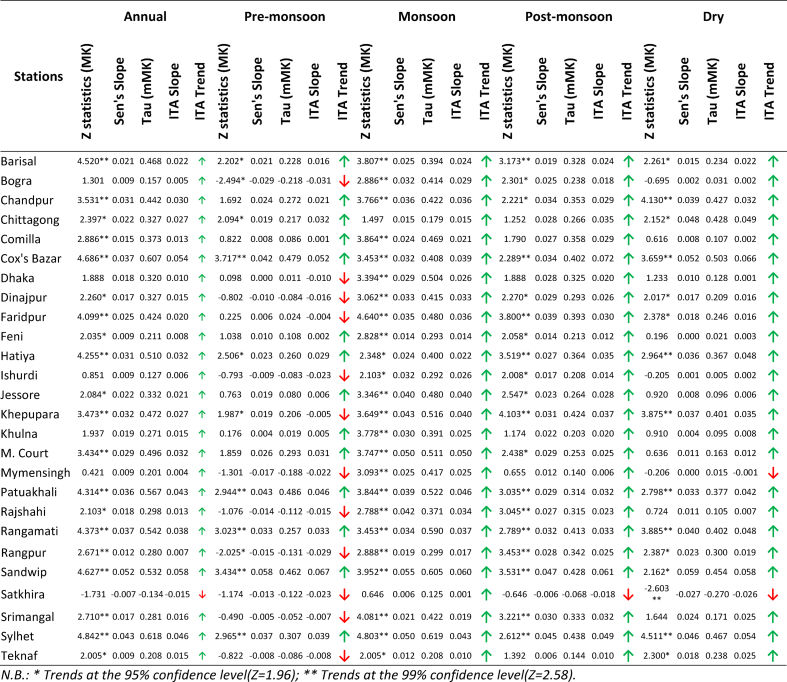
Details of annual and seasonal maximum temperature trend in Bangladesh.

#### 4.1.6 Trend analysis of seasonal maximum temperature

##### 4.1.6.1 Pre-monsoon

According to MK analysis, the south central and eastern parts of the country showed significant increasing trends and Bogra and Rangpur showed significant decreasing trends at 95% CL. According toSen’s slope analysis, Bogra showed a maximum rate of decrease of 0.03 °C/yr and Sandwip showed a maximum rate of increase of 0.058 °C/yr (Table 3 and Fig. S4).

##### 4.1.6.2 Monsoon

All the stations showed significant increasing trends except for Chittagong and Satkhira. The highest and lowest rates of increase were observed in Sandwip (0.055 °C/yr) and Satkhira (0.006 °C/yr), respectively according to Sen’s slope analysis (Table 3 and Fig. S4).

##### 4.1.6.3 Post-monsoon

The ITA, MK and mMK results showed increasing trends in all stations except Satkhira. The results of MK analysis indicated that only Chittagong, Comilla, Dhaka, Khulna, Mymensingh, and Teknaf had insignificant increasing trend at 95% CL. Satkhira showed an insignificant decreasing trend with a rate of decrease of 0.006 °C/yr. The highest rate of increase was observed in Sandwip (0.048 °C/yr) (Table 3 and Fig. S4).

##### 4.1.6.4 Winter or dry season

The ITA, MK and mMK results indicated that (Table 3 and Fig. S4) Bogra, Ishurdi, Mymensingh, and Satkhira showed decreasing trends wherein only Satkhira showed a significant decreasing trend. According to MK analysis, Barisal, Chandpur, Chittagong, Cox’s Bazar, Dinajpur, Faridpur, Hatiya, Khepupara, Patuakhali, Rangamati, Rangpur, Sandwip Sylhet, and Teknaf showed significant increasing trends. Satkhira showed a rate of decrease of 0.027 °C/yr and the highest rate of increase was observed in Sandwip (0.059 °C/yr).

#### 4.1.7 Trend analysis of annual minimum temperature

All the stations, except for Hatiya, Rangamati, and Sandwip showed increasing trends in annual minimum temperature according to the ITA, MK and mMK results (Table S6 and Fig. S5). Rangamati showed a significant decreasing trend (0.029 °C/yr). The highest rate of increase was observed in Chittagong (0.043 °C/yr).

#### 4.1.8 Trend analysis of seasonal minimum temperature

##### 4.1.8.1 Pre-monsoon

According to ITA, except for Khepupara, Patuakhali, Rangamati, and Sandwip all the other stations showed increasing trends (Table S6 and Fig. S5). The results of mMK and Sen’s slope analyses indicated that only Rangamati showed a decreasing trend at a rate of 0.025 °C/yr and Chittagong showed the highest rate of increase of 0.053 °C/yr.

##### 4.1.8.2 Monsoon

The ITA, MK, mMK and Sen’s slope results showed increasing trends at all the stations. Apart from Dhaka, Hatiya, Rangamati, Sandwip and Teknaf all the other stations showed significant increasing trends. The highest rate of increase is observed in Chittagong 0.049 °C/yr (Table S6 and Fig. S5).

##### 4.1.8.3 Post-monsoon

Except for Hatiya, Khepupara, Mymensingh, Patuakhali, Rangamati, Sandwip, and Srimangal all the other stations showed increasing trends according to the ITA, mMk and MK results. Significant increasing trends were observed in Chandpur, Chittagong, Dhaka, Rangpur, and Sylhet. The highest rate of increase of 0.048 °C/yr was observed in Rangpur (Table S6 and Fig. S5).

##### 4.1.8.4 Winter or dry season

The share of stations showing increasing (54%) and decreasing (46%) trends is almost equal according to ITA. Significant increasing trends were observed in Chittagong, Cox’s Bazar, Dhaka, Khulna, M. Court, and Sylhet whereas Hatiya, Khepupara, Rajshahi, Rangamati, and Sandwip showed significant decreasing trends. Rangamati showed the highest rate of decrease of 0.08 °C/yr. The highest rate of increase was observed in Dhaka (0.049 °C/yr) (Table S6 and Fig. S5).

### 4.2 Sub-trend analysis

#### 4.2.1 Sub-trend analysis of annual rainfall

**Fig. 3:**
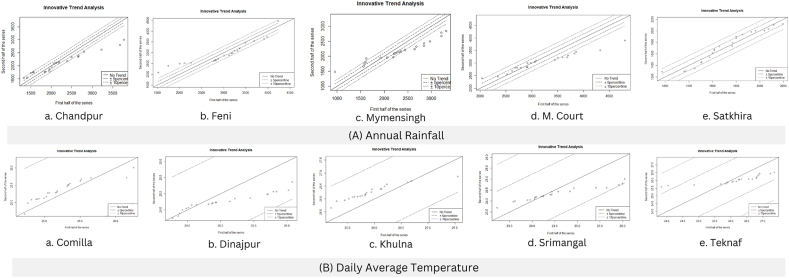
ITA plots of the stations showing sub-trend for (A) Annual rainfall at a. Chandpur, b. Feni, c. Mymensingh, d. M. Court, and e. Satkhira and (B) Daily average temperature at a. Comilla, b. Dinajpur, c. Khulna, d. Srimangal, e. Teknaf.

Rainfall above 2800 mm falls below and rainfall <1600 mm falls above the 10% line which implies Chandpur showed decreasing sub-trend for high intensity rainfall (>2800 mm) and increasing sub-trend for low intensity rainfall (<1600 mm). Feni showed increasing sub-trend for rainfall <2500 mm. Hatiya showed increasing sub-trend for high (>2500 mm) amount of annual precipitation which falls above 10% line. M. Court showed decreasing sub-trend for medium to high precipitation (3500 mm–5000 mm) which falls below 10% line but increasing sub-trend for low intensity rainfall (<2500 mm) which falls above 10% line. Mymensingh showed increasing sub-trend for low intensity (<1500 mm) rainfall. Satkhira showed decreasing sub-trend for rainfall intensity <1600 mm. The above-mentioned graphs are shown in Fig. 3 and rest of the graphs of the annual rainfall is presented in Fig. S6.

#### 4.2.2 Sub-trend analysis of seasonal rainfall

##### 4.2.2.1 Pre-monsoon

Although Bogra, Comilla, and Mymensingh showed negative trends, they showed increasing sub-trends for low intensity rainfall (<100 mm). Hatiya showed no trend, but it showed an increasing sub-trend for rainfall intensity of <150 mm, and a decreasing sub-trend for a rainfall intensity of >250 mm. Srimangal showed positive trend but has a decreasing sub-trend for high intensity rainfall (>300 mm).

##### 4.2.2.2 Monsoon

Dhaka showed an insignificant increasing trend but has a decreasing sub-trend for low intensity rainfall (200 mm–300 mm). Feni showed an increasing trend for rainfall intensity <400 mm but a decreasing trend for high intensity rainfall (>500 mm). Rajshahi showed negative trend but it has an increasing sub-trend for high intensity rainfall (>350 mm).

##### 4.2.2.3 Post-monsoon

Barisal, Bogra, Chandpur, Dhaka and Feni showed increasing trends but has decreasing sub-trends for high intensity rainfall (>200 mm). Although Cox’s Bazar showed a decreasing trend, it showed an increasing sub-trend for rainfall of an intensity <200 m. Mymensingh and Rajshahi showed negative trends for post monsoonal rainfall but have increasing sub-trend for rainfall intensity of >150 mm.

##### 4.2.2.4 Winter or dry season

Srimangal showed a decreasing trend but has an increasing sub-trend for rainfall intensity of >25 mm. Teknaf showed decreasing sub-trend for rainfall intensity of <30 mm.

#### 4.2.3 Sub-trend analysis of annual average temperature

From the analysis it is found that though Comilla showed an increasing trend, it has a decreasing sub trend for high annual temperature. Dinajpur showed an increasing sub trend for low annual temperature. Khulna, M. Court and Rangpur showed downward increasing trend which implies decreasing sub trend for high annual temperature. Srimangal and Teknaf showed increasing sub trend for low annual temperature <24.5 °C. The above-mentioned graphs are shown in Fig. 3 and rest of the graphs of the annual average temperature is presented in Fig. S7.

#### 4.2.4 Sub-trend analysis of seasonal average temperature

##### 4.2.4.1 Pre-monsoon

Dinajpur, Ishurdi, Patuakhali and Rajshahi showed increasing sub trends for low temperature (<27 °C). Faridpur and Khulna showed decreasing sub trends for high annual temperature >28 °C. Rangamati showed increasing sub trend for high annual temperature (>27.5 °C).

##### 4.2.4.2 Monsoon

Dhaka, Feni, Khepupara, M. Court, Rangamati and Rangpur showed downward increasing trends, implying decreasing sub trends for high annual temperature. Mymensingh, Satkhira and Srimangal showed decreasing sub trends for temperature >29 °C.

##### 4.2.4.3 Post-monsoon

Dinajpur showed an increasing sub trend for low temperature (<24 °C). Ishurdi showed an increasing trend for high temperature and a decreasing sub trend for low temperature. Khulna showed a decreasing sub trend for high temperature. Sandwip showed no trends for low temperature value.

##### 4.2.4.4 Winter or dry season

Barisal showed a decreasing sub-trend for low temperature (<20 °C) during the dry season. Faridpur showed an increasing sub-trend for high temperature >20 °C. Srimangal showed an increasing sub-trend for low temperature (<17 °C).

#### 4.2.5 Sub-trend analysis of annual maximum temperature

M. Court and Srimangal showed a downward trend, which implies, decreasing trends for higher maximum temperature >34 °C. Mymensingh showed decreasing sub trend for temperatures ranging from 31.5 °C to 32.5 °C.

#### 4.2.6 Sub-trend analysis of seasonal maximum temperature

##### 4.2.6.1 Pre-monsoon

Comilla and Satkhira showed decreasing sub-trend for temperatures ranging from 33 °C to 35.5 °C and 37 °C–38.5 °C respectively.

##### 4.2.6.2 Monsoon

Almost all the stations showed increasing trends for higher maximum temperature (33 °C–36 °C) during the monsoon season. The graphical representation for Bogra, Comilla, Ishurdi, M. Court and Teknaf showed decreasing sub trends for higher values of monsoonal rainfall.

##### 4.2.6.3 Post-monsoon

Barisal, Bogra, Comilla, Dhaka, Faridpur, M. Court, Patuakhali and Rangamati showed overall increasing trends. However, from the graphical representation indicates that they showed a downward trend line, implying decreasing sub-trends for higher maximum temperature >34.5 °C during the post-monsoon season.

##### 4.2.6.4 Winter or dry season

Bogra showed decreasing sub-trend for the temperature range of 29 °C–30.5 °C. M. Court and Srimangal showed a downward positive trend, implying increasing trends for lower maximum temperature <28 °C and decreasing sub-trend for higher maximum temperature >31 °C.

#### 4.2.7 Sub-trend analysis of annual minimum temperature

Chandpur, Chittagong, Dinajpur, Faridpur, Jessore, M. Court, Mymensingh, Rajshahi and Teknaf showed a downward positive trend indicating decreasing trend for higher minimum temperature.

#### 4.2.8 Sub-trend analysis of seasonal minimum temperature

##### 4.2.8.1 Pre-monsoon

Chandpur, Dinajpur, Feni, Jessore, M. Court and Mymensingh showed a downward positive trend line, implying that they show decreasing trend for higher minimum temperature.

##### 4.2.8.2 Monsoon

Chandpur, Cox’s Bazar, Chittagong, Faridpur, Feni, Jessore, Khepupara, Mymensingh, Patuakhali, Rangamati, Sylhet and Teknaf namely, the southern and eastern parts of the country, showed a downward positive trend line, implying that they have increasing trend for lower minimum temperature (<22 °C).

##### 4.2.8.3 Post-monsoon

Feni and Satkhira showed a downward positive trend, implying that they showed decreasing trend for higher minimum temperature (>18 °C). Khulna showed decreasing sub trend for higher minimum temperature >19 °C. Sandwip showed increasing sub trend for temperature >20 °C.

##### 4.2.8.4 Winter or dry season

Bogra showed an increasing sub-trend for temperature <9 °C. Comilla showed a decreasing sub-trend for medium to high minimum temperature (>10 °C).

### 4.3 SAI analysis

#### 4.3.1 SAI of average rainfall

The rainfall anomaly index for average rainfall was analyzed in 26 stations from 1975 to 2019. Almost all the stations, namely, Barisal, Bogra, Chandpur, Chittagong, Comilla, Faridpur, Feni, Ishurdi, Khulna, Patuakhali, Rajshahi, Rangamati, Satkhira, Srimangal, and Sylhet showed consecutive dry and wet years. Cox’s Bazar and Dhaka mostly showed wet years during the total time period and had extreme drought years in 1976, 1979, 1980, 2014 and 2019.

Wet years prevailed from 1983 to 2005 and dry years prevailed from 2006 to 2019 in Dinajpur. Wet years reigned from 1984 to 1988 and 2002 to 2008, whereas dry years prevailed from 1989 to 2001 and 2009 to 2019 in Jessore, which showed an increasing trend of dry years in recent times. Rajshahi mostly showed wet years from 1977 to 2000 but subsequently, dry years prevailed. Rangpur had wet years from 1984 to 2005 and dry years from 1975 to 1981 and 2006 to 2018. Sylhet also had severe to extreme warm years from 1992 to 2018.

Hatiya had dry and wet years from 1982 to 1994 and 1999 to 2017, respectively, but subsequently showed dry years in 2018 and 2019. Khepupara had dry and wet years from 1975 to 1989 and 1990 to 2019, respectivelt, wherein only 1992, 2009, and 2014 were extreme drought years. M. Court consecutively showed moderately wet and dry years from 1985 to 2016 but had extreme drought and wet years from 1978 to 1979 and 1979 to 1984, respectively.

**Fig. 4:**
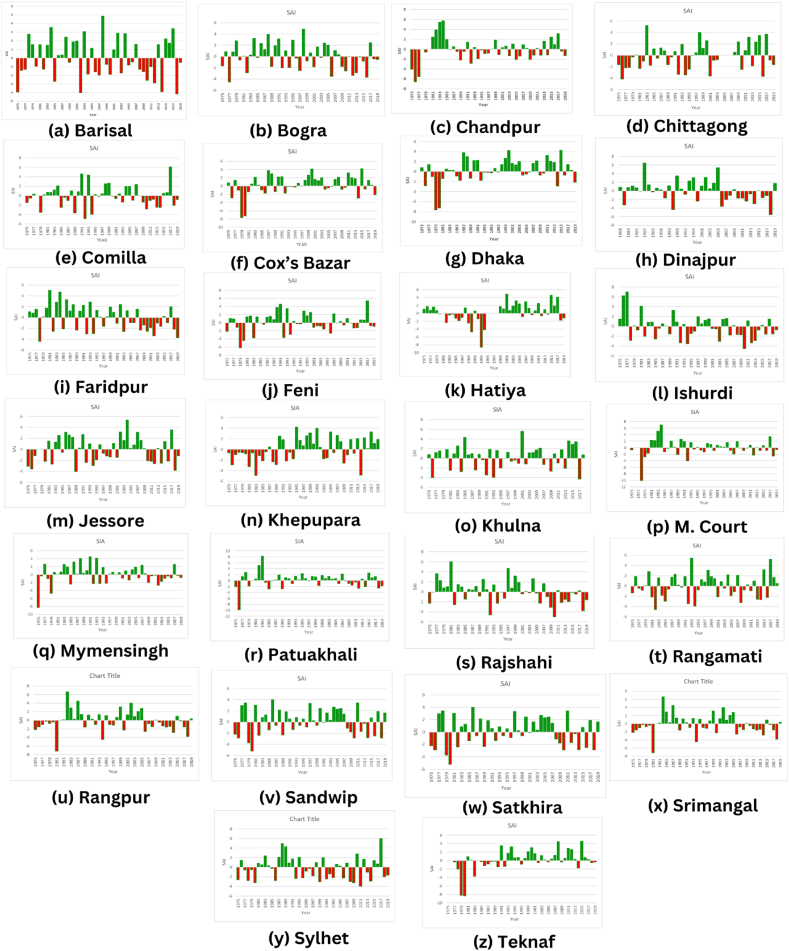
Standard anomaly index of average rainfall series from 1975 to 2019 of (a) Barisal, (b) Bogra, (c) Chandpur, (d) Chittagong, (e) Comilla, (f) Cox's Bazar, (g) Dhaka, (h) Dinajpur, (i) Faridpur, (j) Feni, (k) Hatiya, (l) Ishurdi, (m) Jessore, (n) Khepupara, (o) Khulna, (p) M. Court, (q) Mymensingh, (r) Patuakhali, (s) Rajshahi, (t) Rangamati, (u) Rangpur, (v) Sandwip, (w) Satkhira, (x) Srimangal, (y) Sylhet and (z) Teknaf.

Sandwip had dry and wet years from 1978 to 1997 and 1998 to 2018, respectively, indicating that Sandwip recently experienced wet years. Teknaf mostly experienced wet years from 1991 to 2017, and 1979, 1980, and 1983 were extreme drought years. All the graphs of SAI for average rainfall are shown in Fig. 4.

#### 4.3.2 SAI of average temperature

Temperature anomalies for the 26 stations were analyzed for the average, maximum, and mean temperatures from 1975 to 2019. From the analysis of average temperature, Barisal showed near normal to moderately warm years from 1975 to 2019. Feni intermittently showed moderate to severely warm years intermittently from 1980 to 2019. Hatiya showed two extremely cold years in 1983 and 1994 and eight extremely warm years during the studied period. Khepupara had severely warm years throughout the time period. Our analysis showed that Khulna, Patuakhali, and Rangamati mostly had moderately warm years during the total time period, whereas M. Court and Rangamati mostly had severely warm years.

Bogra and Chandpur had three extreme cold years from 1976 to 1979 and severe to extreme warm years intermittently from 2004 to 2019. Before 1991, Chittagong had few moderate to extreme cold years whereas after 1991, it had numerous moderate to extreme warm years. Comilla had 10 severely cold years from 1981 to 1997 and four extremely warm years from 2006 to 2016. Cox’s Bazar had extremely cold years before 1981 and moderately to severely warm years from 1995 to 2019.

Dhaka had severely cold and warm years before 1984 and after 2005, respectively. Jessore had few severely and extremely cold years up to 1997; subsequently, it had severely to extremely warm years between 2005 and 2017. Sandwip had four extremely cold years in 1976, 1979, 1991, 2002, and fourteen extremely warm years intermittently; severely warm years were prevalent during the other years.

Srimangal had extreme cold years during 1975–1978, 1983, and 1985, and extreme warm years in 1979, 1984, 1986, 1987, 1988, 1999, 2006, and 2016; moderately warm years occurred in other years of the studied period. Sylhet had moderate to severe cold years from 1976 to 1993 and subsequently had moderate to severe warm years. Teknaf had only one extreme cold year in 1980 and thirteen extremely warm years; the remaining period was characterized by moderate to severe warm years.

Dinajpur showed moderately cold years between 1989 and 2019. Satkhira had one extremely cold year in 1980 and extremely warm years from 1979 to 1988; subsequently, it had severely cold years. Mymensingh had extremely warm years from 1980 to 1986; subsequently, it had severely to extremely cold years.

Faridpur showed intermittent moderately warm and cold years. Ishurdi showed alternating cold and warm years, wherein only eight extremely warm years occurred and most of them occurred between 1999 and 2017. Rajshahi had extremely cold years in 1981, 1983, 1985, 1990, 1997, and 2011 and extremely warm years in 1975, 1976, 1979, 1999, 2010, and 2016.

Notably, most stations experienced cold years from 1975 to 1985, except for Comilla, Dinajpur, Jessore, Mymensingh, Rajshahi, and Satkhira; subsequently, warm years prevailed. All the graphs of SAI for average temperature are showed in Fig. S8.

#### 4.3.3 SAI of maximum temperature

The SAI of maximum temperature data followed the similar pattern of average annual temperature at most stations, except for Bogra, Chandpur, Mymensingh, Rajshahi, Rangpur, and Sandwip wherein warm years were observed throughout the studied period. Dinajpur and Dhaka had alternating warm and cold years.

#### 4.3.4 SAI of minimum temperature

The SAI of minimum temperature data showed that almost all stations experienced cold and warm years during the first and last two decades, respectively. Exceptions were observed in Faridpur, Hatiya, Mymensingh, Patuakhali, Rangamati, Rangpur, and Sandwip wherein warm years occurred throughout the studied period. Jessore showed alternating warm and cold years.

### 4.4 Analysis of extreme climate indices

As some of the indices of the ETCCDI are relevant to the sector and location, we chose the most relevant indices for analysis. Prior to the performance of the extreme climatic indices, the homogeneity and quality of the daily input data were checked. For this analysis, the data of eight divisions namely, Barisal, Chittagong, Dhaka, Khulna, Mymensingh, Rajshahi, Rangpur and Sylhet of Bangladesh were used. The analysis showed that all the daily data of all the eight stations were homogenous and they needed no further adjustment for the extreme event analysis which is in consistent to the findings of Shahid [45] and Rahman et al. [18]. The result of extreme climate indices analysis using ClimPACT2 software is presented in Table 4.

**Table 4:** Temperature and precipitation extreme indices values.

Indicator		Barisal	Chittagong	Dhaka	Khulna	Mymensingh	Rajshahi	Rangpur	Sylhet
Temperature Indices									
**WSDI (days/year)**	Trend	0.051	0.139	0.101	0.083	0.016	−0.043	0.058	0.25
	*p*-value	0.468	0.228	0.196	0.344	0.716	0.574	0.446	0.002
**CSDI (days/year)**	Trend	−0.108	−0.138	−0.127	0.302	−0.196	−0.025	−0.179	−0.371
	*p*-value	0.127	0.163	0.02	0.1	0.266	0.626	0.008	0.016
**TXx (°C)**	Trend	0.015	0.016	−0.016	−0.003	−0.004	−0.025	−0.029	0.054
	*p*-value	0.214	0.331	0.284	0.887	0.75	0.234	0.07	0
**TNn (°C)**	Trend	−0.003	0.065	0.045	0.07	0.017	−0.095	−0.009	0.04
	*p*-value	0.878	0.021	0.007	0.043	0.552	0.007	0.784	0.193
**TR (days/year)**	Trend	0.348	0.582	0.515	0.244	0.237	0.104	0.512	0.697
	*p*-value	0.003	0	0	0.072	0.117	0.359	0	0
**SU25 (days/year)**	Trend	0.229	0.089	0.094	−0.034	−0.125	−0.033	0.219	0.588
	*p*-value	0.039	0.475	0.446	0.779	0.377	0.786	0.22	0
**GSL (days/year)**	Trend	0.021	0.003	0.003	−0.001	−0.008	−0.003	−0.001	−0.004
	*p*-value	0.134	0.665	0.739	0.836	0.252	0.67	0.916	0.455
**DTR (°C)**	Trend	0.005	−0.018	−0.012	−0.006	−0.012	0.007	−0.014	0.013
	*p*-value	0.323	0.016	0.058	0.369	0.078	0.315	0.008	0.024
**TX10p (%)**	Trend	−0.152	−0.046	−0.088	−0.08	0.005	−0.086	−0.042	−0.186
	*p*-value	0	0.357	0.013	0.06	0.905	0.01	0.253	0
**TX90p (%)**	Trend	0.283	0.298	0.202	0.199	0.132	0.221	0.25	0.484
	*p*-value	0	0.007	0.003	0	0.004	0	0	0
**TN10p (%)**	Trend	−0.225	−0.304	−0.296	−0.113	−0.232	−0.137	−0.3	−0.412
	*p*-value	0	0	0	0.13	0.028	0.028	0	0
**TN90p (%)**	Trend	0.222	0.383	0.286	0.258	0.122	0.065	0.198	0.33
	*p*-value	0.001	0	0	0	0.031	0.269	0	0
Rainfall Indices									
**R20mm (days/year)**	Trend	−0.084	0.118	−0.119	0.06	−0.035	−0.089	−0.109	0.012
	*p*-value	0.326	0.129	0.168	0.47	0.749	0.168	0.192	0.929
**R10mm (days/year)**	Trend	−0.216	0.175	−0.068	0.058	−0.14	−0.106	−0.187	−0.058
	*p*-value	0.032	0.057	0.523	0.6	0.301	0.215	0.103	0.701
**RX3days (mm)**	Trend	0.943	0.247	−0.662	−0.667	−0.274	−1.682	−0.099	0.067
	*p*-value	0.337	0.897	0.511	0.61	0.783	0.046	0.936	0.55
**CDD (days/year)**	Trend	0.579	0.575	0.58	0.677	0.802	0.684	1.008	0.748
	*p*-value	0.074	0.169	0.048	0.036	0.026	0.042	0.014	0.016
**PRCPTOT (mm)**	Trend	−4.011	5.952	−7.835	0.243	−7.19	−8.663	−7.977	−3.435
	*p*-value	0.364	0.35	0.131	0.956	0.256	0.012	0.171	0.681
**R95pTOT (%)**	Trend	0.097	−0.009	−0.212	−0.004	−0.094	−0.255	−0.153	−0.11
	*p*-value	0.478	0.95	0.123	0.979	0.413	0.039	0.27	0.171
**R99pTOT (%)**	Trend	0.005	−0.044	−0.071	−0.003	−0.05	−0.171	−0.016	−0.092
	*p*-value	0.96	0.745	0.443	0.978	0.555	0.086	0.862	0.19
**SPI**	Trend	−0.001	0.001	−0.002	0	0	−0.002	−0.002	−0.001
	*p*-value	0	0.001	0	0.893	0.204	0	0	0.033
**CWD (days/year)**	Trend	−0.026	0.005	−0.079	0.045	−0.001	−0.013	−0.071	−0.133
	*p*-value	0.698	0.923	0.1	0.464	0.983	0.68	0.208	0.189

N·B.: statistically significant results when p < 0.05.

Our analysis showed that the results of the indices related to temperature are more statistically significant (*p* < 0.05) at a CL of 95% compared with those of the rainfall indices at the stations. Sylhet showed a significant increasing trend for the warm spell duration index (WSDI), which implies that consecutive days of higher temperature are increasing in Sylhet at a rate of 0.25 days/yr. Dhaka, Rangpur, and Sylhet showed significant decreasing trends for the cold spell duration index (CSDI), which implies that the occurrence of consecutive cold days is declining.

Sylhet showed a significant increasing trend for the highest maximum temperature (TXx) which implies that Sylhet is experiencing more warmest days at a rate of 0.054 °C/yr. Chittagong, Dhaka, and Khulna showed significant increasing trends for the lowest minimum temperature (TNn) which implies that these stations are experiencing an increase in coldest days and Rajshahi is experiencing the opposite. Barisal, Chittagong, Dhaka, Rangpur, and Sylhet showed increasing trends for tropical nights (TR), which implies an increase in the occurrence of warmer nights. Barisal and Sylhet showed significant increasing trends for the annual counts of days when daily maximum temperature exceeded 25 °C (SU25), which implies that they are experiencing more annual number of days where TX > 25 °C.

Barisal, Chittagong, and Dhaka showed increasing growing season lengths (GSL) but none of these are statistically significant. Chittagong and Rangpur showed significant decreasing rates for the diurnal temperature range (DTR), which implies that the increase of minimum temperature is higher than that of maximum temperature; Sylhet showed an increasing rate for DTR (0.013 °C/yr) which indicates that the increase of maximum temperature is greater than that of minimum temperature.

Barisal, Dhaka, Rajshahi, and Sylhet showed significant decreasing trends for the percentage of days when TX < 10th percentile (TX10p) and increasing trends for the percentage of days when TX < 90th percentile (TX90p), which indicates that the days with maximum temperature are increasing at these stations. Chittagong, Khulna, Mymensingh, and Rangpur also showed significant increasing trends for TX90p. Barisal, Chittagong, Dhaka, Rangpur, and Sylhet showed significant decreasing trends for cold nights and increasing trends for hot nights.

The analysis of rainfall indices showed less significant trends, and the majority of them show decreasing trends. Barisal showed a significant decreasing trend for the number of heavy precipitation days (R10mm) indicating a decreasing trend for heavy rain days.

Rajshahi showed a significant decreasing trend for the amount of consecutive precipitation or maximum 3-day total precipitation (Rx3day). Dhaka, Khulna, Mymensingh, Rajshahi, Rangpur, and Sylhet showed significant increasing trends for consecutive dry days.

Considering the annual total wet days, all the stations showed decreasing trends, wherein only Rajshahi showed a significantly decreasing trend. Considering the contribution from very and extremely wet days, all the stations showed decreasing trends, wherein Rajshahi showed a significantly decreasing trend. The overall findings suggest that the country has been observing hotter and dryer weather with time which is in line to the previous studies undertaken by Abdullah et al. [46], Tuladhar et al. [47].

The SPI index is one of the most widely used indices to track drought [48]. All the stations showed negative trends for the standardized precipitation index, and among which Barisal, Dhaka, Rajshahi, Rangpur, and Sylhet showed significant drought conditions. Considering consecutive wet days, all the stations showed statistically insignificant trends.

## 5 Discussion

Our analysis indicated that the MK, mMK and ITA results were almost similar for both rainfall and temperature data; however, among them, the mMK and ITA results were more identical, suggesting that ITA provides more precise values and also helps to identify the sub-trends at any stations for both rainfall and temperature data. Wu and Qian [49] and Girma et al. [50] also found ITA to be reliable and consistent as MK and Sen’s slope estimators.

The analysis of rainfall data indicated that the south-western, southern central, south-eastern and eastern sides of the country showed increasing trends and western, northern and central parts of the country showed decreasing trends. Cox’s Bazar, Hatiya, Sandwip, and Teknaf, namely, the coastal area of Bangladesh, showed notable increasing trends for the annual and seasonal rainfall. The central part and Sylhet showed the highest decreasing trends for annual rainfall.

Das et al. [2] also concluded that the increasing trend in rainfall is observed in the south eastern, south western and north eastern parts of Bangladesh. Rahman et al. [18] found increasing rainfall trends in Cox’s Bazar, Khulna, and Satkhira and decreasing trends in Srimangal. Rahman and Lateh [19] found that dry conditions dominated in the north western, western and south western parts of the country.

The analysis of pre-monsoon season rainfall showed decreasing trends in the central part of the country. During the monsoon season, the northern part of the country showed a severely decreasing trend in rainfall. During the post-monsoon season, the north eastern part of Bangladesh showed a decreasing trend in rainfall, and during the dry season, all the stations showed decreasing trends. Shahid [51] analyzed the rainfall data of 17 stations from 1958 to 2007 and found increasing trends in the annual and pre-monsoon season rainfall. This is in contrast to our overall findings but it also indicates that the rainfall pattern has changed in the last two decades.

The northern and central parts of the country showed significant increasing trends for the annual temperature. The increase in maximum temperature is more prominent than that in minimum temperature. The maximum temperature is increasing in the eastern, central, and coastal areas of Bangladesh. The temperature increases during the monsoon season and decreases during the dry season it is decreasing. The coastal and hilly areas of Bangladesh showed decreasing trends in temperature during the pre-monsoon and post-monsoon seasons.

Khan et al. [16] found that the daily temperature showed warming trends in all regions and the rate of increase was higher in monsoon. Bhuyan et al. [21] found an increasing temperature trend in the north western region of Bangladesh. Moreover, studies conducted in the neighboring areas [12,52,53] have also found increasing trends in temperature throughout the respective study periods and study areas, but the trends of rainfall are erratic and inhomogeneous in nature.

The sub-trend analysis showed that the stations showing overall positive trends, showed decreasing sub-trend for medium to high intensity rainfall. Considering the temperature, the sub-trend analysis showed that lower maximum and minimum temperature are increasing, which has minimized the seasonal variation of temperature, resulting in a warmer weather throughout the year.

The SAI analysis for rainfall data revealed that almost all the stations showed consecutive dry and wet years. Cox’s Bazar and Dhaka mostly had wet years from 1975 to 2019 and extreme drought years in 1976, 1979, 1980, 2014, and 2019. Jessore, Rajshahi, and Rangpur had moderate to severe drought years in the last two decades. Teknaf, Sandwip, Khepupara, and Hatiya namely, the coastal areas of Bangladesh, experienced moderate to severe wet years in the last two decades. SAI analysis for temperature data revealed that almost all parts of Bangladesh showed moderate to severe warm years in the last two decades. The majority of the stations experienced cold years during the first two decades; subsequently, all of them began to experience warm years. The coastal area of the country experienced warm years throughout the studied period.

The overall result of the analysis of extreme indices suggests that Sylhet is vulnerable to increasing temperature and drought conditions as hot days, tropical nights, daily maximum temperature, percent hot days and nights and consecutive dry days show growing trends. Chittagong, Dhaka, Rangpur, and Khulna experience fewer cold days or minimum temperatures. Barisal and Rajshahi are susceptible to significant drought conditions due to decreasing trend in consecutive wet days, annual total wet days, number of heavy rain days.

The limitation of our study is it relies on restricted number of meteorological stations due to data unavailability of all of them which might create some gap in weather patterns analysis throughout Bangladesh. In addition, analysis of other climatic parameters i.e., wind speed, humidity, sunshine etc. could have shown underlying relationship among them influencing the observed trends which has not been undertaken in this study. Thus, future studies using other climatic parameters using different trend detection techniques for both observed and simulated data is recommended.

## 6 Conclusion

In this study, we aimed to identify the trends and patterns of annual and seasonal climate change variables and the occurrence of extreme events throughout Bangladesh using accepted statistical analysis. Our analysis showed that during the last four decades, the pattern and trend of climate variables had heterogeneously. Both increasing and decreasing trends of annual rainfall were observed, but the majority stations showed decreasing trends. The central part of Bangladesh showed significant decreasing trends for rainfall.

The maximum temperature is increasing in the eastern, central, and coastal areas of Bangladesh. The rainfall and maximum temperature are inversely related during the monsoon and dry seasons as the rainfall and temperature show decreasing (0.65 mm/yr) and increasing (0.017 °C/yr) trends, respectively. The pre- and post-monsoonal rainfall values also reflect the decreasing trends, indicating prevailing drought conditions, especially in the northern and central parts of the country.

In the past 20 years, the country's western region experienced more drought years, whereas the coastal region experienced more wet years. The majority of the stations experienced cold years from 1975 to 1995; subsequently, warm years prevailed. Exceptions was observed at Dinajpur, Mymensingh, and Satkhira. Our analysis showed that the results of the indices related to temperature were more statistically significant (*p* < 0.05) at a CL of 95% compared with those of the rainfall indices at the eight stations.

The results of our study can aid in understanding regional climate change in South Asian regions, and help define appropriate policies and plans to mitigate the effects of climate change in Bangladesh. On regional and national scales, the trends of temperature and rainfall, and the analyses of sub-trend directions, magnitudes, extreme indices, and spatial patterns may also provide useful information on global warming and drought conditions which is important in the field of agriculture, ecosystem, and water availability. Moreover, we expected that this study will contribute toward the management of water resources, agricultural output, food security, and standard of living for the general populace in Bangladesh.

## Funding

This research received no funding grant from any funding agency.

## Data availability

The datasets generated during and/or analyzed during the current study are available at https://data.mendeley.com/preview/n35gn9w8nc (https://doi.org/10.17632/n35gn9w8nc.1).

## CRediT authorship contribution statement

**Shanjana Haider:** Writing – review & editing, Writing – original draft, Software, Methodology, Formal analysis, Data curation, Conceptualization. **Md Rezaul Karim:** Writing – review & editing, Supervision, Resources, Methodology, Conceptualization. **Md Saiful Islam:** Writing – review & editing, Writing – original draft, Methodology, Formal analysis, Conceptualization. **Tanzilla Aktar Megumi:** Software, Formal analysis. **Quazi Shahnewaz Rahnama:** Formal analysis.

## Declaration of competing interest

The authors declare that they have no known competing financial interests or personal relationships that could have appeared to influence the work reported in this paper.
