# Supplementary material for: Extreme weather events and Spatio-temporal characterization of climate change variables in Bangladesh during 1975–2019
Source: Heliyon. 2024 Feb 27;10(5):e27118. doi: 10.1016/j.heliyon.2024.e27118 (PMC10918194; doi:10.1016/j.heliyon.2024.e27118)
Supplement: Multimedia component 1 [file mmc1.docx]

**Extreme Weather Events and Spatio-temporal Characterization of Climate Change Variables in Bangladesh during 1975 – 2019**

**Shanjana Haider^1, *^, Md. Rezaul Karim^1^, Md. Saiful Islam^1, *^, Tanzilla Aktar Megumi^1^, and Quazi Shahnewaz Rahnama^1^**

^1^ Department of Civil and Environmental Engineering, Islamic University of Technology (IUT), Gazipur 1704, Bangladesh.

*Corresponding author.

E-mail address: saifulislam2@iut-dhaka.edu (Md. Saiful Islam)

shanjanahaider@iut-dhaka.edu (Shanjana Haider)

**Supplementary Material**

**Table S1: SAI value classification**

| Rainfall | | Temperature | |
| --- | --- | --- | --- |
| SAI value | **Category** | **SAI value** | **Category** |
| > 2.0 | Extremely Wet | > 2.0 | Extremely Warm |
| 1.5 to 1.99 | Severely Wet | 1.5 to 1.99 | Severely Warm |
| 1.0 to 1.49 | Moderately Wet | 1.0 to 1.49 | Moderately Warm |
| -0.99 to 0.99 | Near Normal | -0.99 to 0.99 | Near Normal |
| -1.0 to -1.49 | Moderately Dry | -1.0 to -1.49 | Moderately Cold |
| -1.5 to -1.99 | Severely Dry | -1.5 to -1.99 | Severely Cold |
| < 2.0 | Extremely dry | < 2.0 | Extremely Cold |

**Table S2: Details of extreme temperature and precipitation indices**

| **S N.** | **Indicator** | **Long Name** | **Definition** | **Unit** |
| --- | --- | --- | --- | --- |
|  | WSDI | Warm spell duration  indicator | Annual number of days with at least 6 consecutive days when TX > 90th percentile | days |
|  | CSDI | Cold spell duration indicator | Annual number of days with at least 6 consecutive days when TN < 10th percentile | days |
|  | TXx | Max TX | Warmest daily TX | °C |
|  | TNn | Min TN | Coldest daily TN | °C |
|  | TR | Tropical nights | Annual number of days when TN > 20 °C | days |
|  | SU25 | Hot days | Annual number of days when TX >= 25 °C | days |
|  | CDD | Consecutive dry days | Maximum annual number of consecutive dry days (when PR < 1.0 mm) | days |
|  | R20mm | Number of very heavy rain days | Annual number of days when PR >= 20 mm | days |
|  | RX3days | 3 consecutive days  PR amount | Maximum consecutive 3-day total precipitation | mm |
|  | PRCPTOT | Annual total wet days PR | Annual sum of daily PR >= 1.0 mm | mm |
|  | R95pTOT | Contribution from very wet days | 100*r95p / PRCPTOT | % |
|  | R99pTOT | Contribution from extremely wet days | 100*r99p / PRCPTOT | % |
|  | GSL | Growing season Length | Annual number of days between the first occurrence of 6 consecutive days with TM > 5 °C and the first occurrence of 6 consecutive days with TM < 5 °C | days |
|  | SPI | Standardized Precipitation Index | Measure of “drought” using the Standardized Precipitation Index on 12-month time scale. | unitless |
|  | DTR | Daily temperature range | Mean difference between daily TX and daily TN | °C |
|  | TX10p | Percentage of cool days | Percentage of days when TX < 10th percentile | % |
|  | TX90p | Percentage of hot days | Percentage of days when TX > 90th percentile | % |
|  | TN10p | Percentage of cold nights | Percentage of days when TN < 10th percentile | % |
|  | TN90p | Percentage of hot nights | Percentage of days when TN > 90th percentile | % |
|  | CWD | Consecutive wet days | Maximum annual number of consecutive wet days (when PR >= 1.0 mm) | days |
|  | R10mm | Number of heavy rain days | Annual number of days when PR >= 10 mm | days |

**Table S3: Mean, Standard Deviation, Variance and CV of rainfall data of all the stations**

| **STATIONS** | **Mean** | **Standard Deviation** | **Variance** | **CV** | **CV in %** |
| --- | --- | --- | --- | --- | --- |
| Barisal | 2084.889 | 358.0104253 | 128171.5 | 0.171717 | 17.17168 |
| Bogra | 1735.8 | 382.2661852 | 146127.4 | 0.220225 | 22.02248 |
| Chandpur | 2158.489 | 553.3998481 | 306251.4 | 0.256383 | 25.6383 |
| Chittagong | 2938.667 | 502.5996509 | 252606.4 | 0.17103 | 17.10298 |
| Comilla | 2059.289 | 400.0262277 | 160021 | 0.194255 | 19.42545 |
| Cox's Bazar | 3625.333 | 691.294175 | 477887.6 | 0.190684 | 19.06843 |
| Dhaka | 2050.4 | 450.8060659 | 203226.1 | 0.219862 | 21.98625 |
| Dinajpur | 1958.644 | 456.9086419 | 208765.5 | 0.233278 | 23.3278 |
| Faridpur | 1800.556 | 340.9409143 | 116240.7 | 0.189353 | 18.93532 |
| Feni | 2981.733 | 622.3862218 | 387364.6 | 0.208733 | 20.8733 |
| Hatiya | 3276.111 | 679.2737172 | 461412.8 | 0.207341 | 20.73415 |
| Ishurdi | 1556.222 | 378.467237 | 143237.4 | 0.243196 | 24.31961 |
| Jessore | 1665.678 | 324.214333 | 105114.9 | 0.194644 | 19.4644 |
| Khepupara | 2791 | 380.5196208 | 144795.2 | 0.136338 | 13.63381 |
| Khulna | 1836.044 | 345.8280547 | 119597 | 0.188355 | 18.83549 |
| M.Court | 3107.289 | 532.0580378 | 283085.8 | 0.171229 | 17.1229 |
| Mymensingh | 2233.222 | 498.8784644 | 248879.7 | 0.22339 | 22.33895 |
| Patuakhli | 2625.333 | 474.2620104 | 224924.5 | 0.180648 | 18.06483 |
| Rajshahi | 1435.444 | 298.3386998 | 89005.98 | 0.207837 | 20.78372 |
| Rangamati | 2587.044 | 553.8595143 | 306760.4 | 0.21409 | 21.40897 |
| Rangpur | 2235.933 | 467.3025786 | 218371.7 | 0.208997 | 20.89967 |
| Sandwip | 3675.067 | 792.8345408 | 628586.6 | 0.215733 | 21.57334 |
| Satkhira | 1721.8 | 268.3672518 | 72020.98 | 0.155864 | 15.58644 |
| Srimangal | 2398.467 | 496.5724153 | 246584.2 | 0.207037 | 20.70374 |
| Sylhet | 4093.467 | 653.8902327 | 427572.4 | 0.15974 | 15.974 |
| Teknaf | 4168.6 | 782.465143 | 612251.7 | 0.187705 | 18.77045 |

**Table S4: Mean, Standard Deviation, Variance and CV of maximum temperature data of all the stations**

| **STATIONS** | **Mean** | **Standard Deviation** | **Variance** | **CV** | **CV in %** |
| --- | --- | --- | --- | --- | --- |
| Barisal | 33.62907 | 0.424846311 | 0.180494 | 0.012633 | 1.26333 |
| Bogra | 34.25944 | 0.41450043 | 0.171811 | 0.012099 | 1.209887 |
| Chandpur | 33.43148 | 0.61536228 | 0.378671 | 0.018407 | 1.840667 |
| Chittagong | 33.14978 | 0.610208079 | 0.372354 | 0.018408 | 1.84076 |
| Comilla | 33.44815 | 0.399616644 | 0.159693 | 0.011947 | 1.194735 |
| Cox's Bazar | 33.24824 | 0.830278794 | 0.689363 | 0.024972 | 2.497211 |
| Dhaka | 33.91389 | 0.534598405 | 0.285795 | 0.015763 | 1.576341 |
| Dinajpur | 33.68056 | 0.536446289 | 0.287775 | 0.015927 | 1.592748 |
| Faridpur | 33.96852 | 0.533936357 | 0.285088 | 0.015719 | 1.571856 |
| Feni | 33.63074 | 0.395547434 | 0.156458 | 0.011761 | 1.176148 |
| Hatiya | 32.76 | 0.610786874 | 0.373061 | 0.018644 | 1.864429 |
| Ishurdi | 34.61315 | 0.450702625 | 0.203133 | 0.013021 | 1.302114 |
| Jessore | 34.98444 | 0.516164223 | 0.266426 | 0.014754 | 1.475411 |
| Khepupara | 33.19963 | 0.700218571 | 0.490306 | 0.021091 | 2.109116 |
| Khulna | 34.45259 | 0.492608953 | 0.242664 | 0.014298 | 1.429817 |
| M.Court | 33.42741 | 0.60689073 | 0.368316 | 0.018155 | 1.815548 |
| Mymensingh | 33.37667 | 0.382317495 | 0.146167 | 0.011455 | 1.145463 |
| Patuakhli | 33.71593 | 0.647189003 | 0.418854 | 0.019195 | 1.919535 |
| Rajshahi | 34.76537 | 0.516312059 | 0.266578 | 0.014851 | 1.485133 |
| Rangamati | 33.82 | 0.615430272 | 0.378754 | 0.018197 | 1.819723 |
| Rangpur | 33.20537 | 0.362629094 | 0.1315 | 0.010921 | 1.09208 |
| Sandwip | 32.71549 | 0.863899701 | 0.746323 | 0.026406 | 2.640644 |
| Satkhira | 34.59981 | 0.490417934 | 0.24051 | 0.014174 | 1.4174 |
| Srimangal | 33.86722 | 0.458252059 | 0.209995 | 0.013531 | 1.353084 |
| Sylhet | 33.75833 | 0.687326654 | 0.472418 | 0.02036 | 2.036021 |
| Teknaf | 31.42934 | 0.531768693 | 0.282778 | 0.016919 | 1.69195 |

**Table S5: Mean, Standard Deviation, Variance and CV of minimum temperature data of all the stations**

| **STATIONS** | **Mean** | **Standard Deviation** | **Variance** | **CV** | **CV in %** |
| --- | --- | --- | --- | --- | --- |
| Barisal | 17.63333 | 0.678242306 | 0.460013 | 0.038464 | 3.846365 |
| Bogra | 17.45907 | 0.609180663 | 0.371101 | 0.034892 | 3.489192 |
| Chandpur | 18.61667 | 0.706061419 | 0.498523 | 0.037926 | 3.792631 |
| Chittagong | 18.63815 | 0.75484439 | 0.56979 | 0.0405 | 4.049997 |
| Comilla | 17.70389 | 0.46905369 | 0.220011 | 0.026494 | 2.649439 |
| Cox's Bazar | 19.38519 | 0.613209208 | 0.376026 | 0.031633 | 3.163288 |
| Dhaka | 18.17926 | 0.618199421 | 0.382171 | 0.034006 | 3.400575 |
| Dinajpur | 16.47667 | 0.670908851 | 0.450119 | 0.040719 | 4.071872 |
| Faridpur | 17.7013 | 0.481465027 | 0.231809 | 0.027199 | 2.719942 |
| Feni | 17.87741 | 0.606466683 | 0.367802 | 0.033924 | 3.392364 |
| Hatiya | 18.64741 | 0.618455182 | 0.382487 | 0.033166 | 3.316575 |
| Ishurdi | 16.65 | 0.562560322 | 0.316474 | 0.033787 | 3.378741 |
| Jessore | 17.13315 | 0.470826592 | 0.221678 | 0.02748 | 2.748045 |
| Khepupara | 18.40796 | 0.550814238 | 0.303396 | 0.029923 | 2.992261 |
| Khulna | 18.0413 | 0.570522077 | 0.325495 | 0.031623 | 3.162312 |
| M.Court | 18.68914 | 0.631539463 | 0.398842 | 0.033792 | 3.379179 |
| Mymensingh | 17.36463 | 0.532898602 | 0.283981 | 0.030689 | 3.068874 |
| Patuakhli | 18.54019 | 0.474575464 | 0.225222 | 0.025597 | 2.559713 |
| Rajshahi | 16.80204 | 0.536605597 | 0.287946 | 0.031937 | 3.193694 |
| Rangamati | 17.91296 | 0.768382787 | 0.590412 | 0.042895 | 4.289535 |
| Rangpur | 16.75704 | 0.598393144 | 0.358074 | 0.03571 | 3.570996 |
| Sandwip | 18.64204 | 0.613021463 | 0.375795 | 0.032884 | 3.288382 |
| Satkhira | 17.69574 | 0.517016689 | 0.267306 | 0.029217 | 2.921701 |
| Srimangal | 15.98556 | 0.579640367 | 0.335983 | 0.03626 | 3.626026 |
| Sylhet | 17.47389 | 0.633501772 | 0.401324 | 0.036254 | 3.625419 |
| Teknaf | 18.98887 | 0.501820576 | 0.251824 | 0.026427 | 2.642708 |

**Table S6: Details of annual and seasonal minimum temperature trend in Bangladesh**

| Stations | Annual | | | | | Pre-monsoon | | | | | Monsoon | | | | | Post-monsoon | | | | | Dry | | | | |
| --- | --- | --- | --- | --- | --- | --- | --- | --- | --- | --- | --- | --- | --- | --- | --- | --- | --- | --- | --- | --- | --- | --- | --- | --- | --- |
|  | Z statistics (MK) | Sen's Slope | Tau (mMK) | ITASlope | ITA Trend | Z statistics (MK) | Sen's Slope | Tau (mMK) | ITASlope | ITA Trend | Z statistics (MK) | Sen's Slope | Tau (mMK) | ITA Slope | ITA Trend | Z statistics (MK) | Sen's Slope | Tau (mMK) | ITA Slope | ITA Trend | Z statistics (MK) | Sen's Slope | Tau (mMK) | ITA Slope | ITA Trend |
| Barisal | 3.727** | 0.024 | 0.444 | 0.031 | **↑** | 2.653** | 0.029 | 0.275 | 0.021 | **↑** | 3.903** | 0.038 | 0.484 | 0.048 | **↑** | 1.204 | 0.014 | 0.125 | 0.036 | **↑** | 1.253 | 0.017 | 0.130 | 0.015 | **↑** |
| Bogra | 2.768** | 0.022 | 0.331 | 0.028 | **↑** | 3.318** | 0.033 | 0.343 | 0.032 | **↑** | 4.197** | 0.038 | 0.504 | 0.045 | **↑** | 1.057 | 0.019 | 0.110 | 0.031 | **↑** | -0.176 | -0.004 | -0.019 | -0.001 | **↓** |
| Chandpur | 2.690** | 0.019 | 0.317 | 0.028 | **↑** | 1.321 | 0.018 | 0.177 | 0.029 | **↑** | 3.788** | 0.016 | 0.392 | 0.029 | **↑** | 2.084* | 0.036 | 0.216 | 0.040 | **↑** | 1.683 | 0.014 | 0.175 | 0.019 | **↑** |
| Chittagong | 4.451** | 0.043 | 0.531 | 0.033 | **↑** | 3.610** | 0.053 | 0.486 | 0.040 | **↑** | 3.844** | 0.049 | 0.464 | 0.036 | **↑** | 2.320* | 0.030 | 0.240 | 0.035 | **↑** | 2.750** | 0.030 | 0.285 | 0.020 | **↑** |
| Comilla | 3.131** | 0.016 | 0.324 | 0.017 | **↑** | 1.664 | 0.017 | 0.173 | 0.022 | **↑** | 2.906** | 0.017 | 0.301 | 0.017 | **↑** | 1.419 | 0.025 | 0.147 | 0.031 | **↑** | 0.225 | 0.001 | 0.024 | 0.002 | **↑** |
| Cox's Bazar | 3.512** | 0.029 | 0.426 | 0.022 | **↑** | 3.269** | 0.033 | 0.338 | 0.031 | **↑** | 3.355** | 0.028 | 0.394 | 0.020 | **↑** | 1.657 | 0.015 | 0.172 | 0.020 | **↑** | 2.702** | 0.029 | 0.280 | 0.017 | **↑** |
| Dhaka | 3.786** | 0.034 | 0.518 | 0.030 | **↑** | 2.759** | 0.041 | 0.381 | 0.038 | **↑** | 1.477 | 0.015 | 0.242 | 0.008 | **↑** | 2.946** | 0.031 | 0.305 | 0.042 | **↑** | 2.201* | 0.049 | 0.397 | 0.043 | **↑** |
| Dinajpur | 3.003** | 0.022 | 0.340 | 0.033 | **↑** | 2.944** | 0.040 | 0.345 | 0.053 | **↑** | 3.776** | 0.043 | 0.515 | 0.051 | **↑** | 1.605 | 0.019 | 0.167 | 0.030 | **↑** | -1.038 | -0.010 | -0.108 | -0.009 | **↓** |
| Faridpur | 3.003** | 0.018 | 0.353 | 0.026 | **↑** | 3.424** | 0.042 | 0.398 | 0.043 | **↑** | 4.825** | 0.036 | 0.499 | 0.045 | **↑** | -0.764 | -0.012 | -0.080 | 0.003 | **↑** | -1.233 | -0.013 | -0.128 | -0.003 | **↓** |
| Feni | 1.986* | 0.016 | 0.284 | 0.025 | **↑** | 2.104* | 0.023 | 0.218 | 0.038 | **↑** | 3.541** | 0.026 | 0.430 | 0.034 | **↑** | 0.480 | 0.006 | 0.051 | 0.020 | **↑** | 0.509 | 0.003 | 0.054 | 0.003 | **↑** |
| Hatiya | -1.399 | -0.011 | -0.149 | -0.013 | **↓** | 0.538 | 0.008 | 0.057 | 0.005 | **↑** | 1.712 | 0.019 | 0.259 | 0.020 | **↑** | -2.524* | -0.042 | -0.323 | -0.043 | **↓** | -2.925 ** | -0.044 | -0.415 | -0.057 | **↓** |
| Ishurdi | 3.727** | 0.031 | 0.509 | 0.037 | **↑** | 3.473** | 0.046 | 0.458 | 0.056 | **↑** | 4.216** | 0.041 | 0.592 | 0.044 | **↑** | 0.392 | 0.006 | 0.041 | 0.028 | **↑** | 1.125 | 0.007 | 0.117 | 0.016 | **↑** |
| Jessore | 3.042** | 0.022 | 0.364 | 0.023 | **↑** | 3.238** | 0.040 | 0.315 | 0.039 | **↑** | 4.854** | 0.032 | 0.502 | 0.041 | **↑** | 0.333 | 0.005 | 0.035 | 0.017 | **↑** | -0.783 | -0.010 | -0.082 | -0.011 | **↓** |
| Khepupara | 0.831 | 0.005 | 0.122 | 0.000 | **↑** | 1.409 | 0.016 | 0.146 | -0.004 | **↓** | 3.894** | 0.031 | 0.403 | 0.034 | **↑** | -1.987* | -0.025 | -0.206 | -0.011 | **↓** | -2.084* | -0.031 | -0.346 | -0.033 | **↓** |
| Khulna | 2.690** | 0.026 | 0.392 | 0.031 | **↑** | 3.492** | 0.044 | 0.424 | 0.041 | **↑** | 3.327** | 0.027 | 0.344 | 0.027 | **↑** | -1.047 | 0.000 | -0.010 | 0.014 | **↑** | 2.427* | 0.031 | 0.252 | 0.038 | **↑** |
| M. Court | 3.688** | 0.025 | 0.443 | 0.028 | **↑** | 3.670** | 0.041 | 0.380 | 0.045 | **↑** | 2.504* | 0.018 | 0.318 | 0.020 | **↑** | 1.194 | 0.016 | 0.124 | 0.023 | **↑** | 2.701** | 0.024 | 0.280 | 0.027 | **↑** |
| Mymensingh | 3.395** | 0.017 | 0.352 | 0.021 | **↑** | 3.189** | 0.033 | 0.330 | 0.039 | **↑** | 4.629** | 0.025 | 0.479 | 0.033 | **↑** | -0.176 | -0.004 | -0.019 | -0.003 | **↓** | 0.440 | 0.003 | 0.046 | 0.001 | **↑** |
| Patuakhali | 1.702 | 0.008 | 0.177 | 0.005 | **↑** | 1.448 | 0.017 | 0.151 | -0.002 | **↓** | 4.493** | 0.037 | 0.465 | 0.039 | **↑** | -0.832 | -0.017 | -0.087 | -0.003 | **↓** | -1.742 | -0.021 | -0.181 | -0.027 | **↓** |
| Rajshahi | 0.558 | 0.010 | 0.133 | 0.024 | **↑** | 2.563* | 0.031 | 0.289 | 0.040 | **↑** | 4.431** | 0.040 | 0.514 | 0.048 | **↑** | -1.214 | -0.020 | -0.126 | 0.007 | **↑** | -2.674 ** | -0.029 | -0.277 | -0.013 | **↓** |
| Rangamati | -2.925** | -0.029 | -0.375 | -0.025 | **↓** | -2.377* | -0.025 | -0.261 | -0.019 | **↓** | 1.859 | 0.013 | 0.193 | 0.023 | **↑** | -3.003** | -0.067 | -0.417 | -0.052 | **↓** | -4.431 ** | -0.080 | -0.417 | -0.076 | **↓** |
| Rangpur | 3.473** | 0.031 | 0.513 | 0.034 | **↑** | 2.201* | 0.048 | 0.388 | 0.057 | **↑** | 3.375** | 0.030 | 0.416 | 0.030 | **↑** | 3.758** | 0.048 | 0.389 | 0.047 | **↑** | 0.499 | 0.004 | 0.053 | 0.008 | **↑** |
| Sandwip | -1.418 | -0.011 | -0.127 | -0.018 | **↓** | 0.578 | 0.006 | 0.061 | -0.004 | **↓** | 0.646 | 0.010 | 0.167 | 0.003 | **↑** | -2.819** | -0.031 | -0.292 | -0.022 | **↓** | -3.668 ** | -0.046 | -0.340 | -0.059 | **↓** |
| Satkhira | 1.497 | 0.012 | 0.222 | 0.010 | **↑** | 1.027 | 0.015 | 0.107 | 0.002 | **↑** | 2.142* | 0.021 | 0.276 | 0.023 | **↑** | 0.754 | 0.010 | 0.079 | 0.019 | **↑** | 0.039 | 0.000 | 0.005 | -0.006 | **↓** |
| Srimangal | 0.382 | 0.005 | 0.119 | 0.002 | **↑** | -0.068 | 0.002 | 0.031 | 0.003 | **↑** | 3.503** | 0.023 | 0.363 | 0.009 | **↑** | -1.752 | -0.024 | -0.182 | -0.004 | **↓** | 0.294 | 0.001 | 0.031 | -0.003 | **↓** |
| Sylhet | 4.833** | 0.035 | 0.574 | 0.037 | **↑** | 4.424** | 0.042 | 0.458 | 0.043 | **↑** | 4.080** | 0.029 | 0.492 | 0.036 | **↑** | 2.554* | 0.023 | 0.265 | 0.025 | **↑** | 3.473 ** | 0.046 | 0.360 | 0.041 | **↑** |
| Teknaf | 1.614 | 0.018 | 0.269 | 0.019 | **↑** | 2.506* | 0.028 | 0.260 | 0.029 | **↑** | 1.908 | 0.029 | 0.439 | 0.032 | **↑** | 1.459 | 0.016 | 0.152 | 0.030 | **↑** | -1.830 | -0.020 | -0.190 | -0.020 | **↓** |

*N.B.: * Trends at the 95% confidence level(Z=1.96); ** Trends at the 99% confidence level(Z=2.58).*

**Figure S1: Research Workflow Diagram**


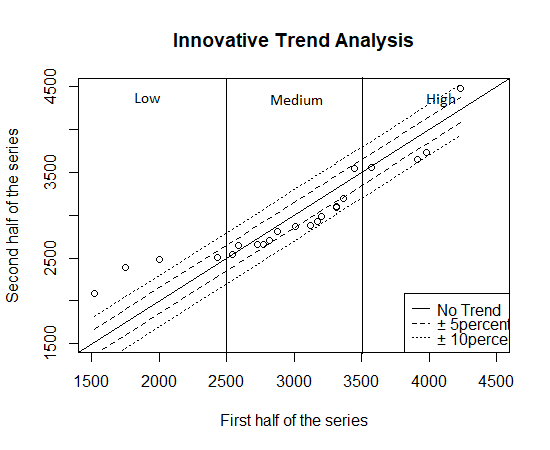


**Figure S2: Example of the innovative trend analysis (ITA) method**

| 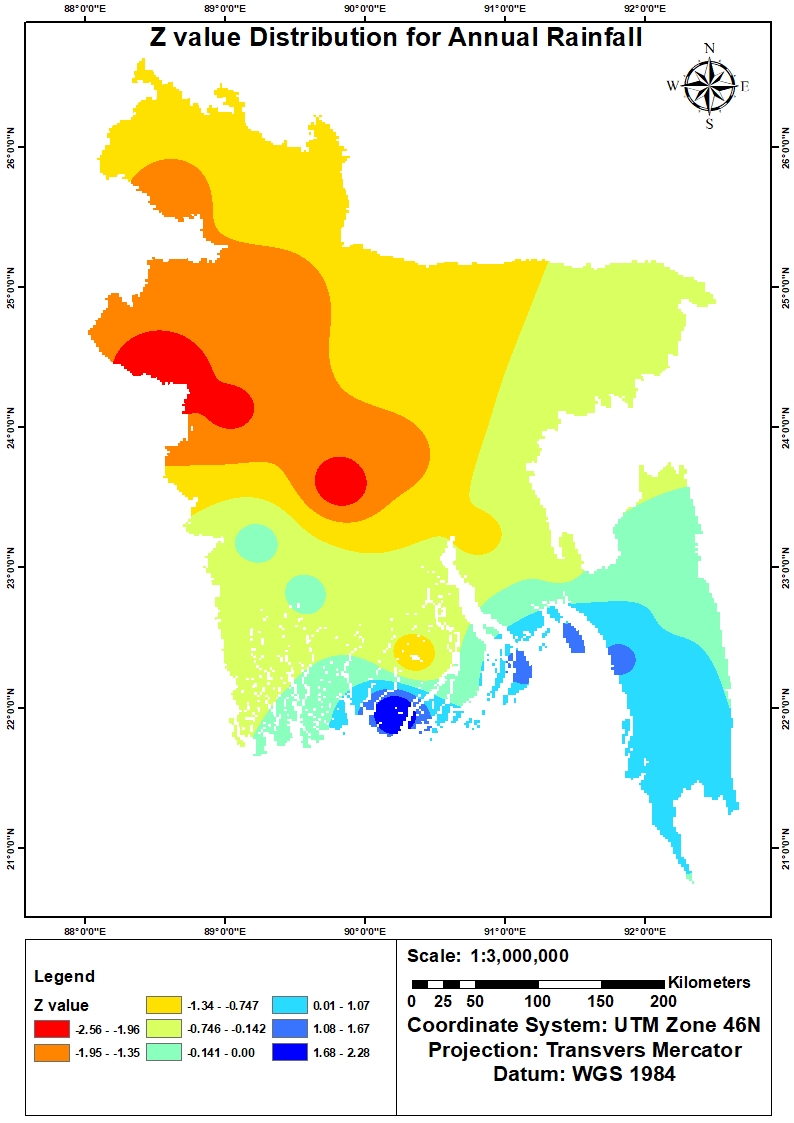 | 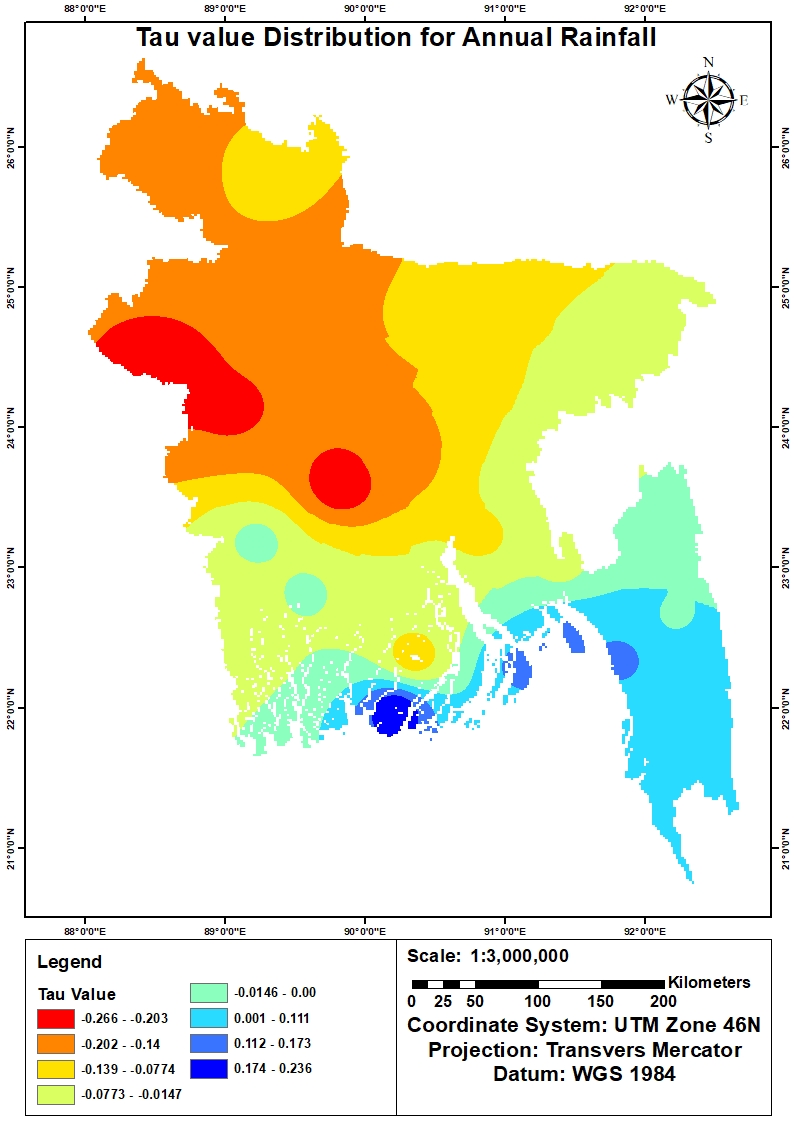 | 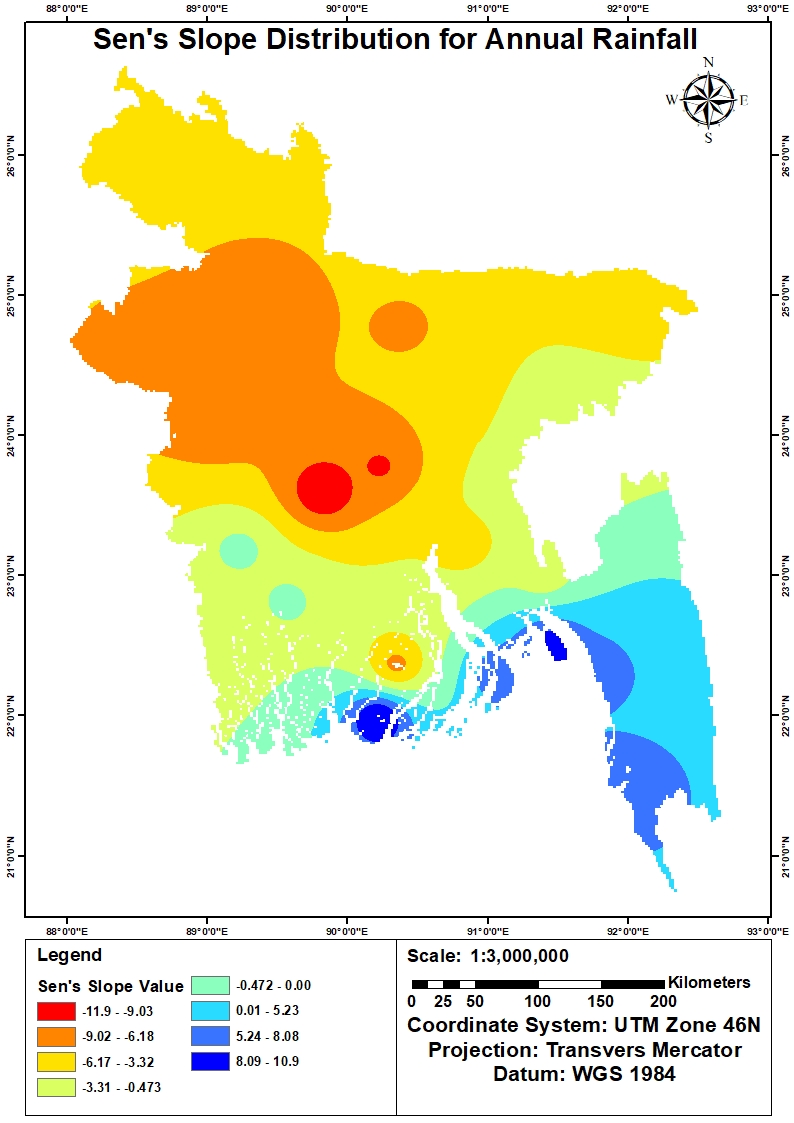 | 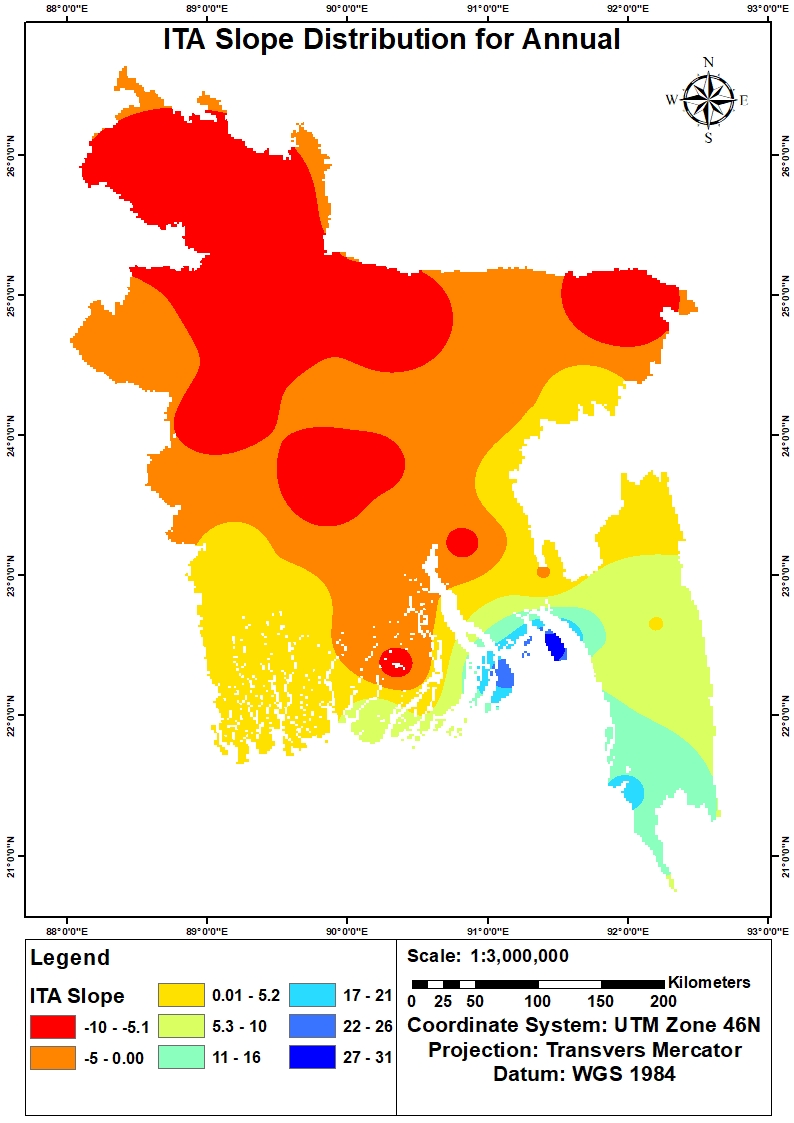 |
| --- | --- | --- | --- |
| **Annual** | | | |
| 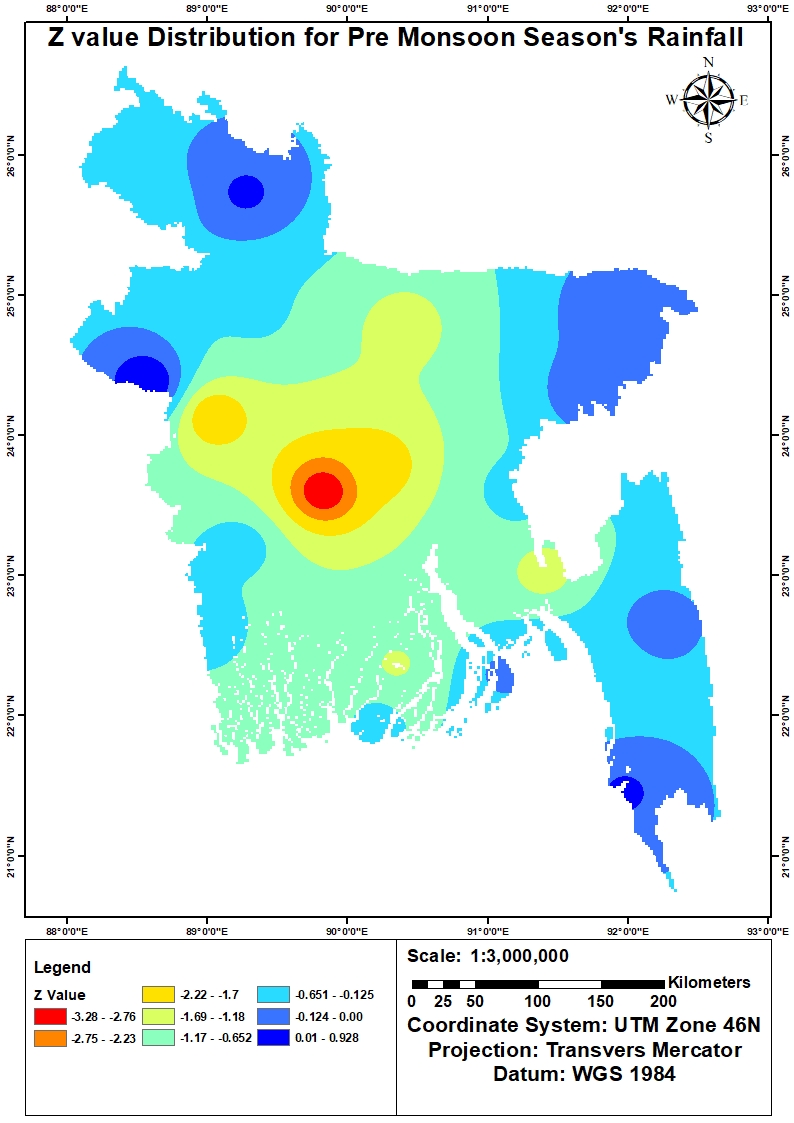 | 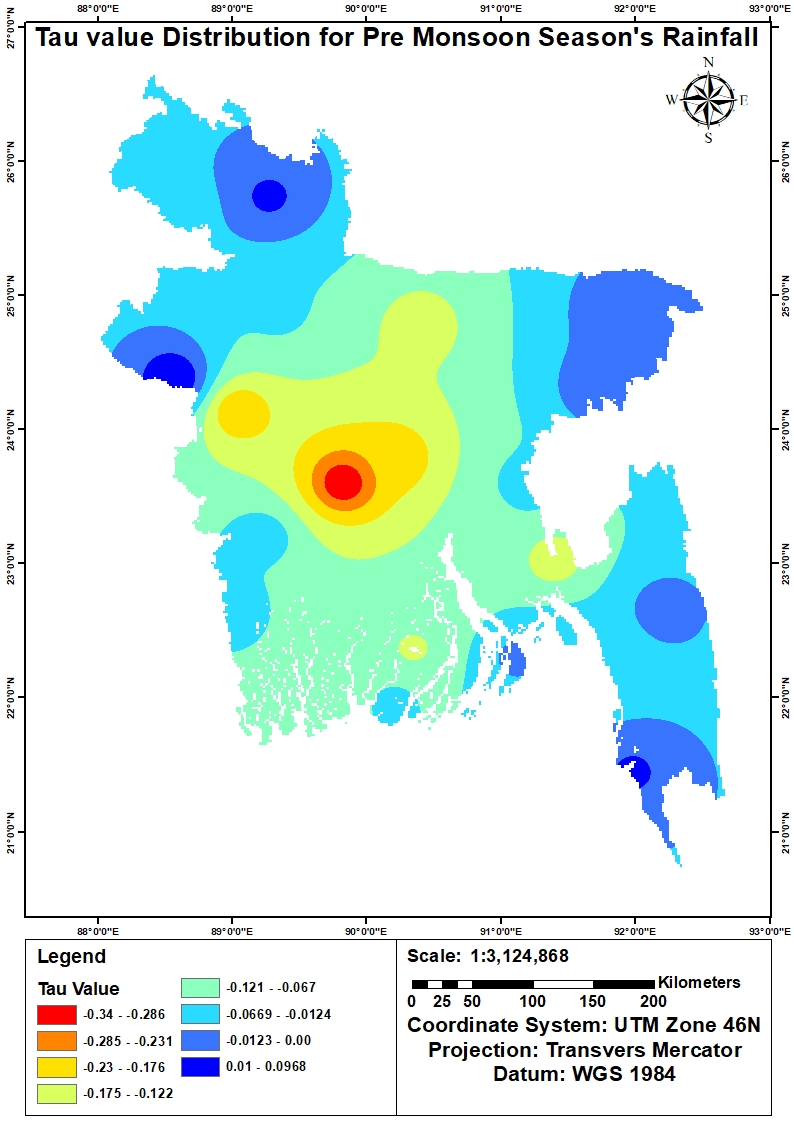 | 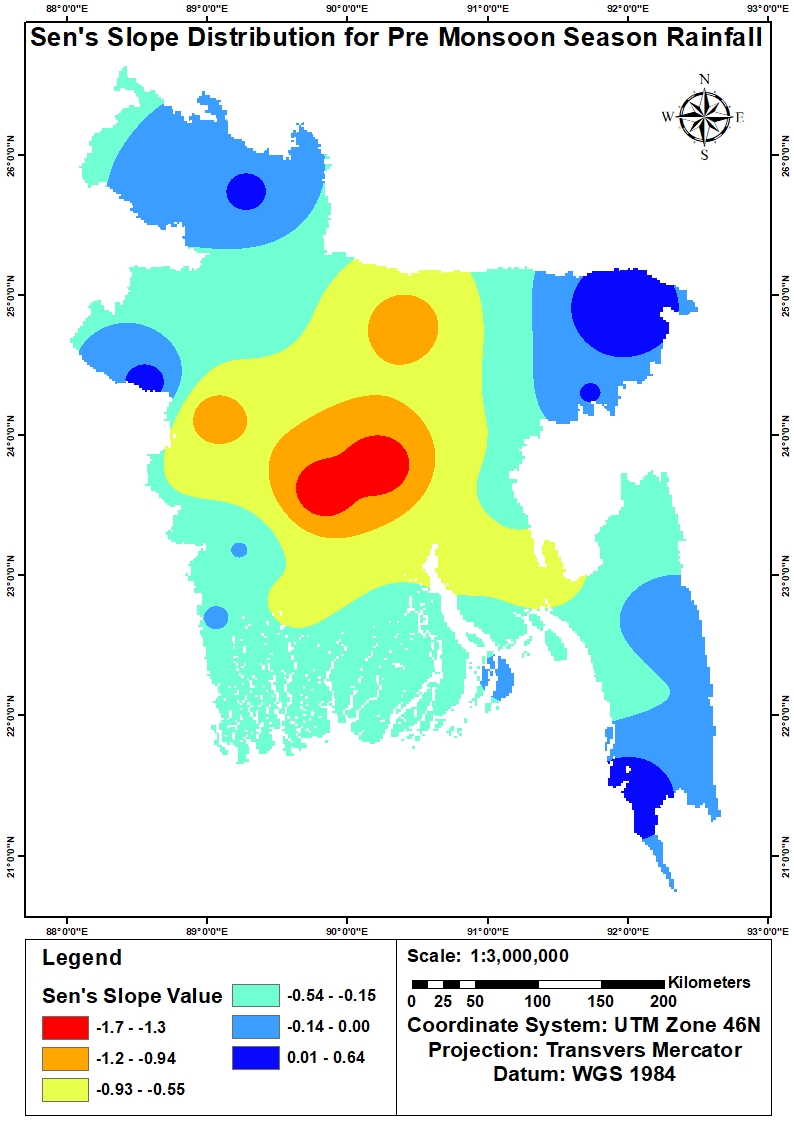 | 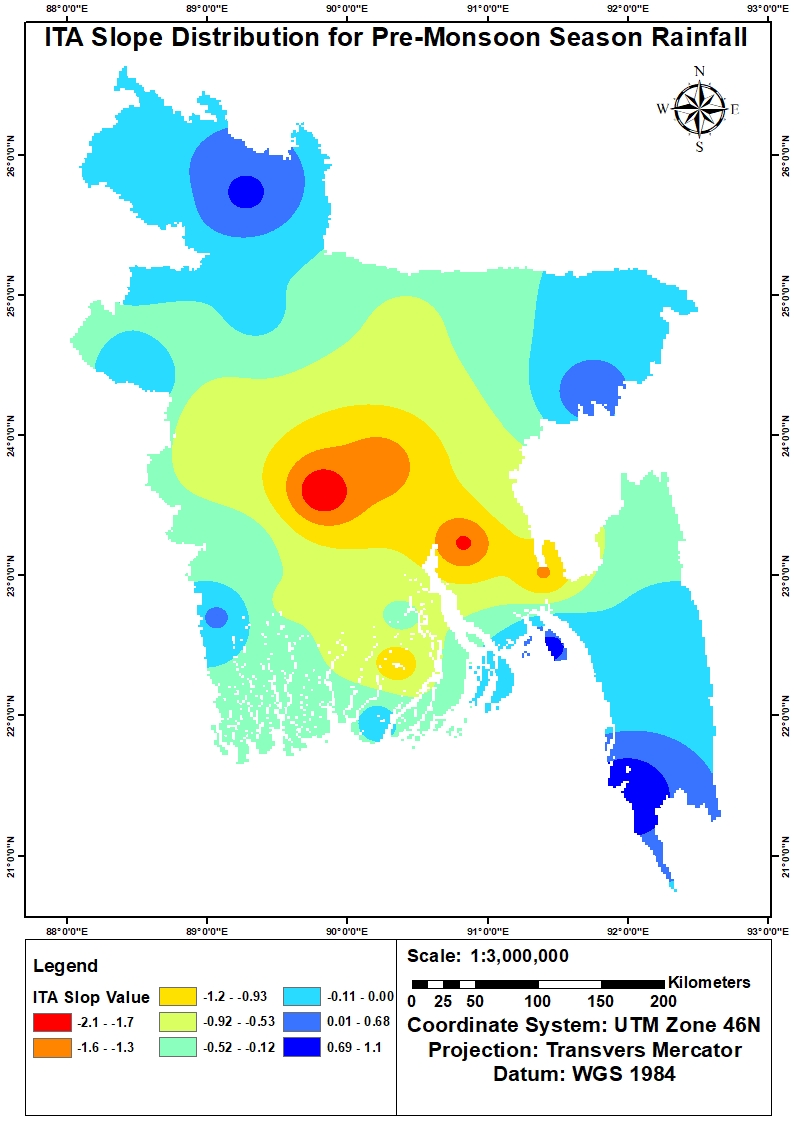 |
| **Pre-monsoon** | | | |
| 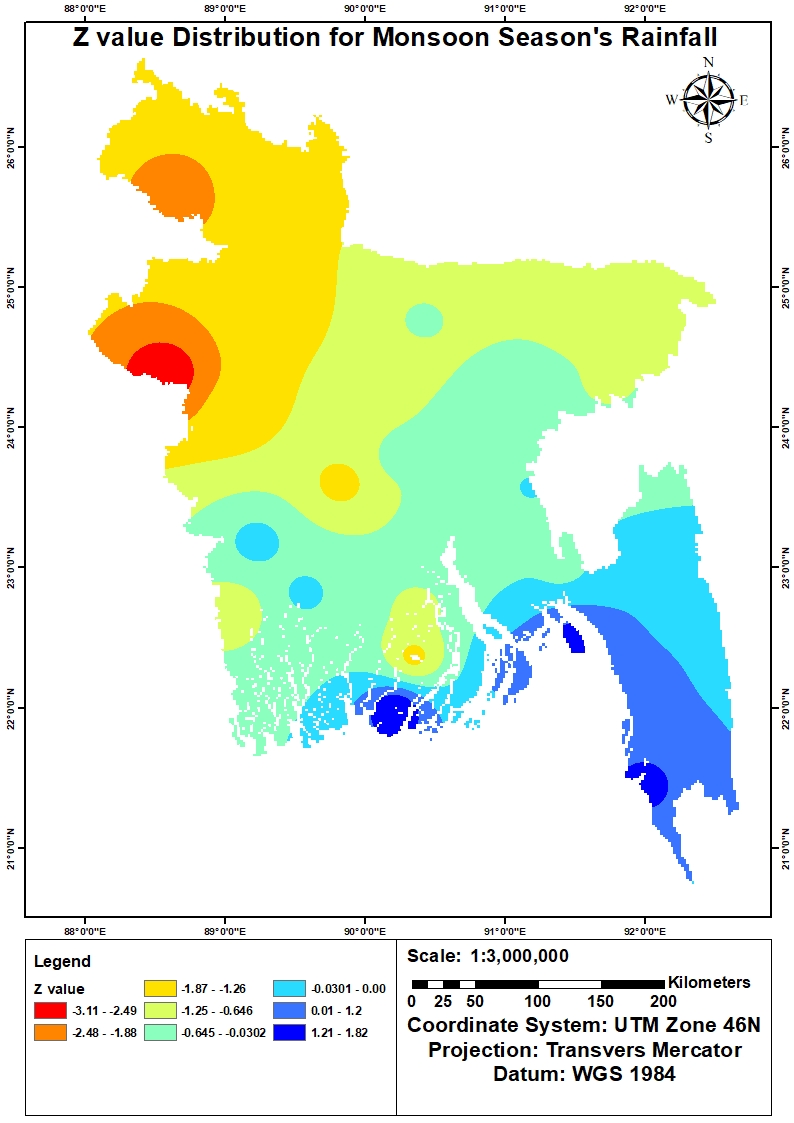 | 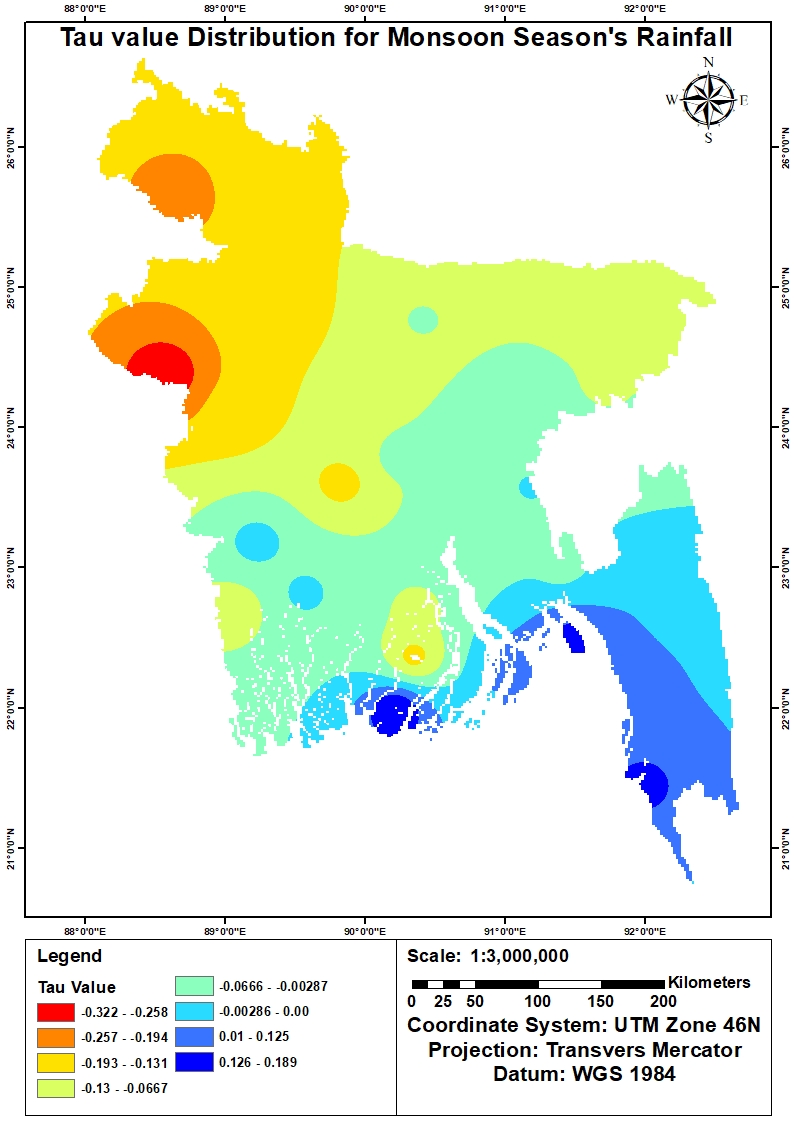 | 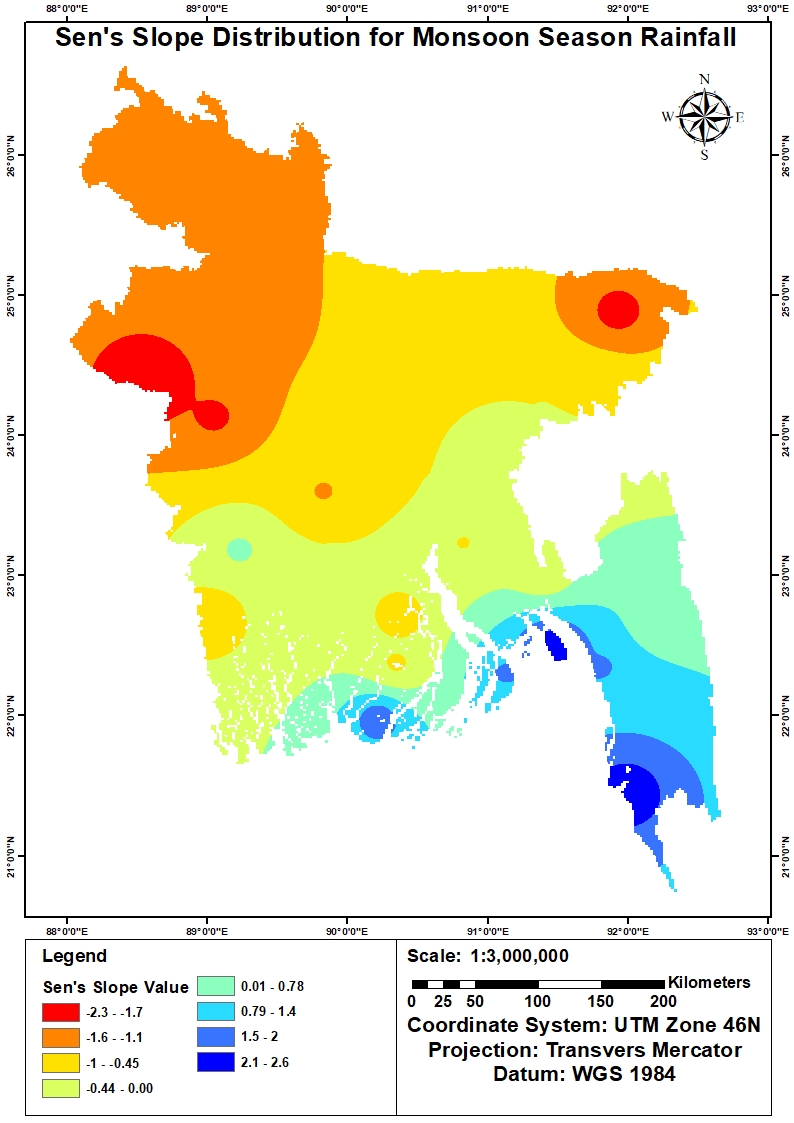 | 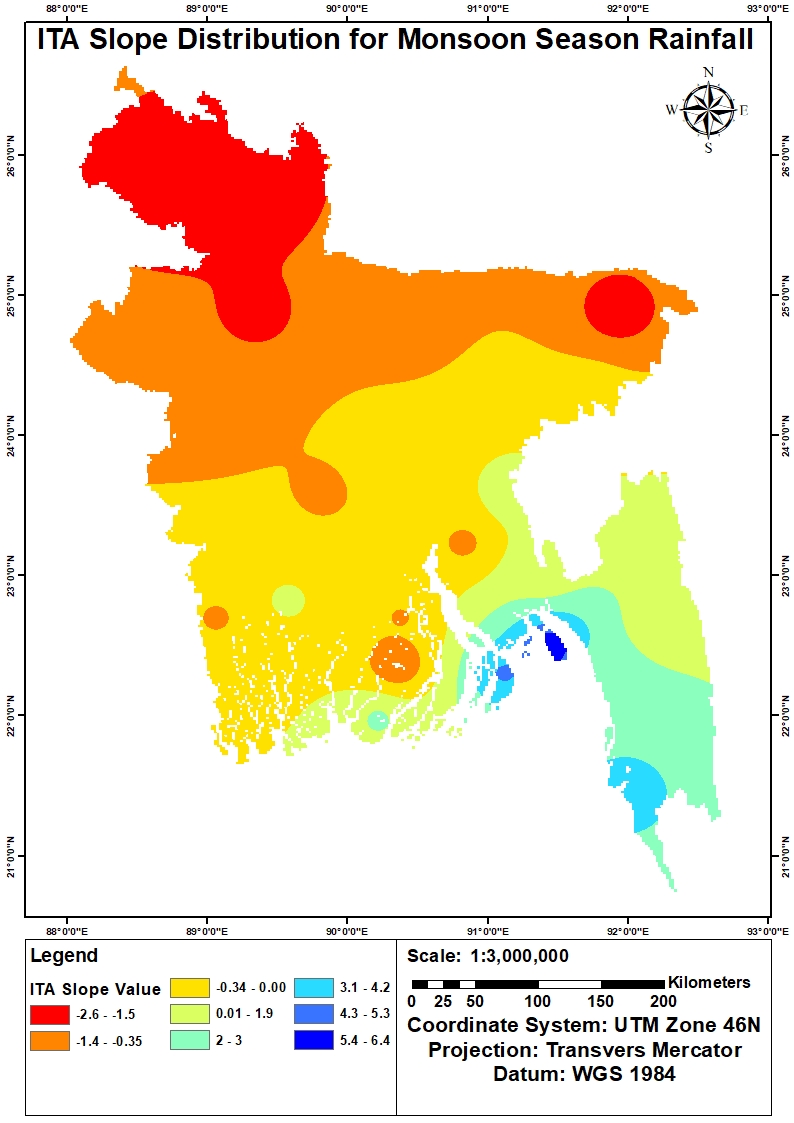 |
| **Monsoon** | | | |
| 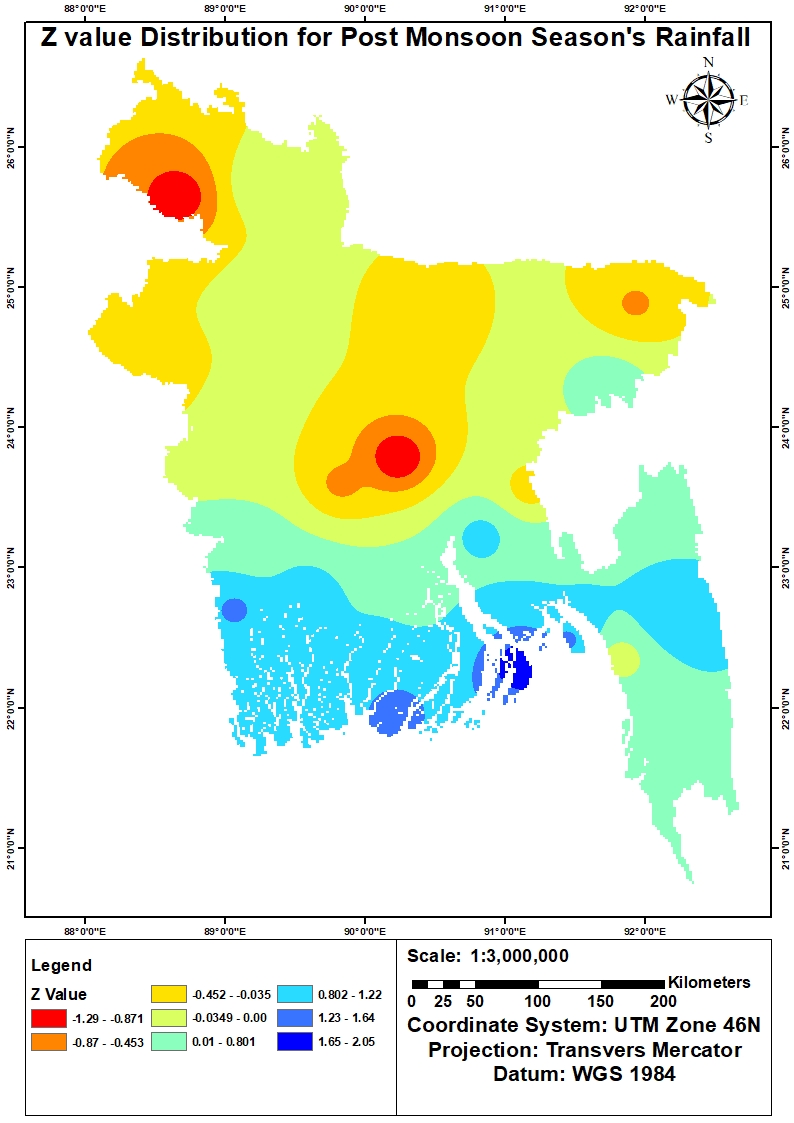 | 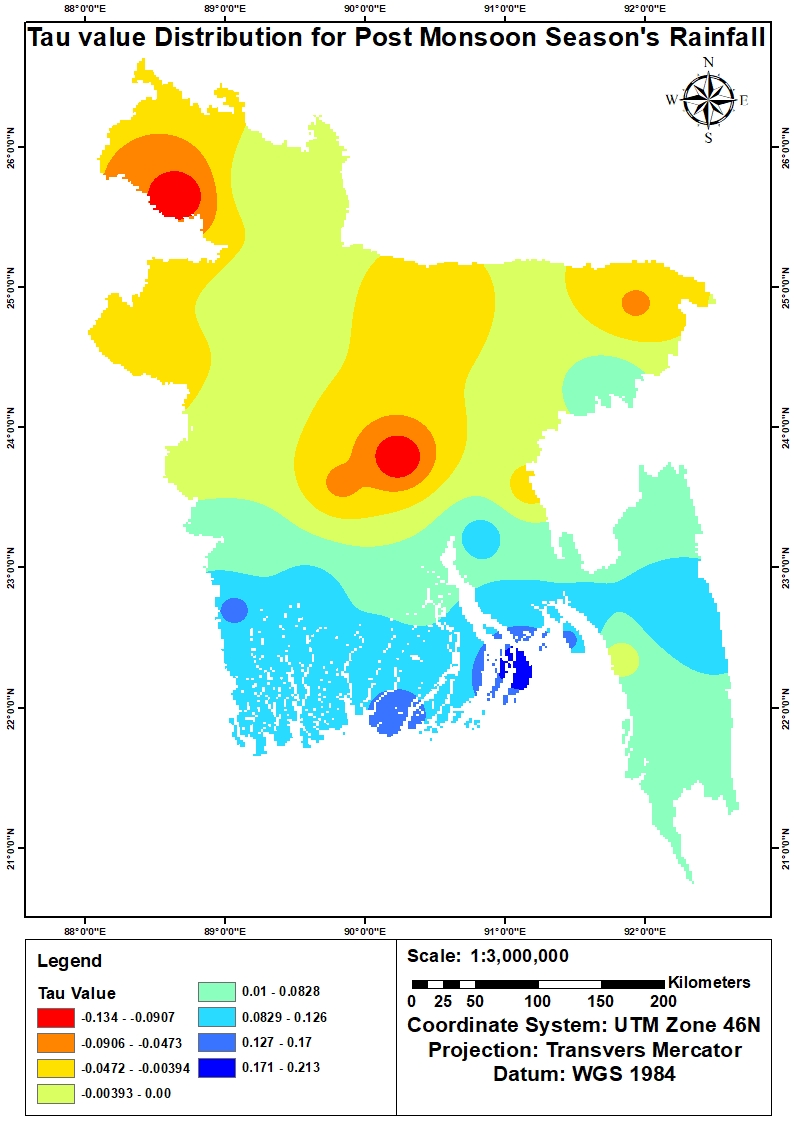 | 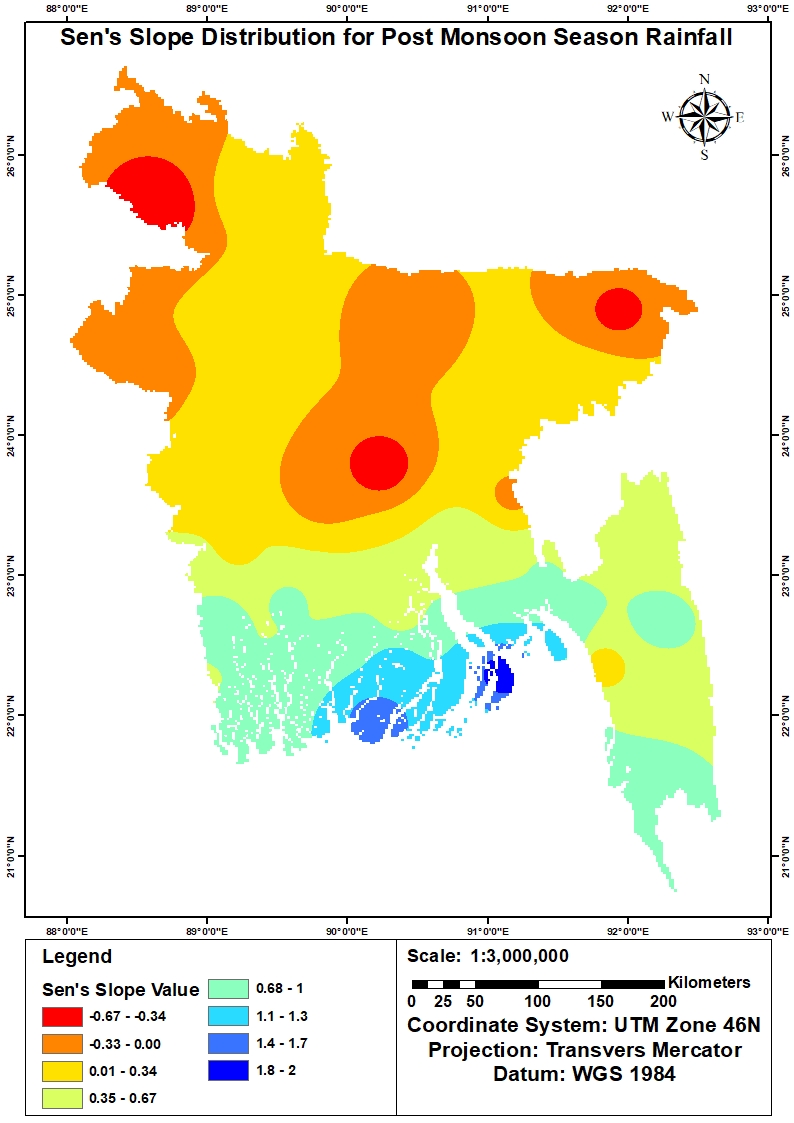 | 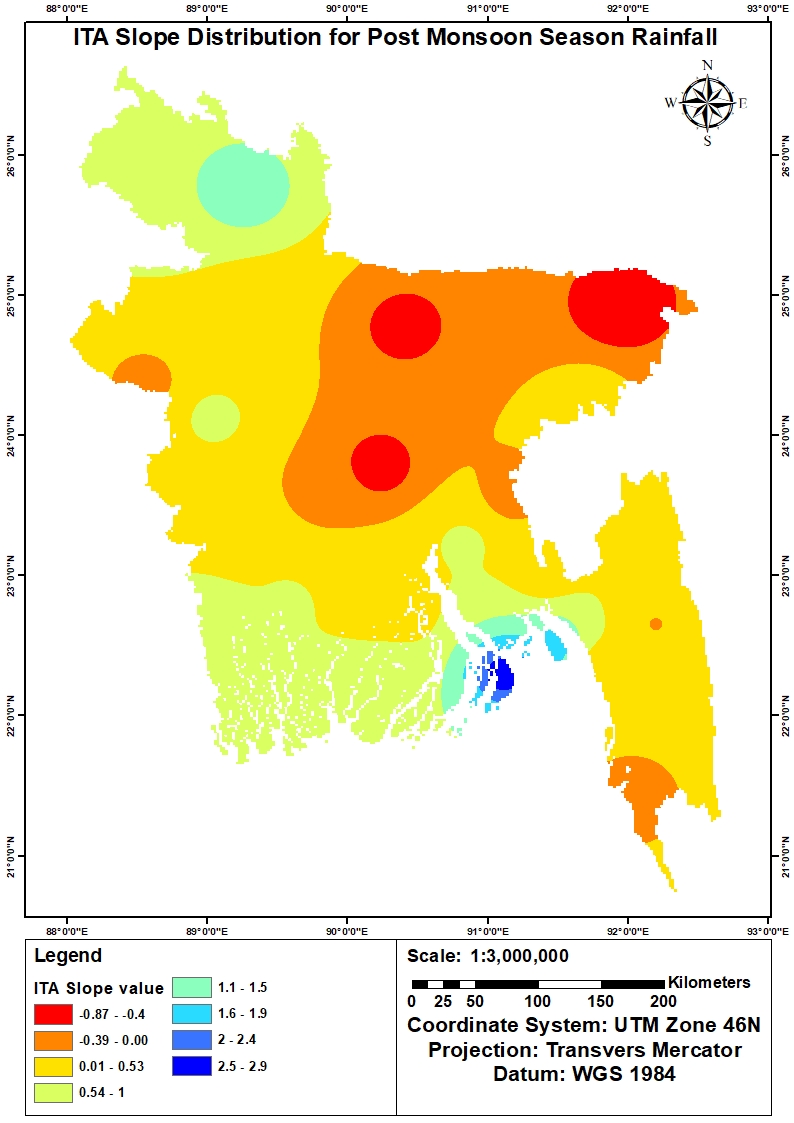 |
| **Post-monsoon** | | | |
| 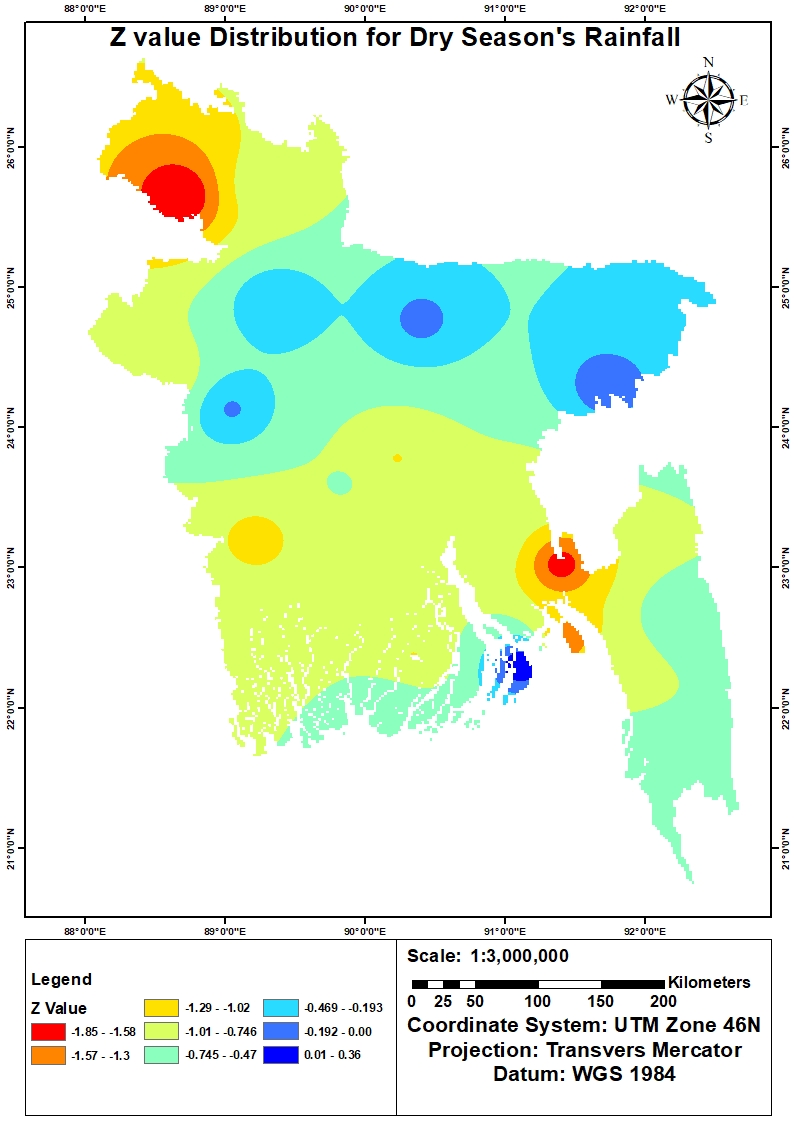 | 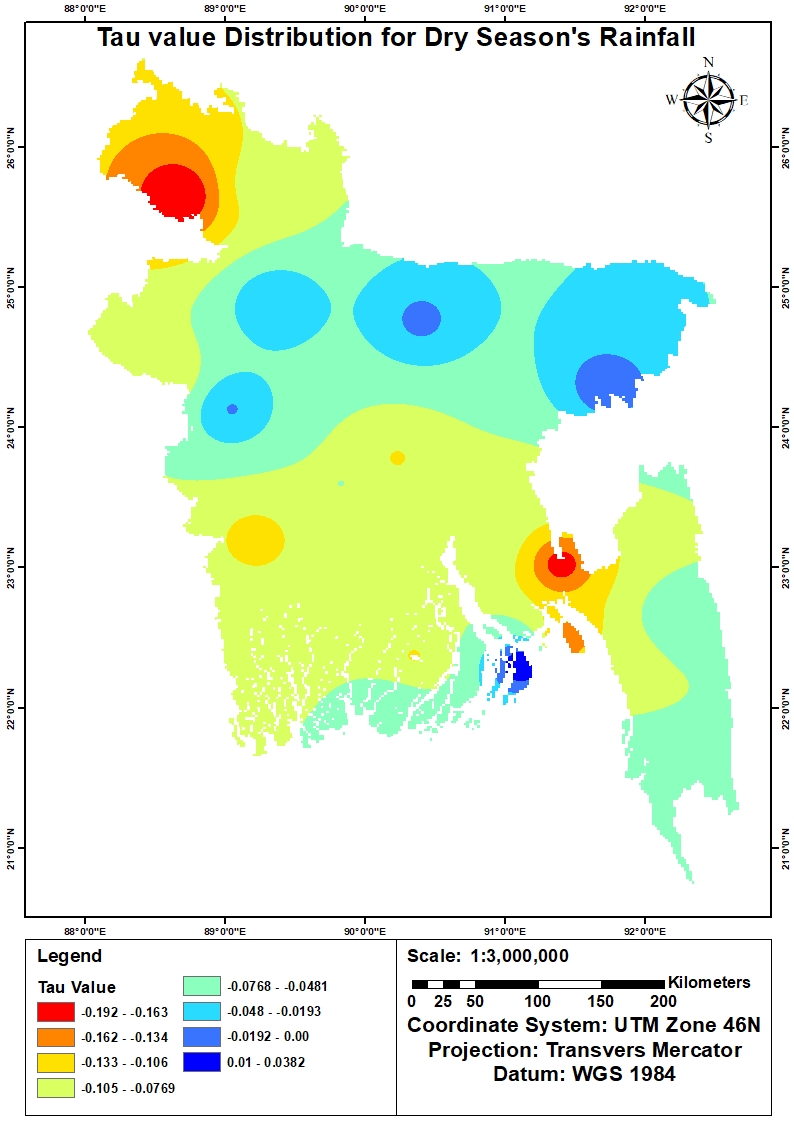 | 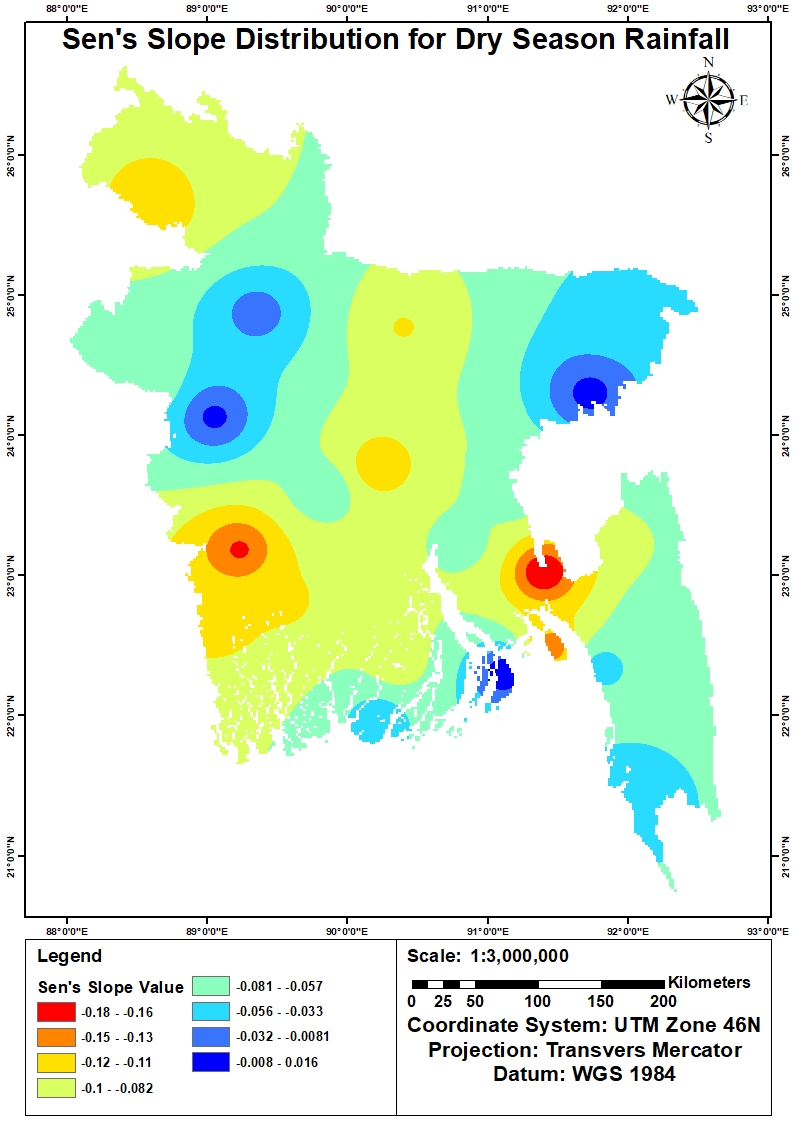 | 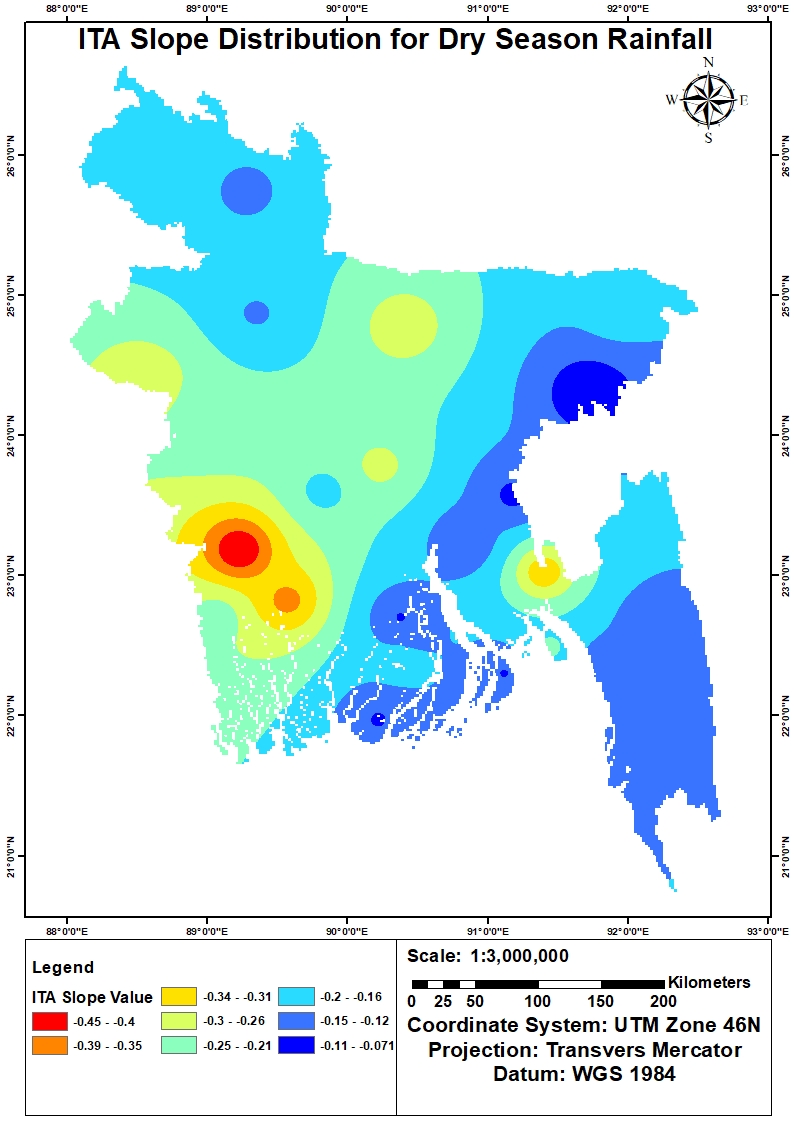 |
| **Dry** | | | |

**Figure S3: Spatial distribution of (a) Annual, (b) Pre-monsoon, (c) Monsoon, (d) Post-monsoon and (e) Dry annual rainfall trend using MK (1st column), mMK (2nd column), ITA (3rd column) and Sen’s slope (4th column) analysis**

| 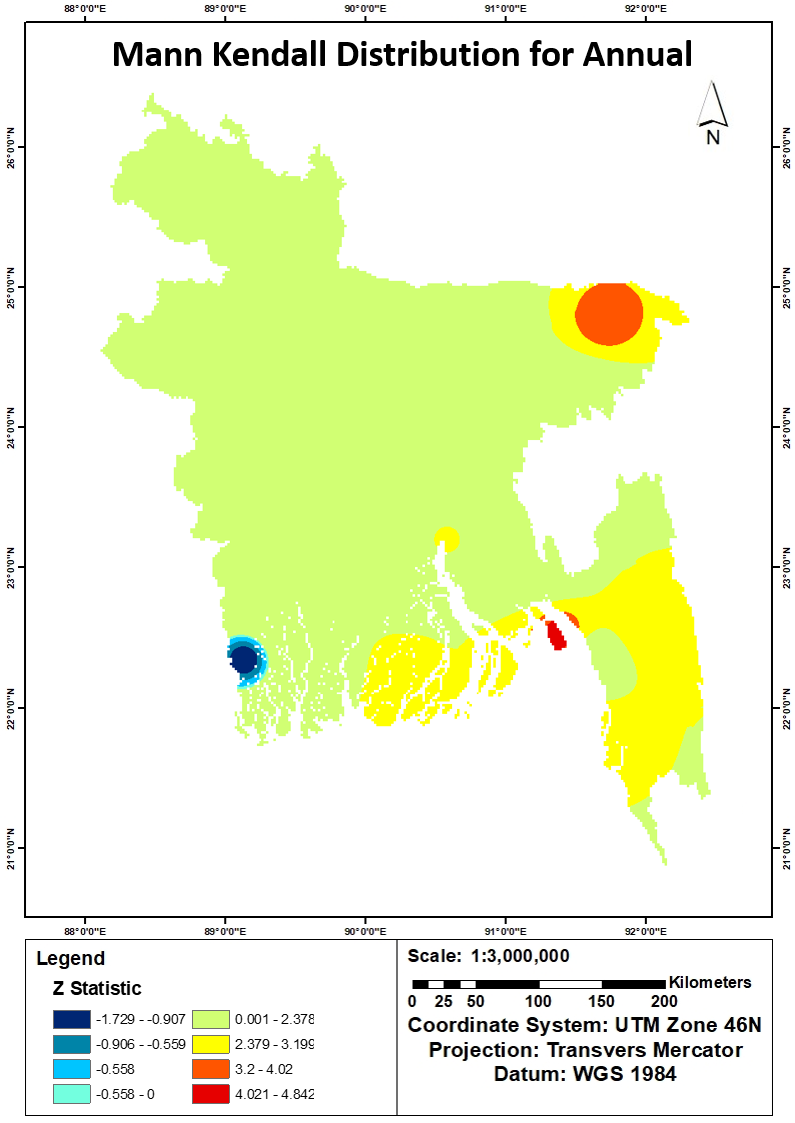 | 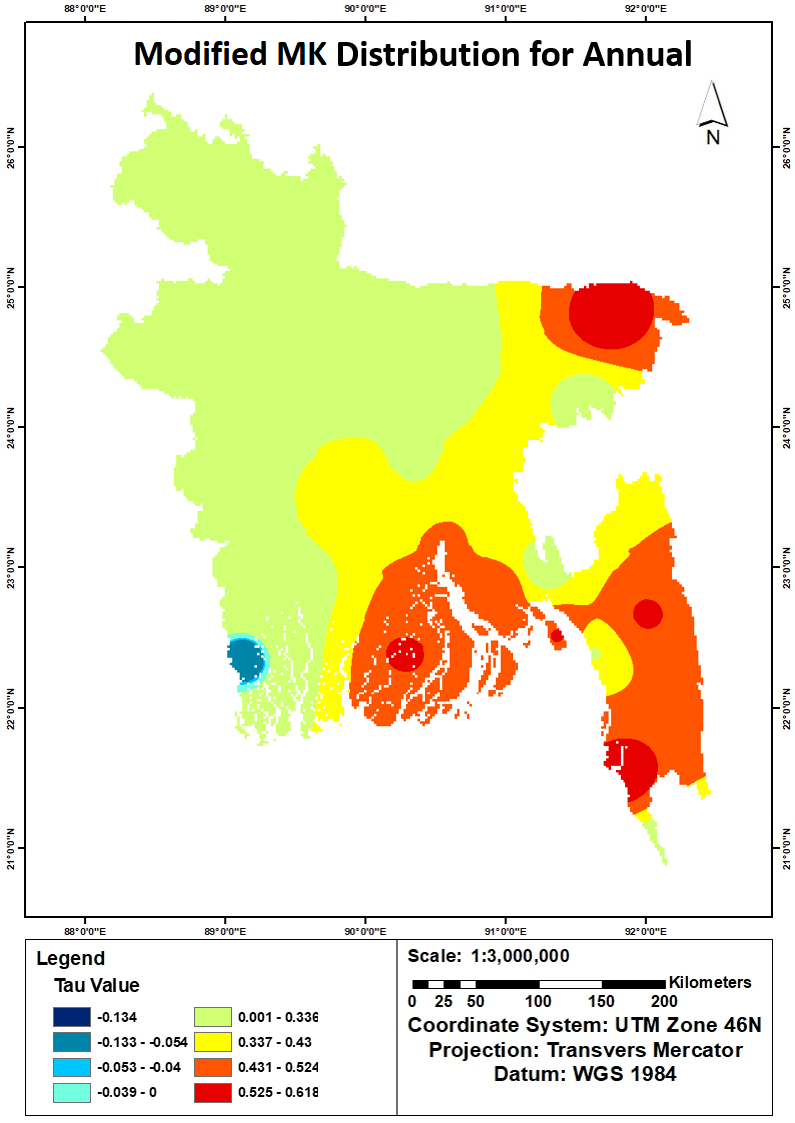 | 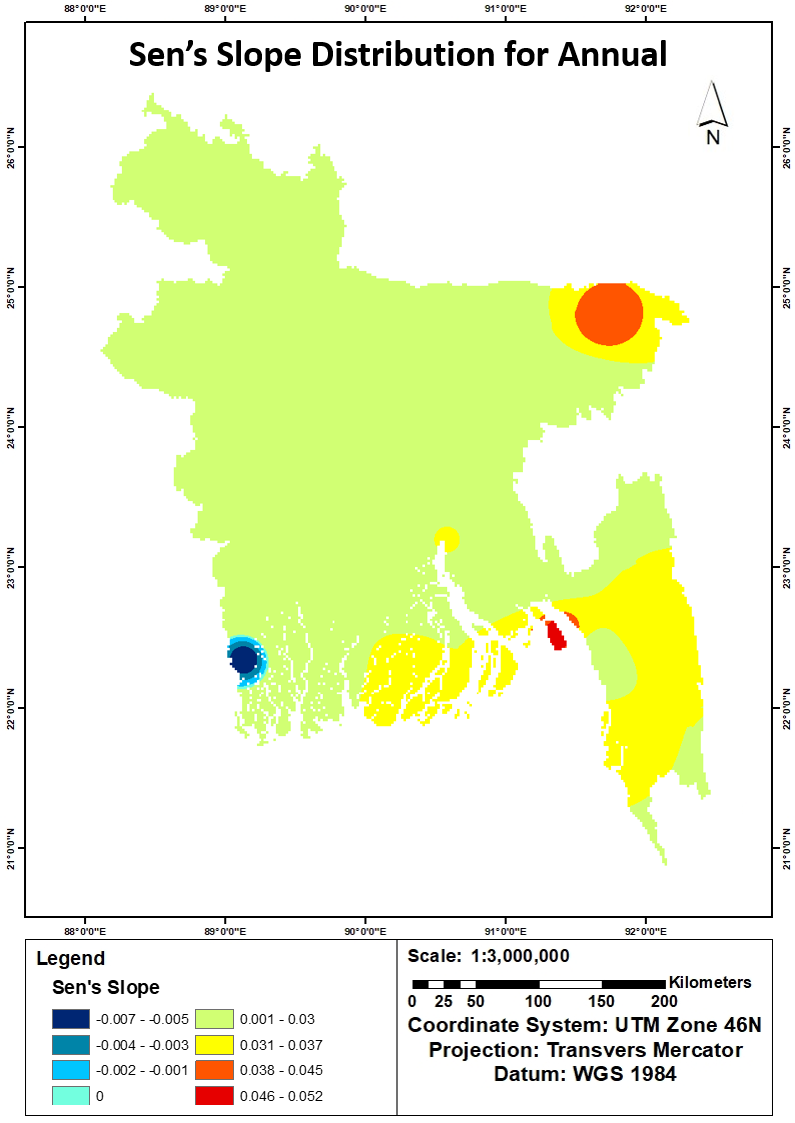 | 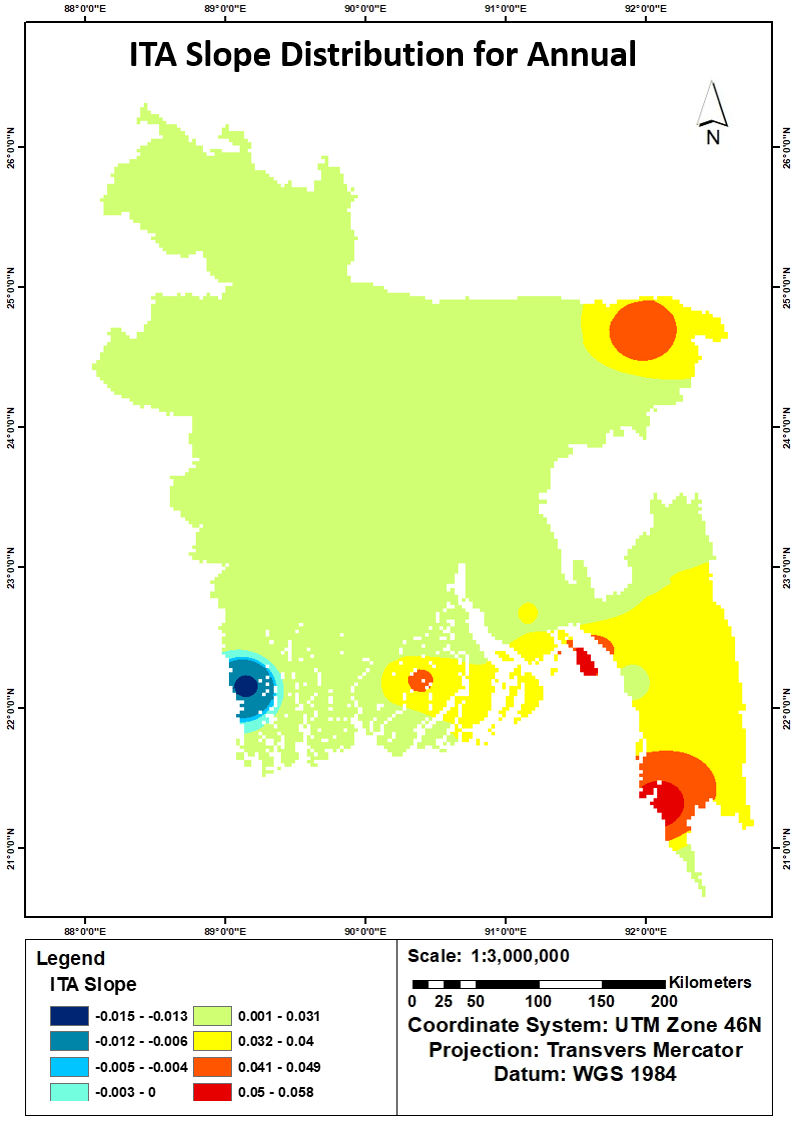 |
| --- | --- | --- | --- |
| **Annual** | | | |
| 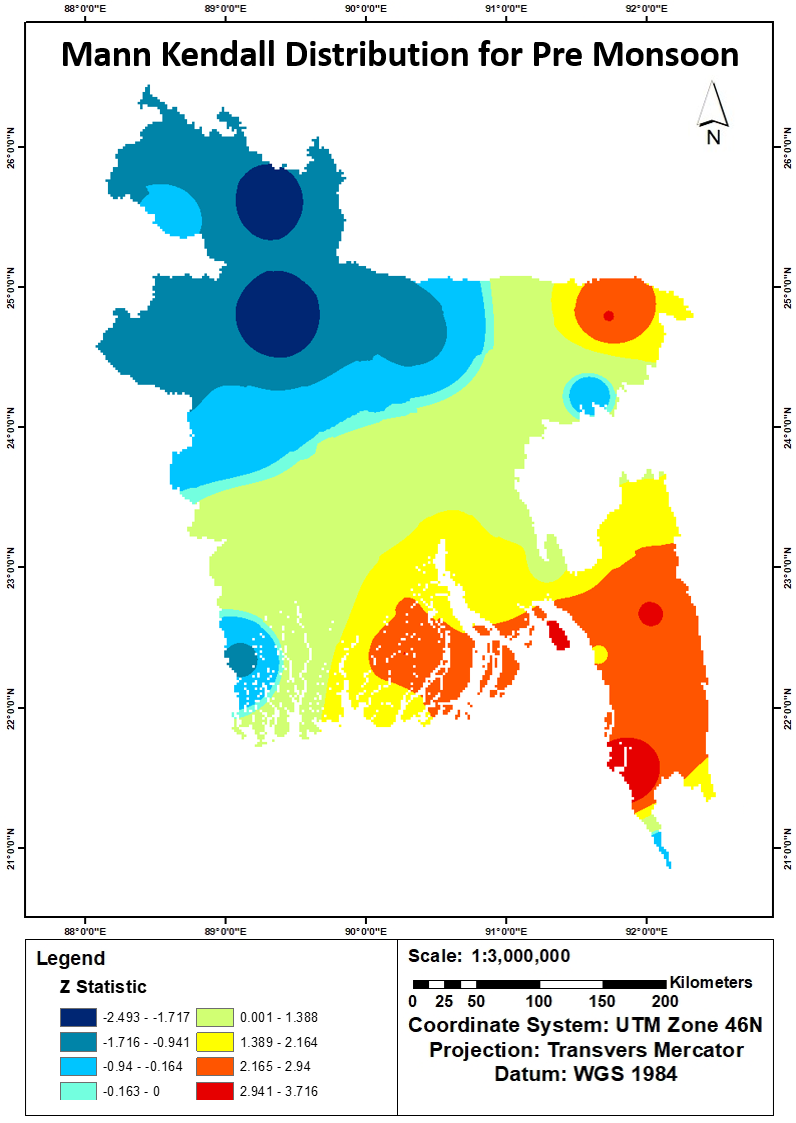 | 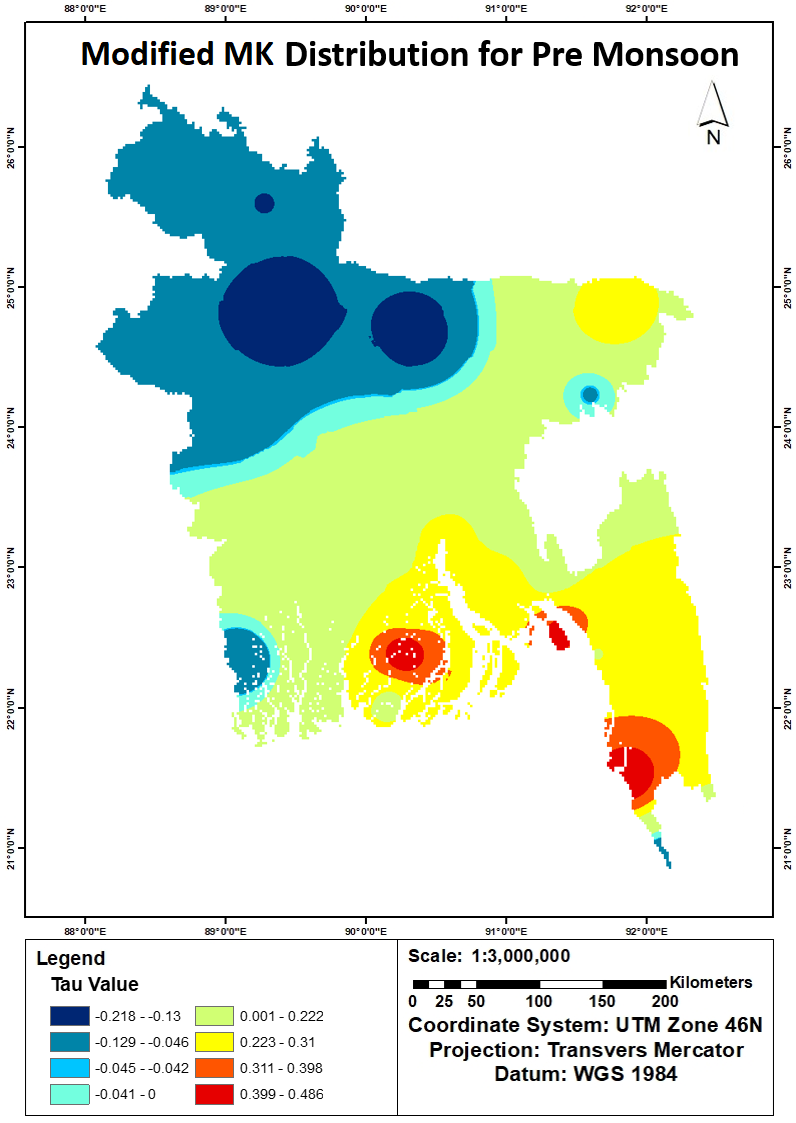 | 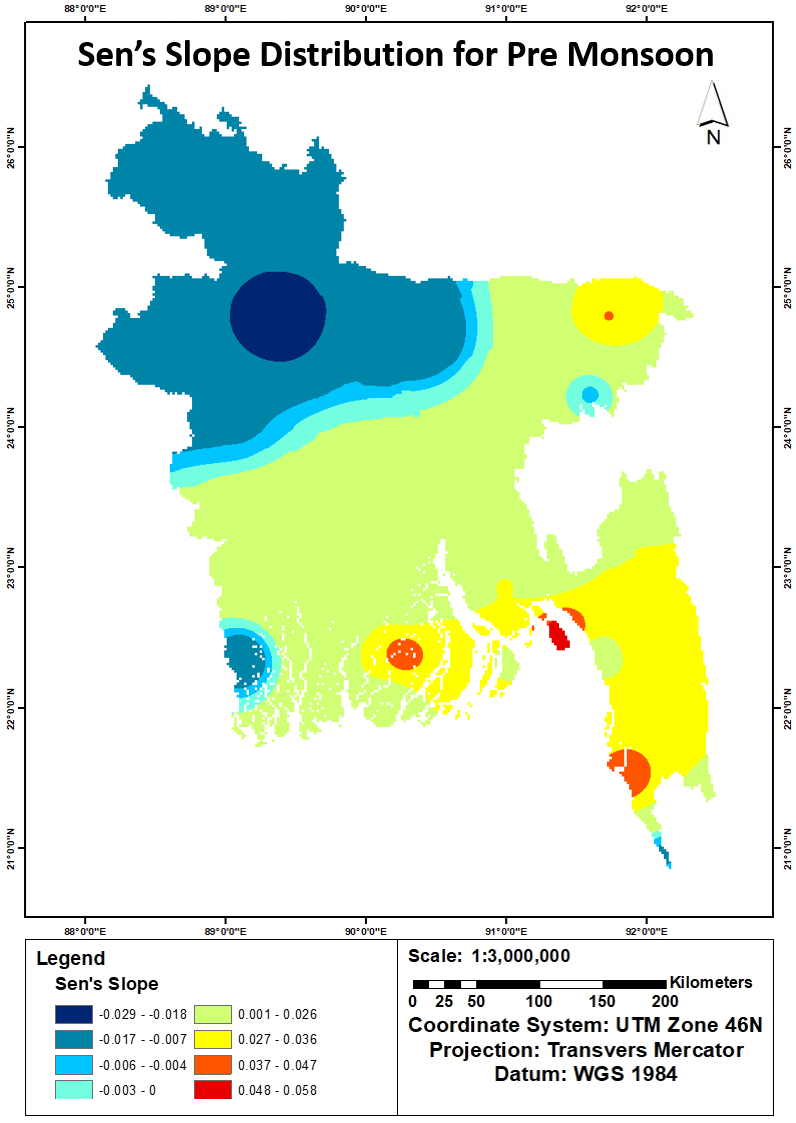 | 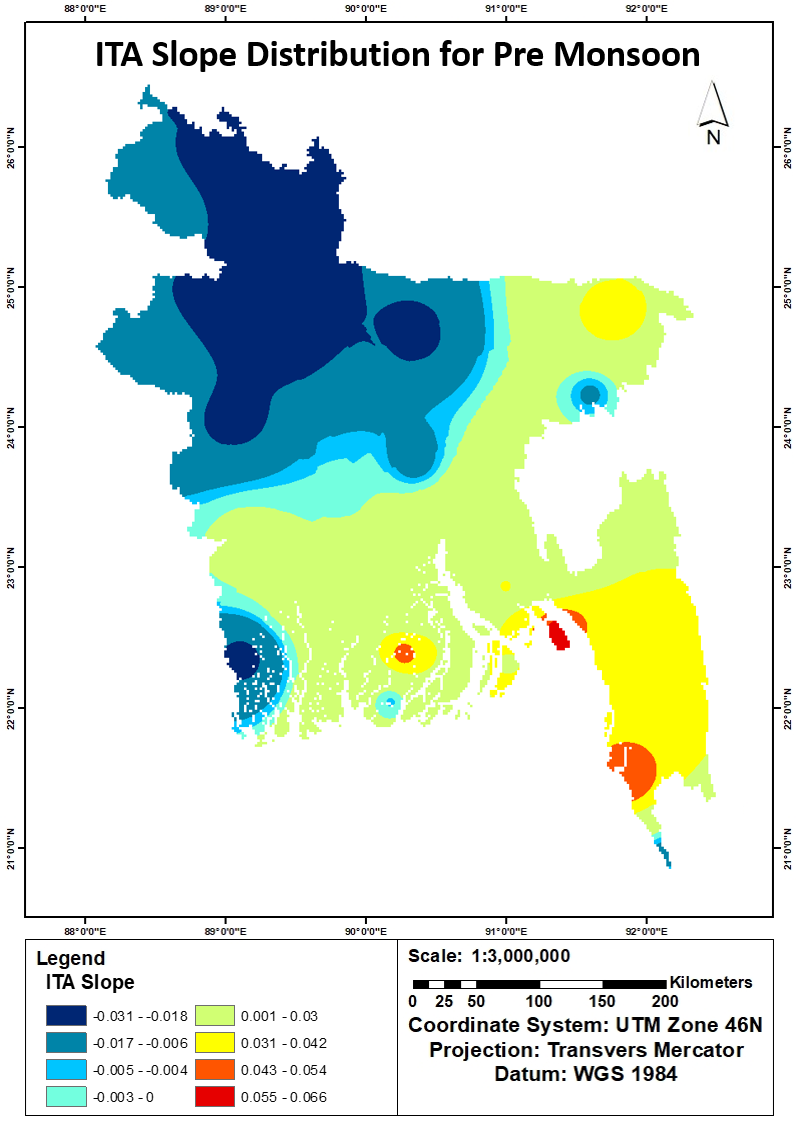 |
| **Pre-monsoon** | | | |
| 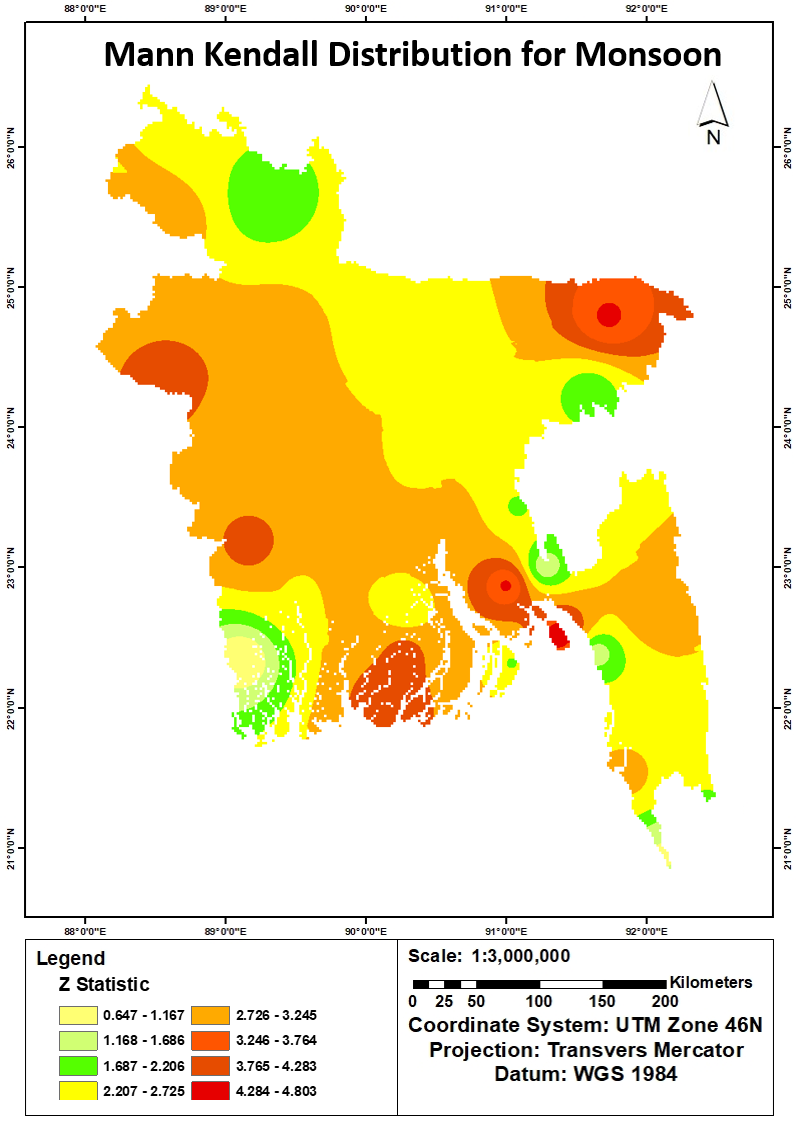 | 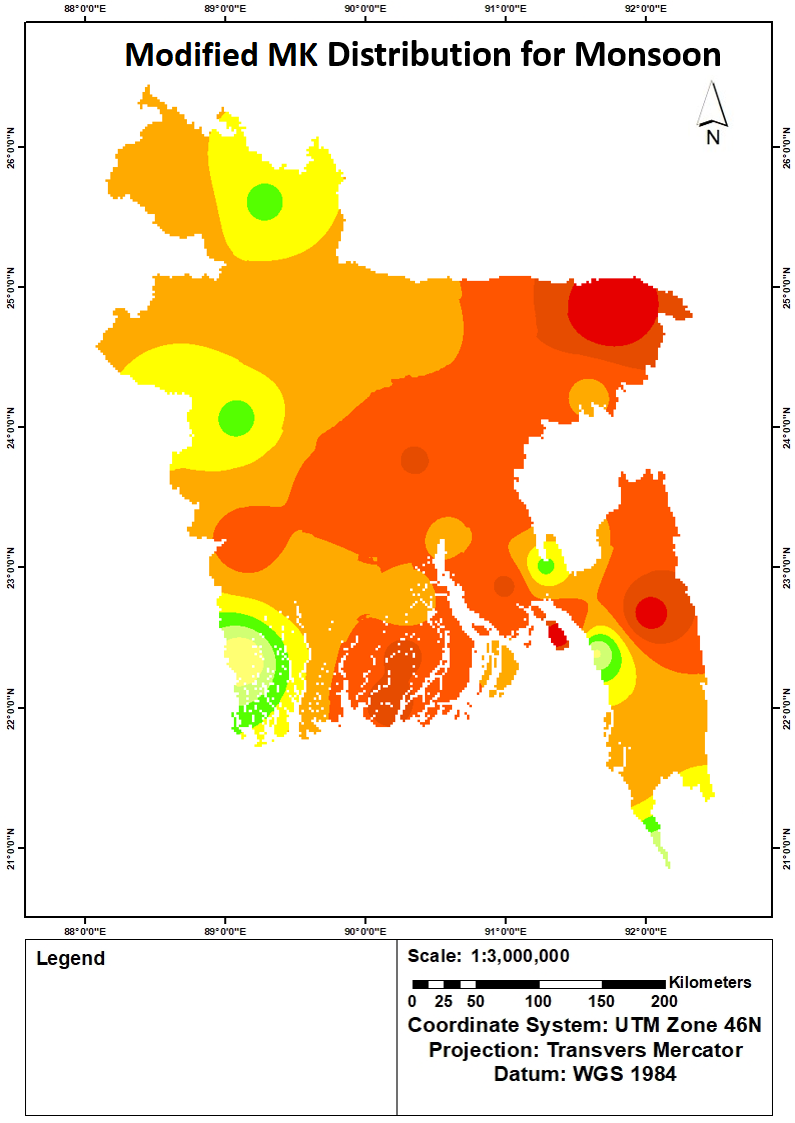 | 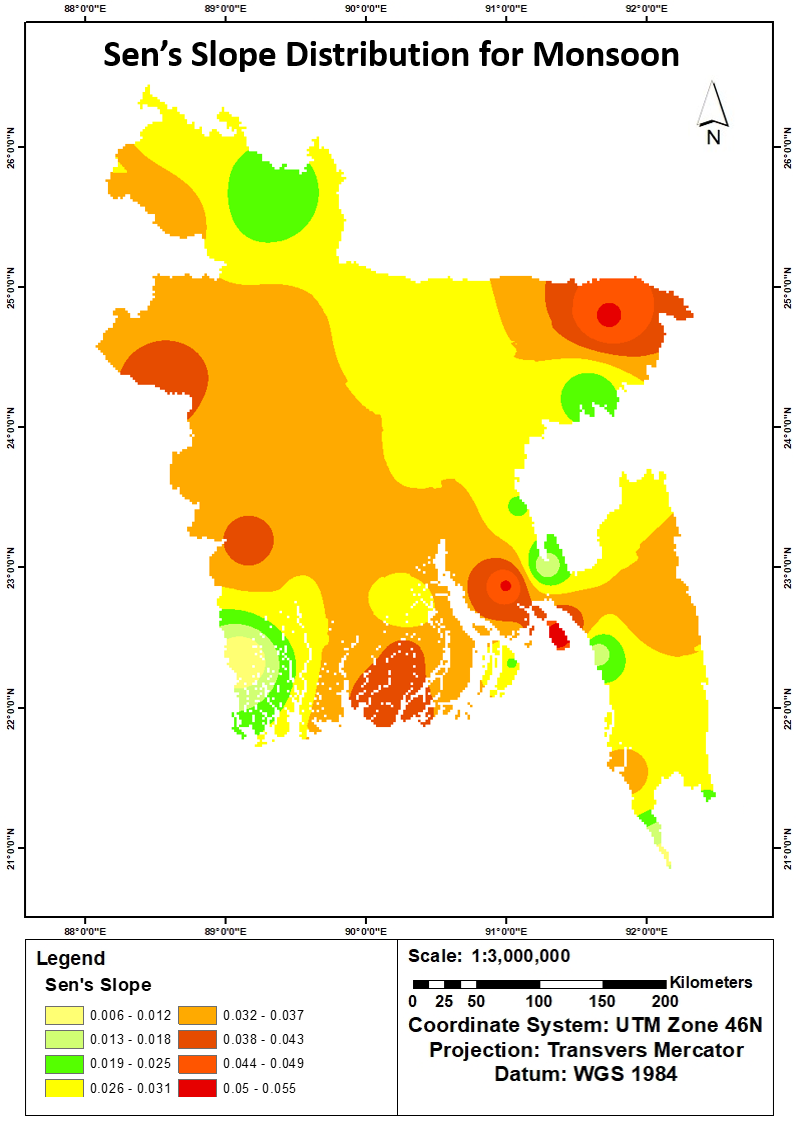 | 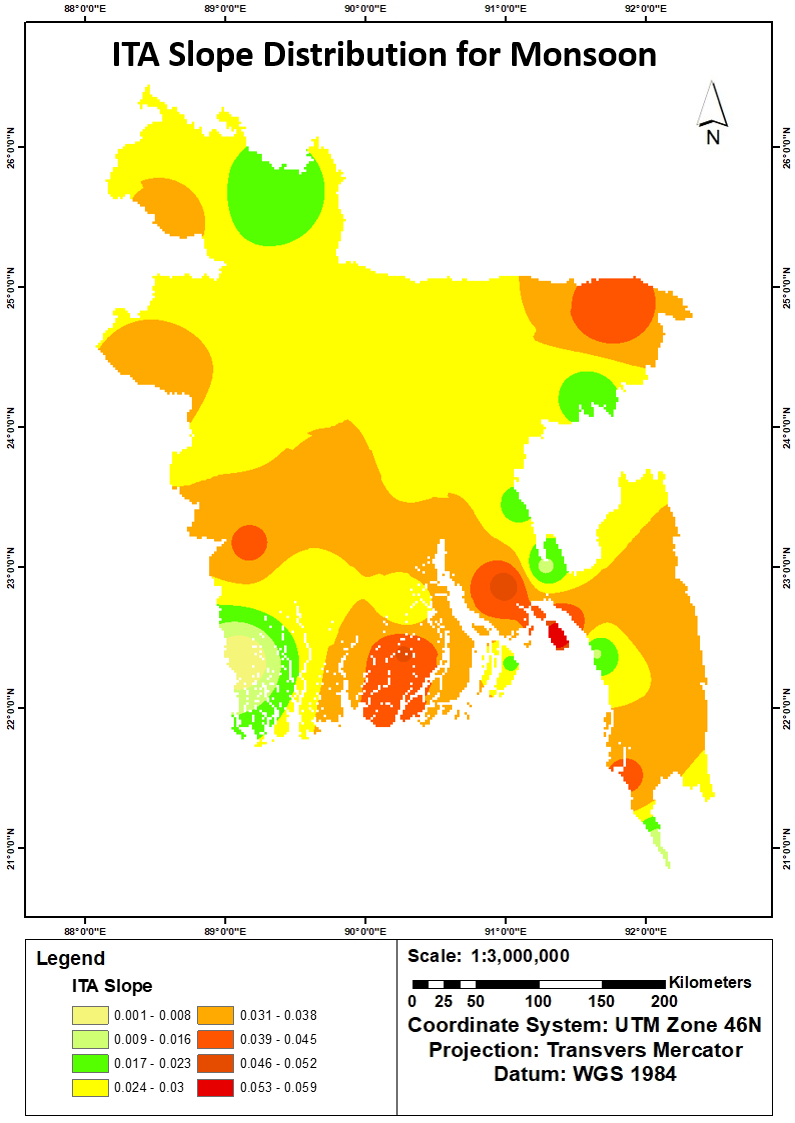 |
| **Monsoon** | | | |
| 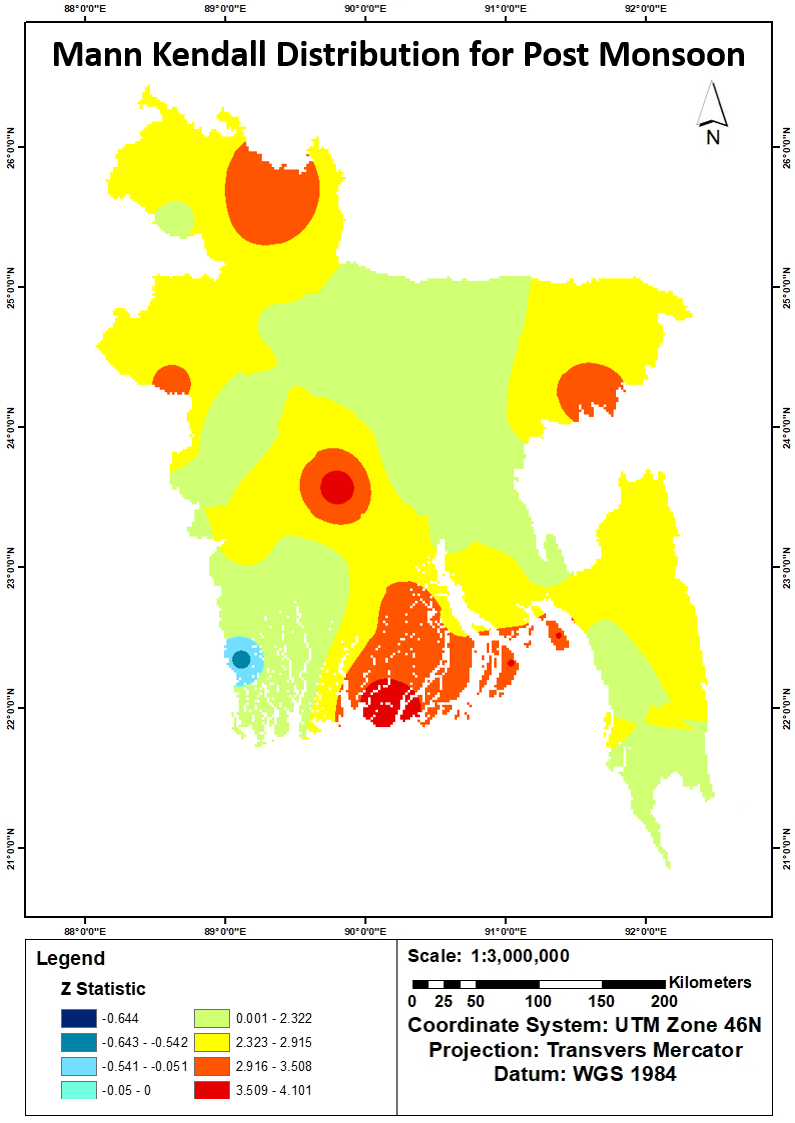 | 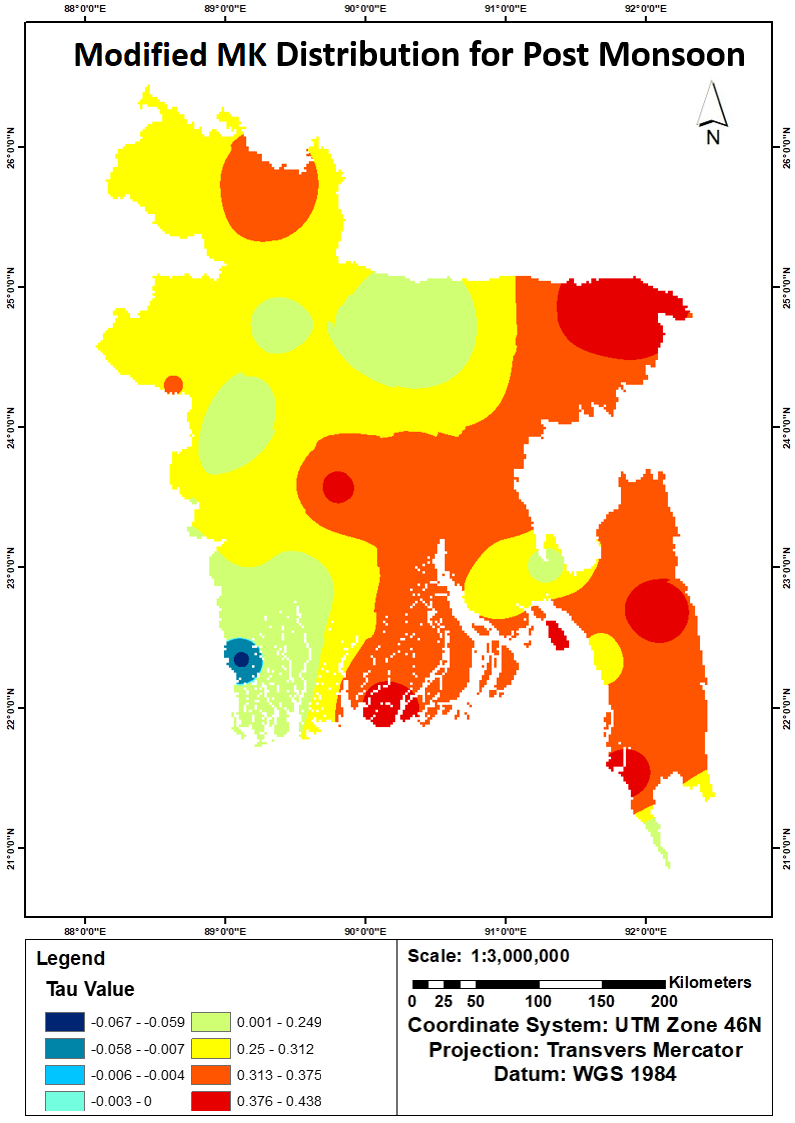 | 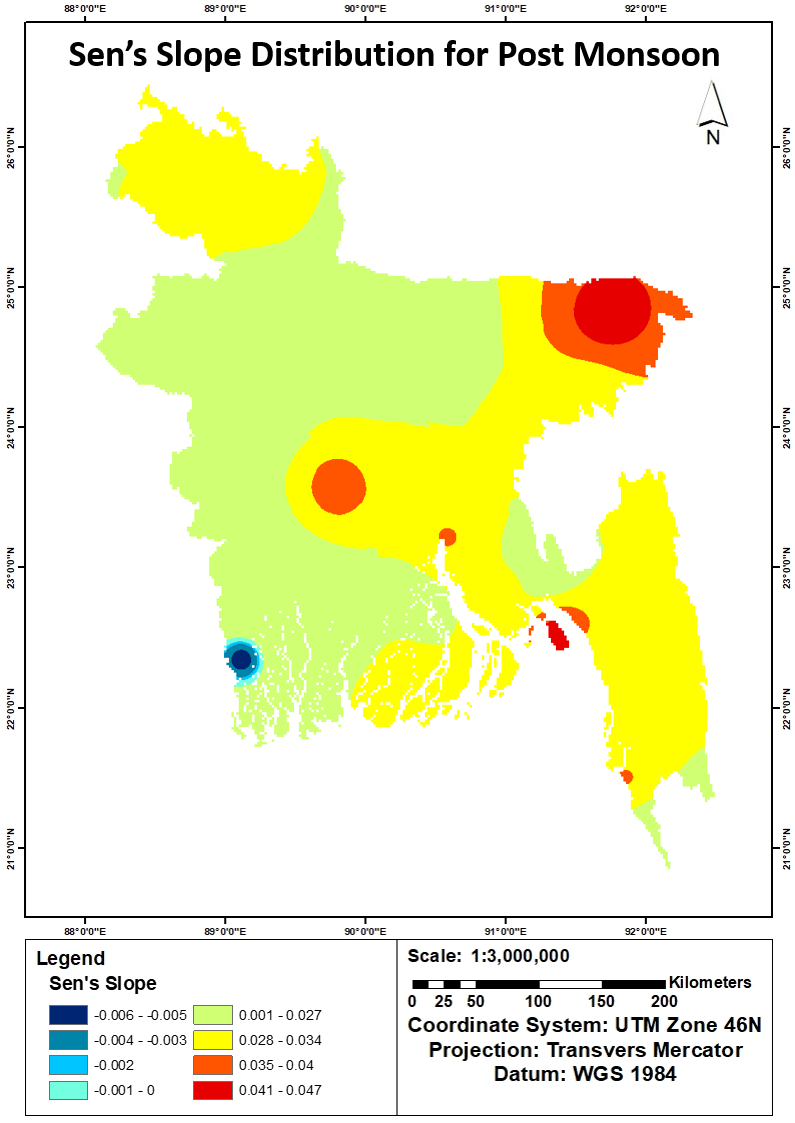 | 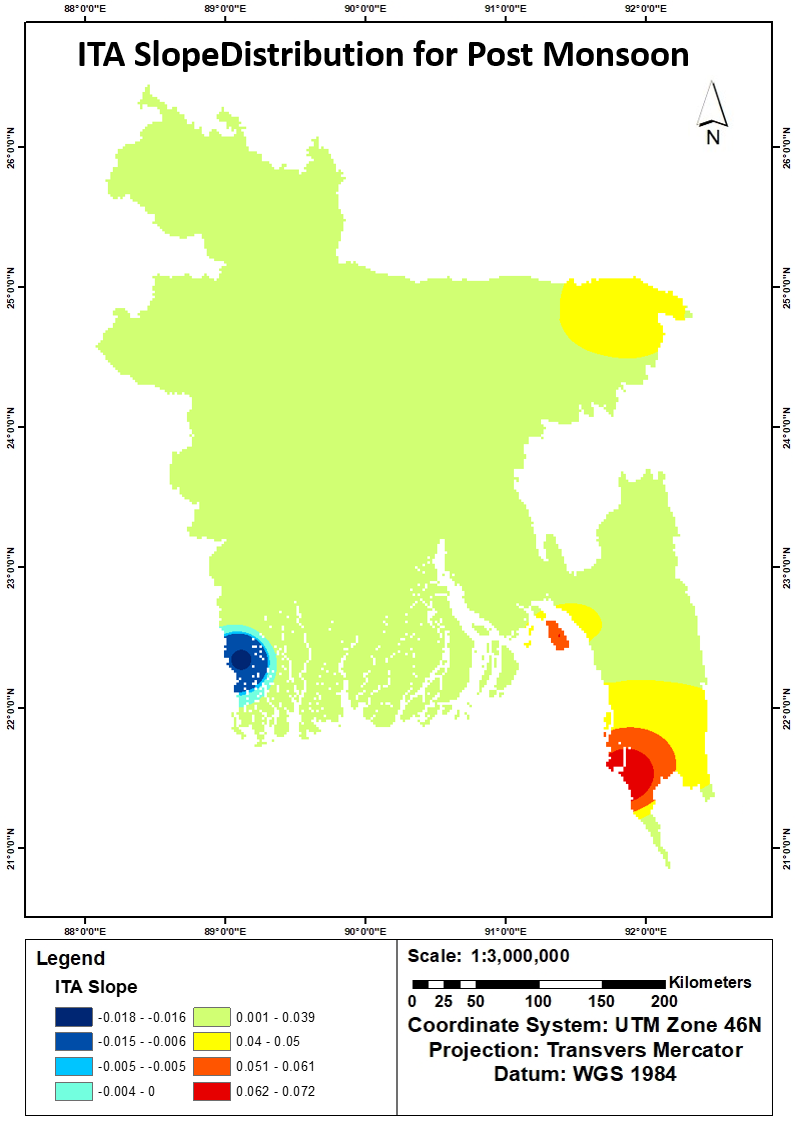 |
| **Post-monsoon** | | | |
| 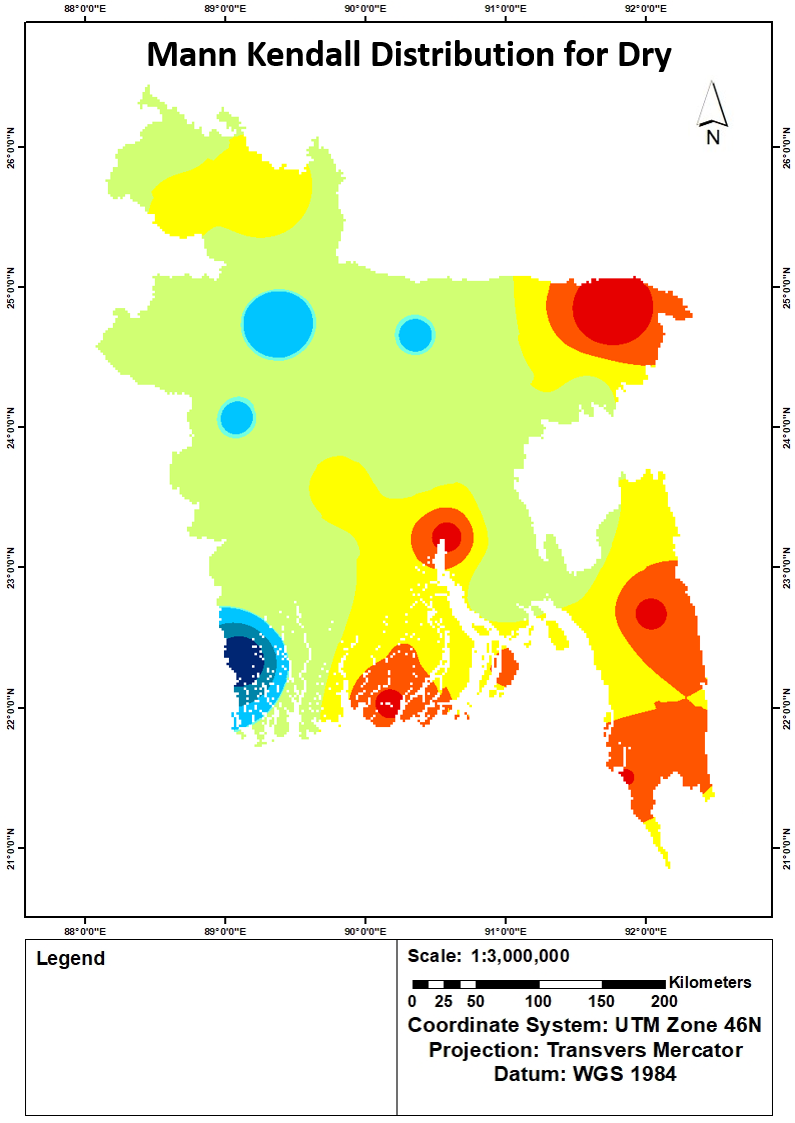 | 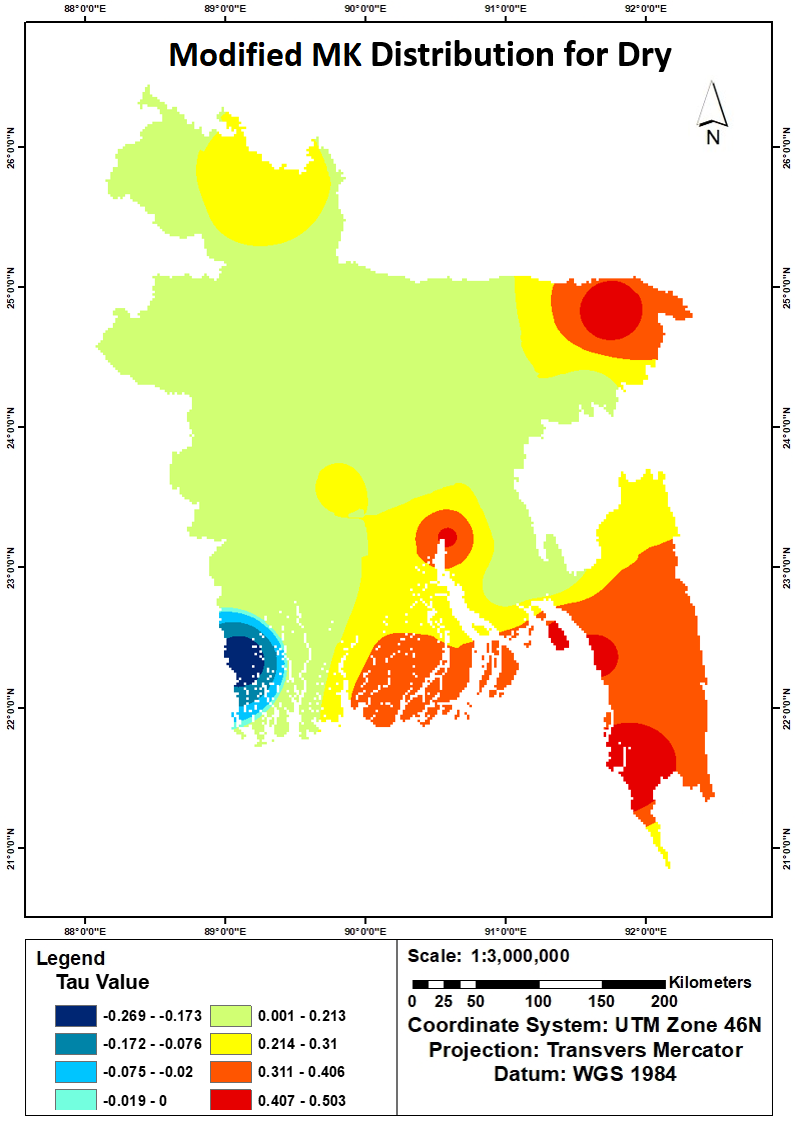 | 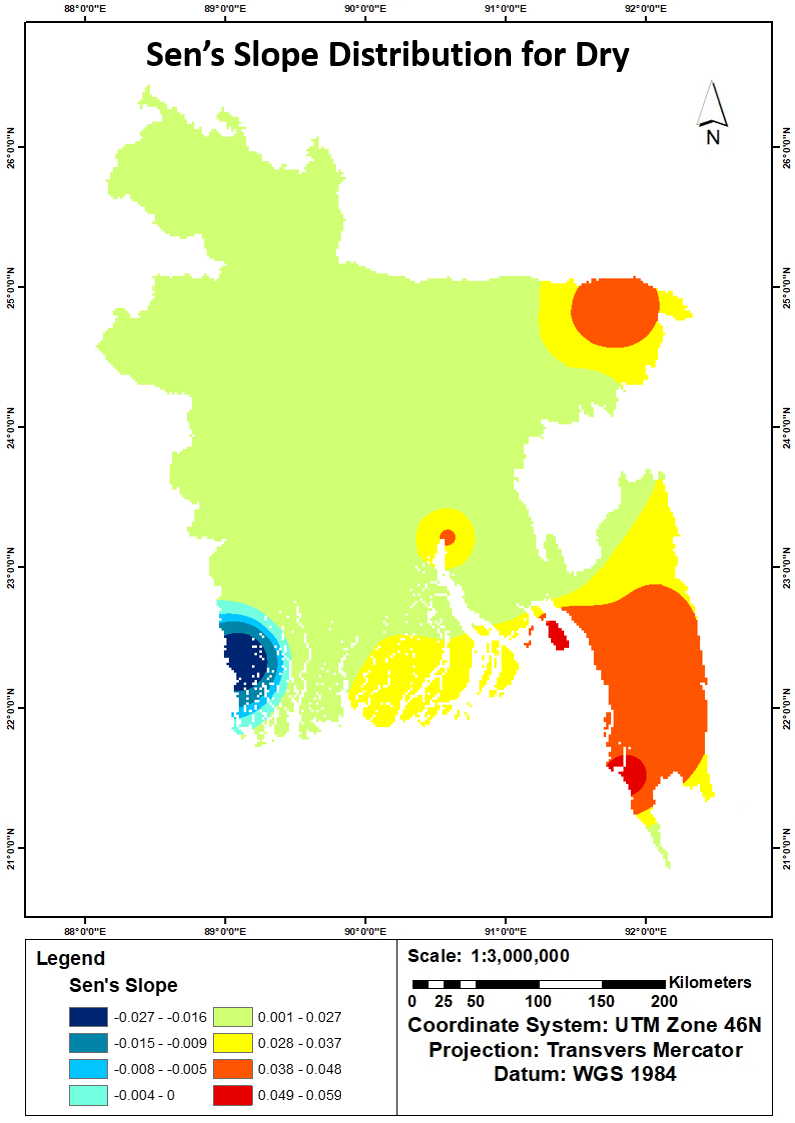 | 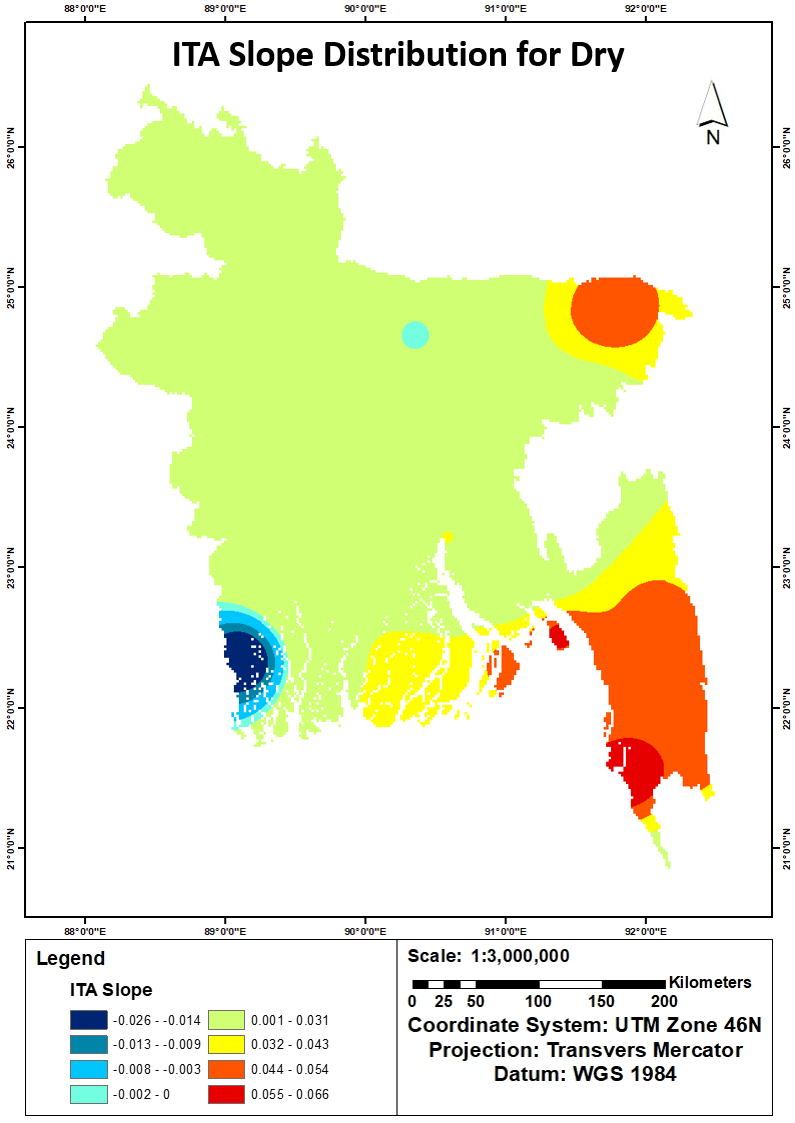 |
| **Dry** | | | |

**Figure S4: Spatial distribution of (a) Annual, (b) Pre-monsoon, (c) Monsoon, (d) Post-monsoon and (e) Dry annual maximum temperature trend using MK (1st column), mMK (2nd column), ITA (3rd column) and Sen’s slope (4th column) analysis**

| 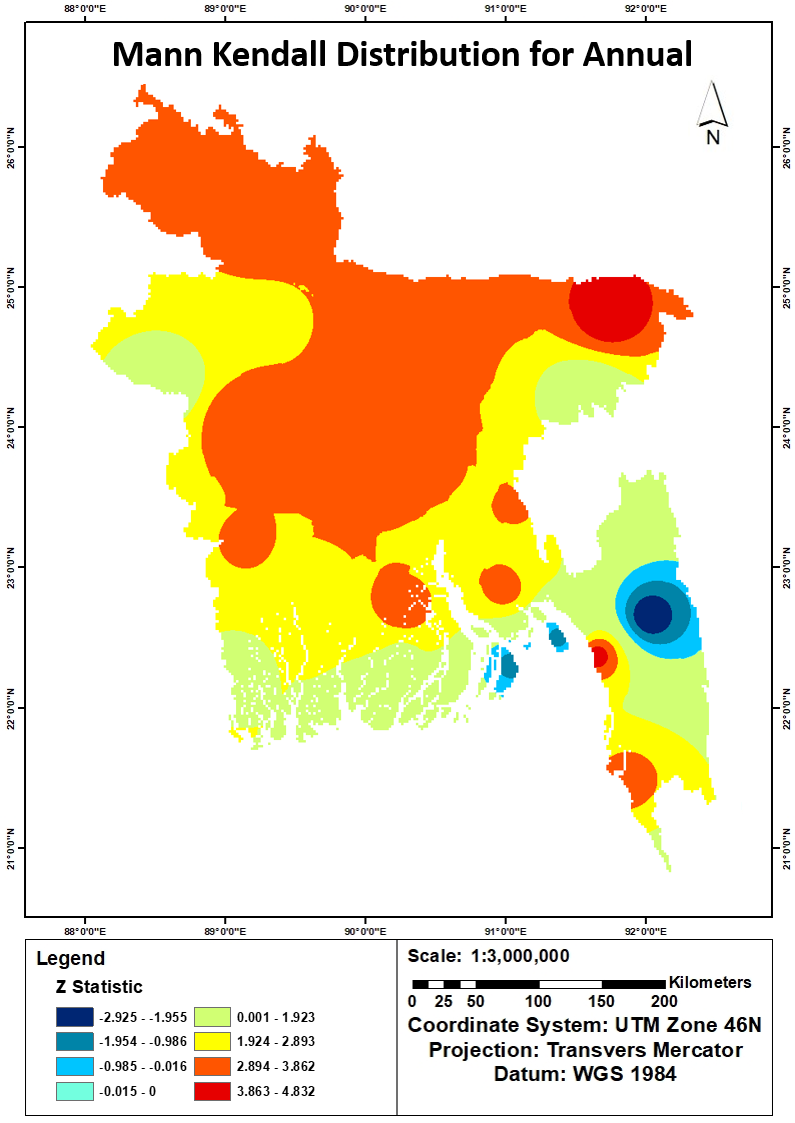 | 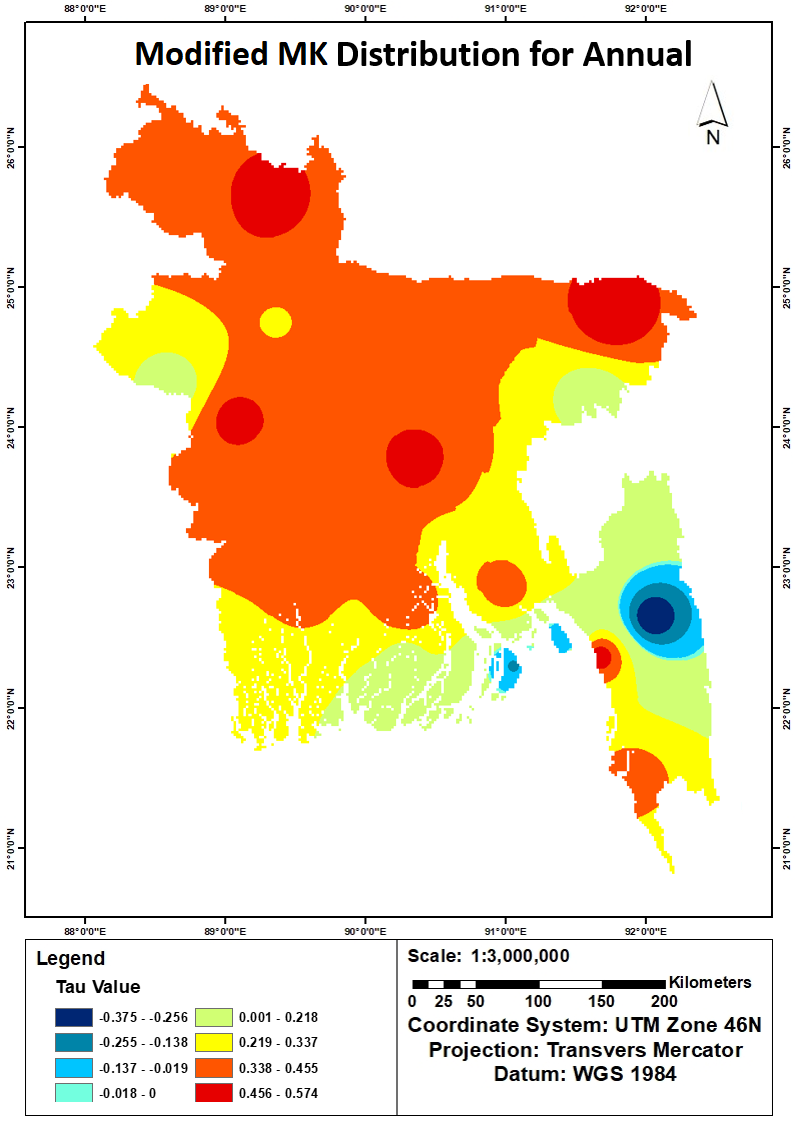 | 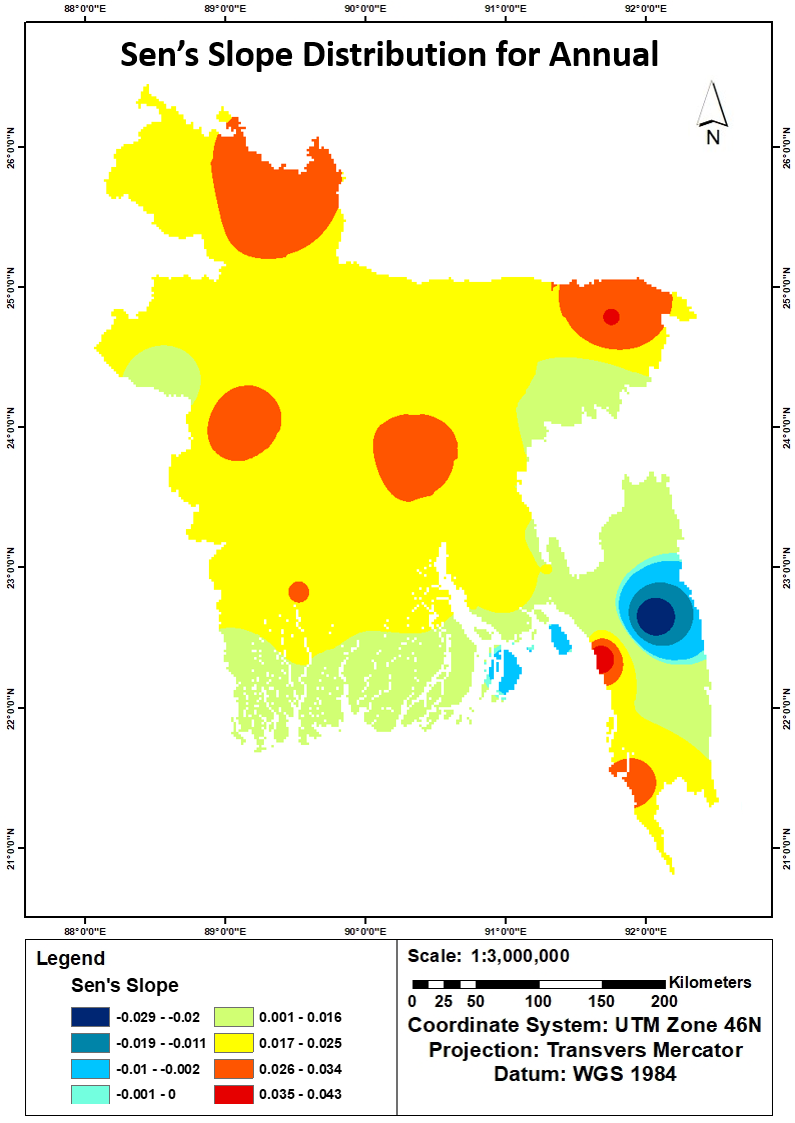 | 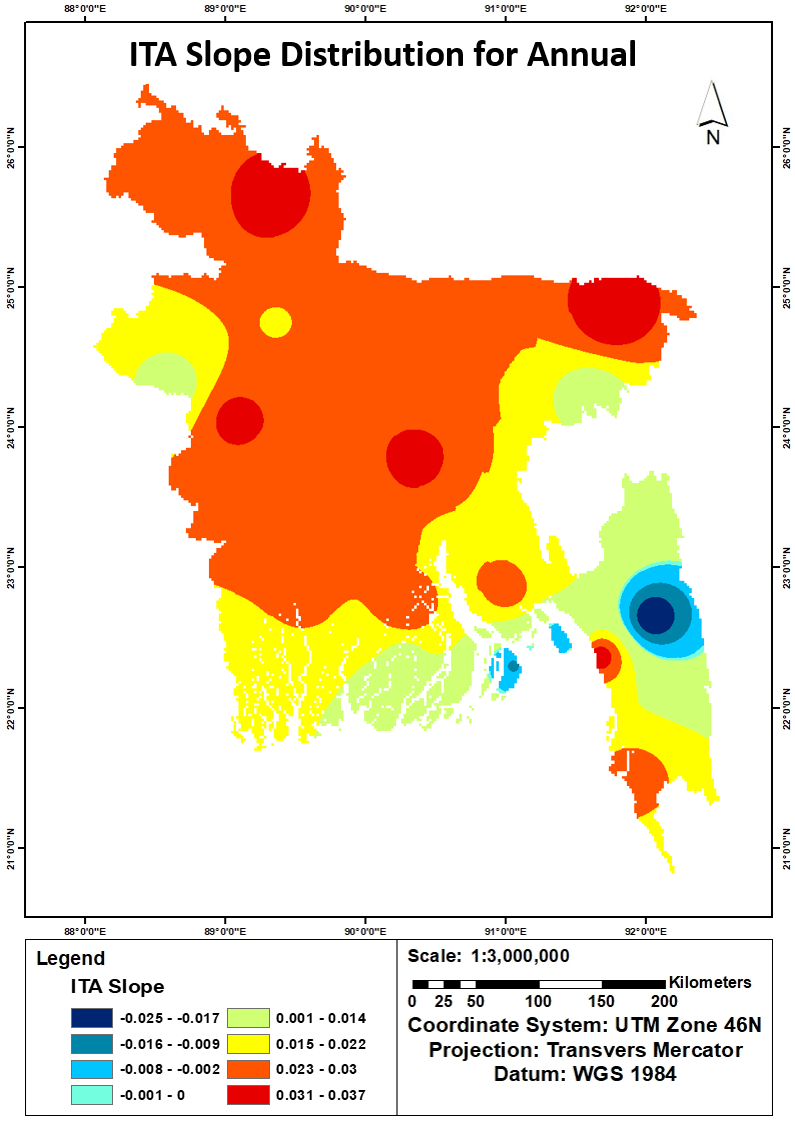 |
| --- | --- | --- | --- |
| **Annual** | | | |
| 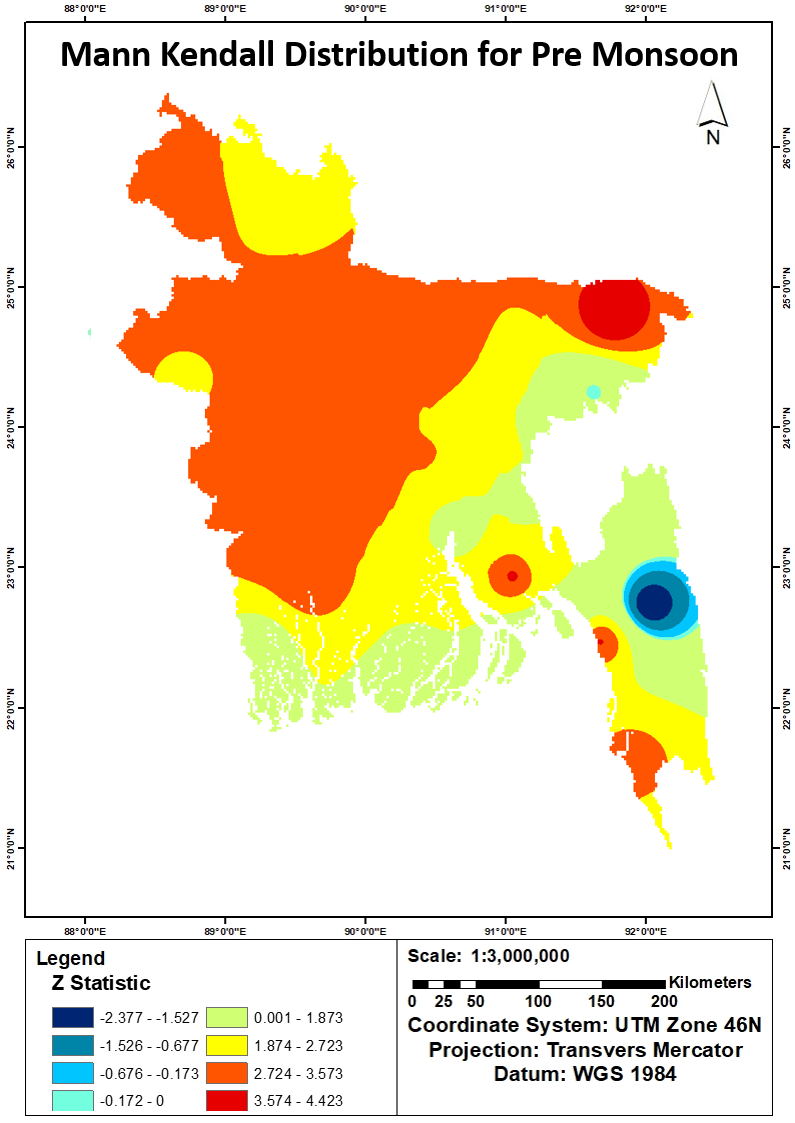 | 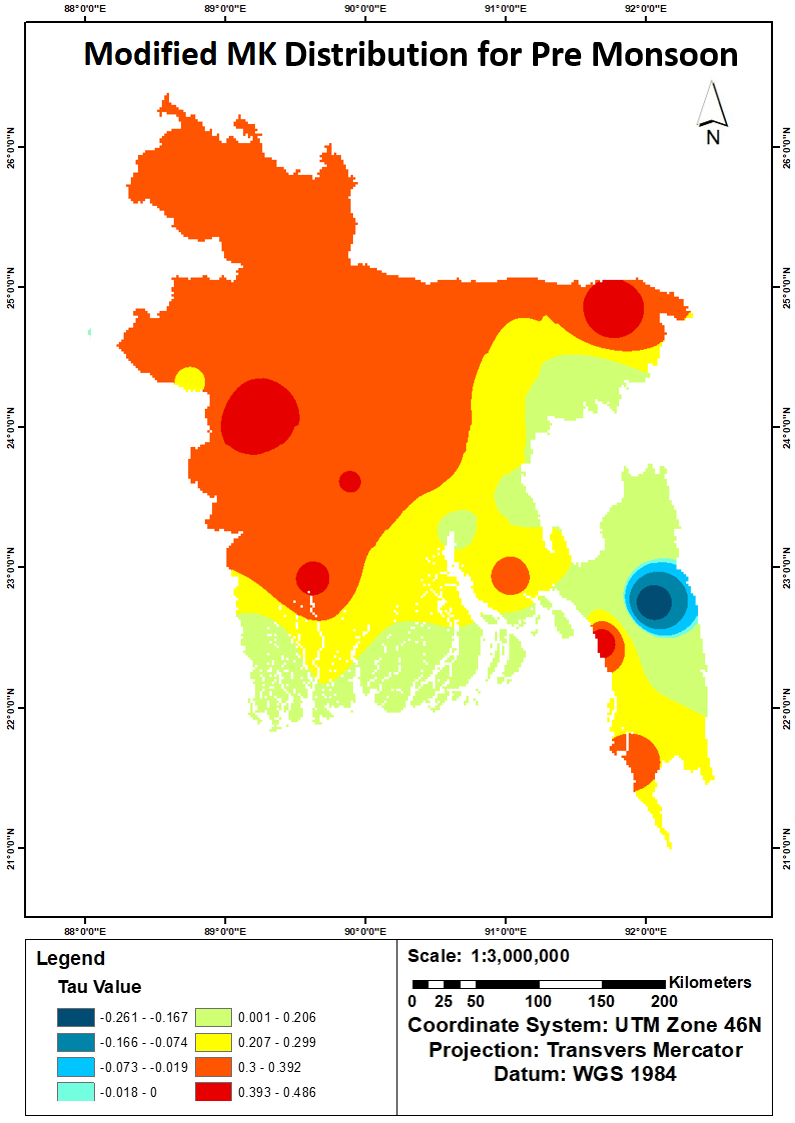 | 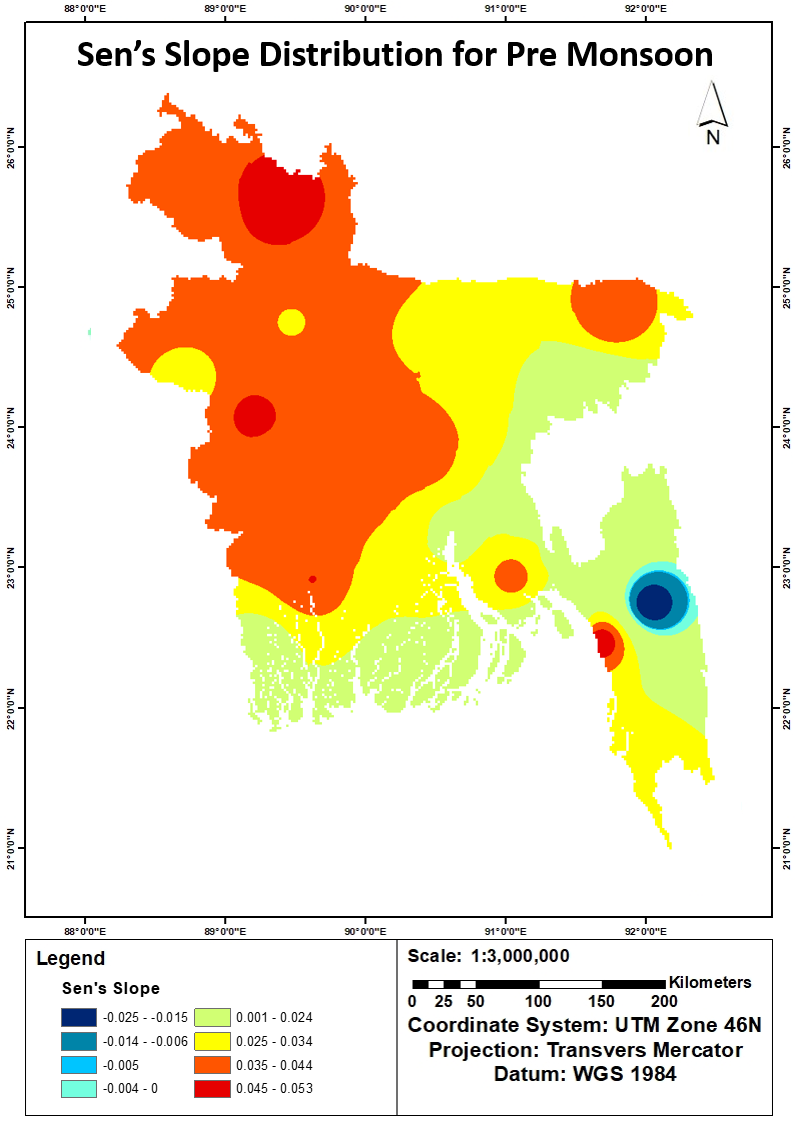 | 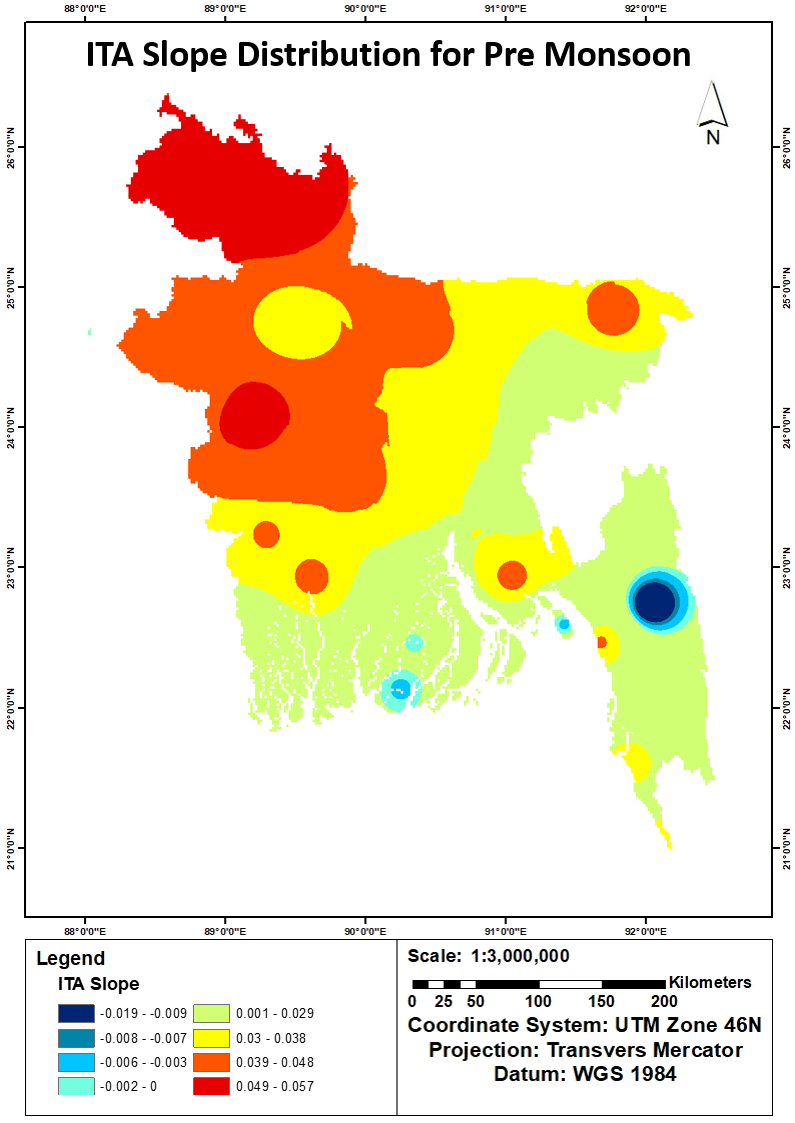 |
| **Pre-monsoon** | | | |
| 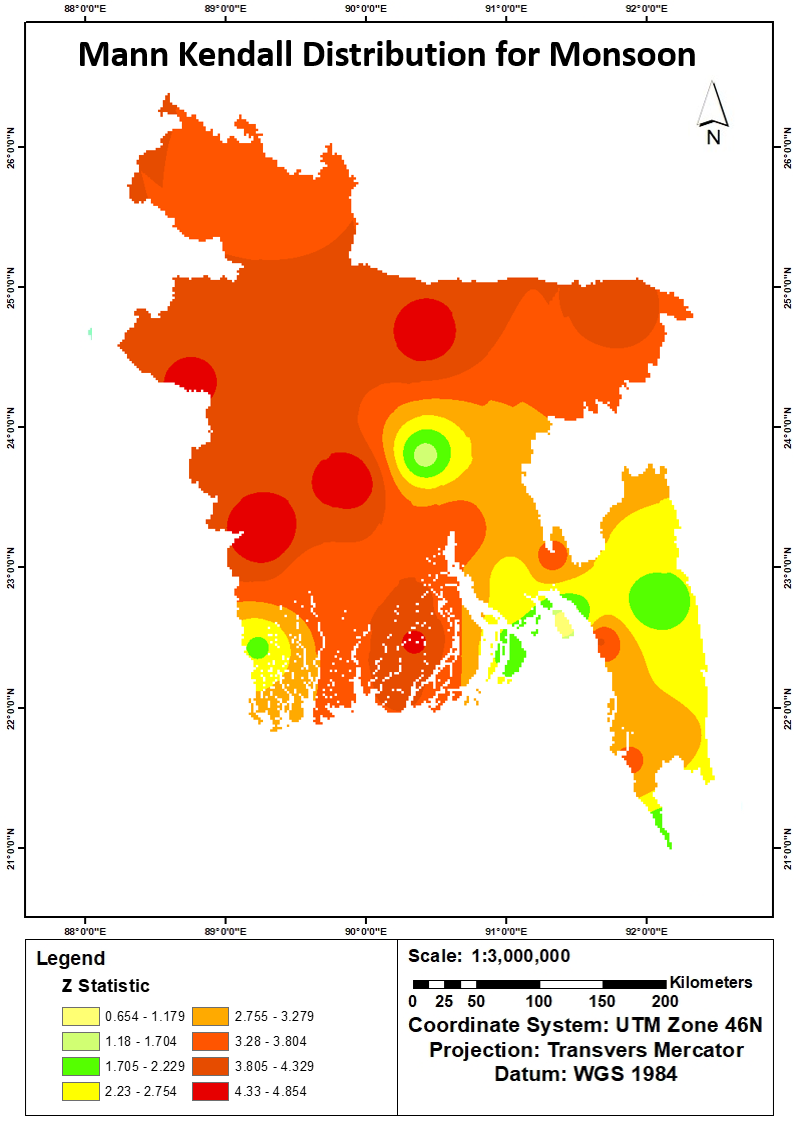 | 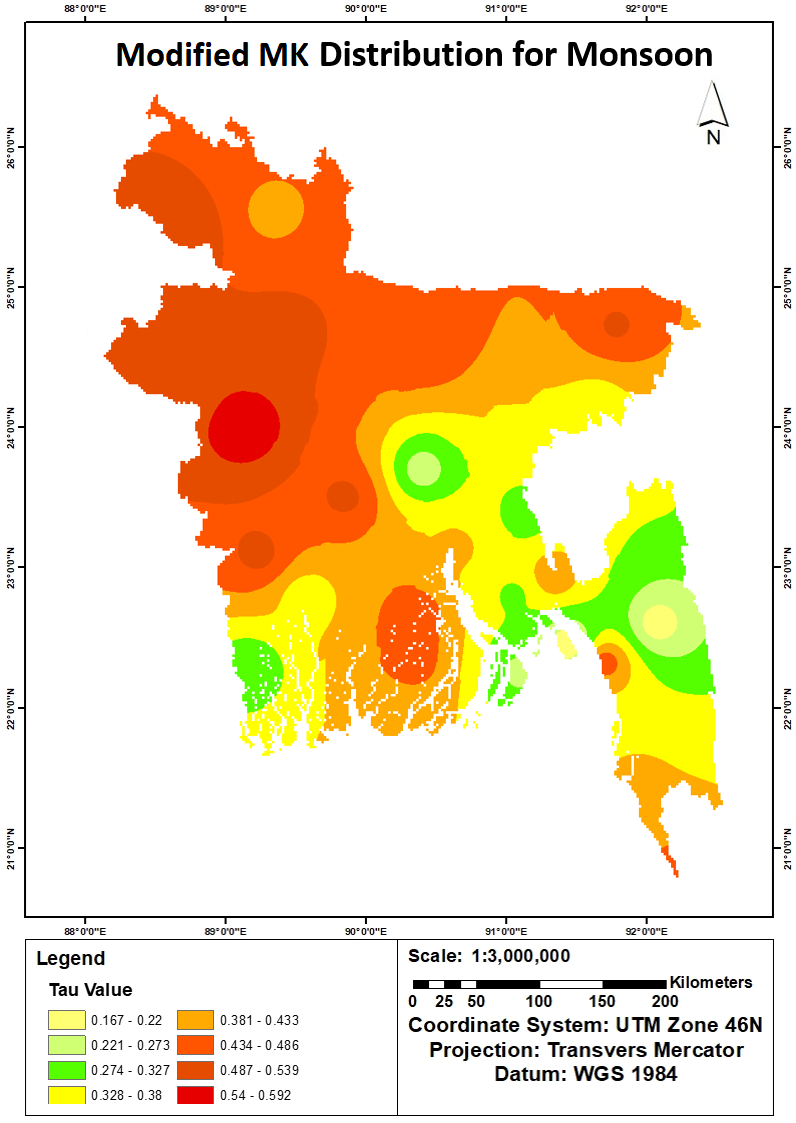 | 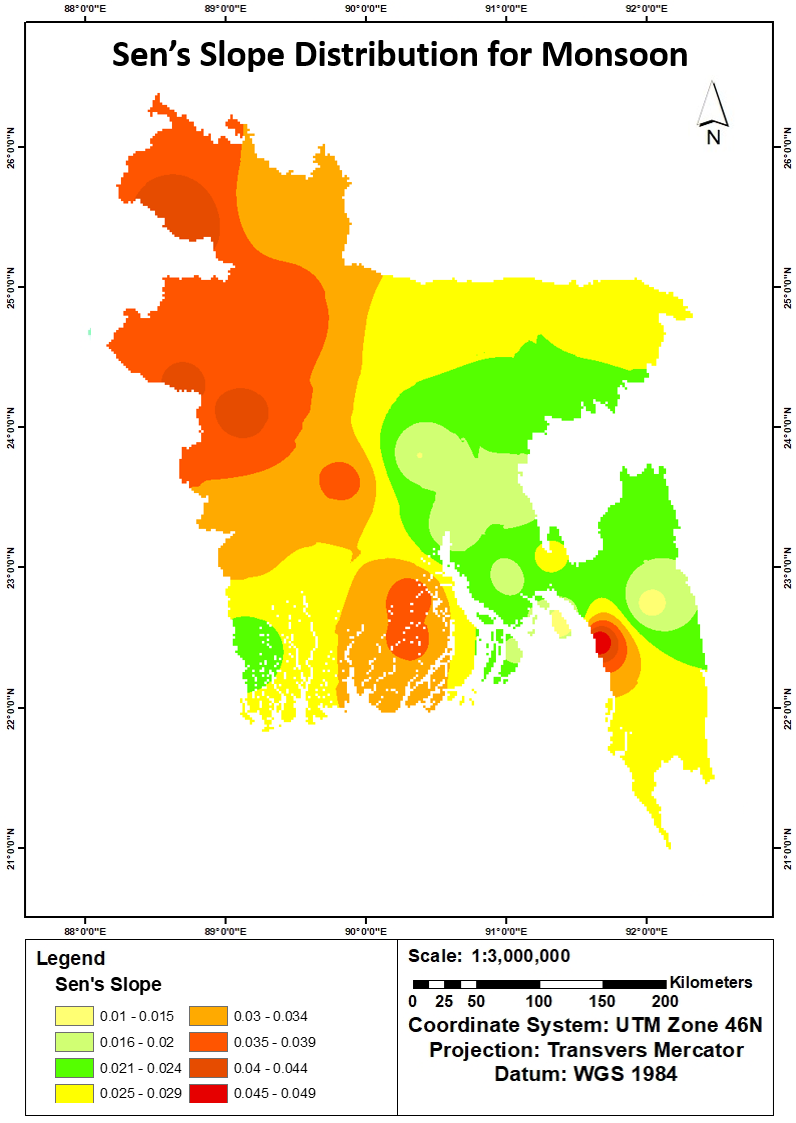 | 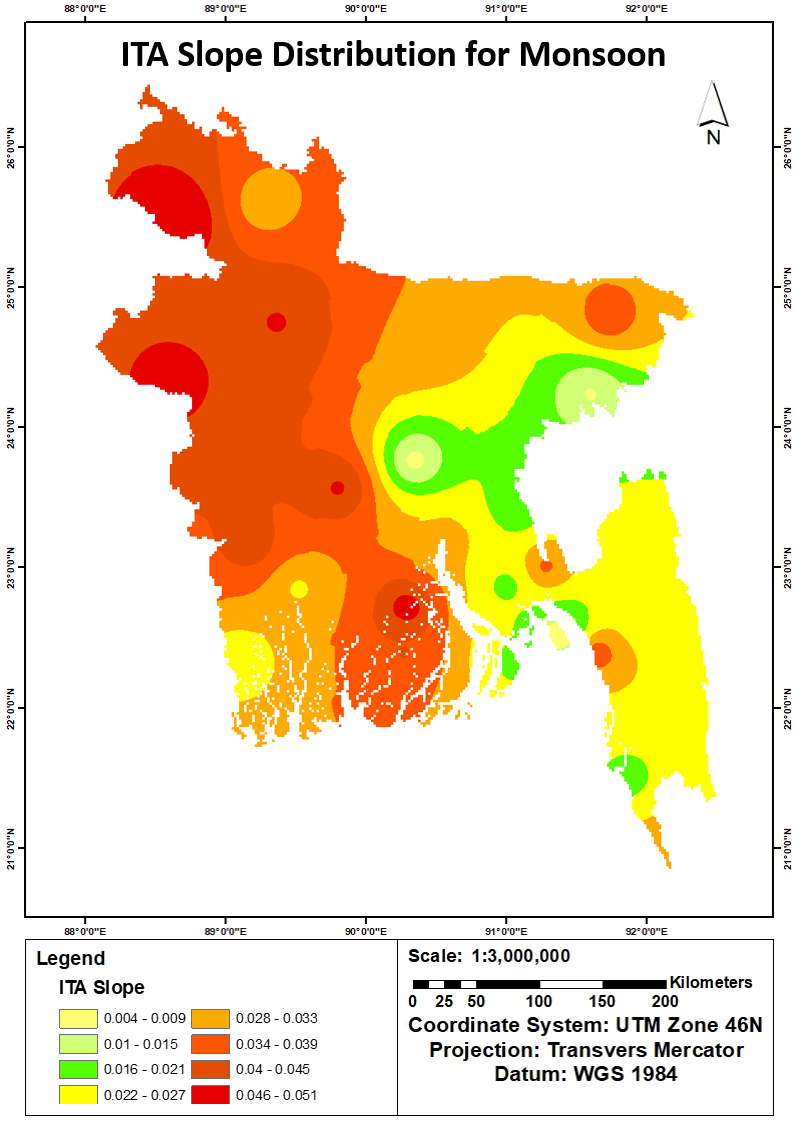 |
| **Monsoon** | | | |
| 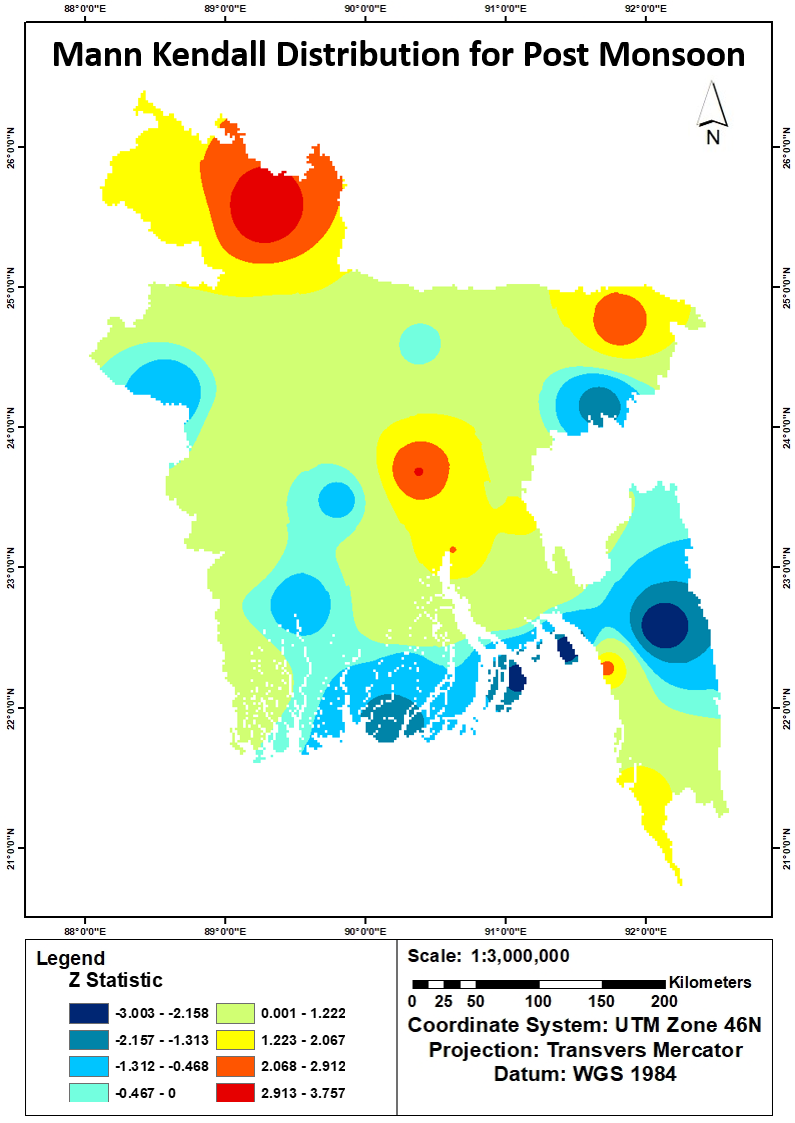 | 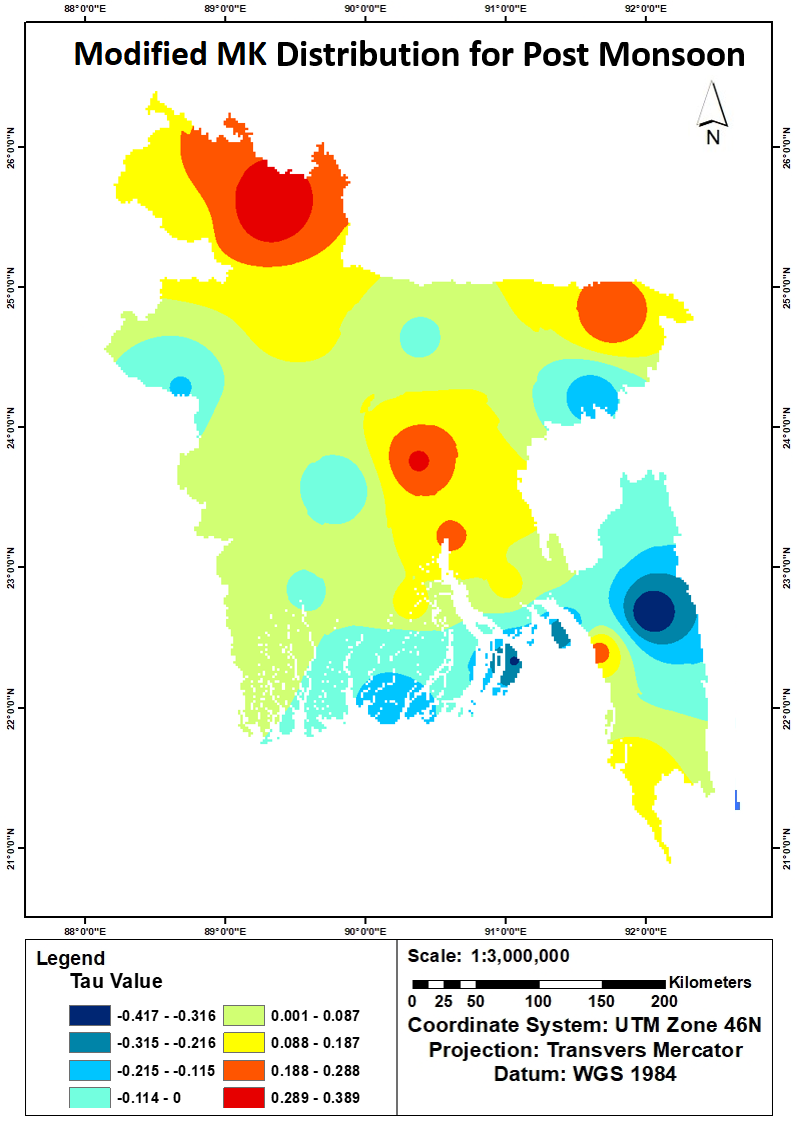 | 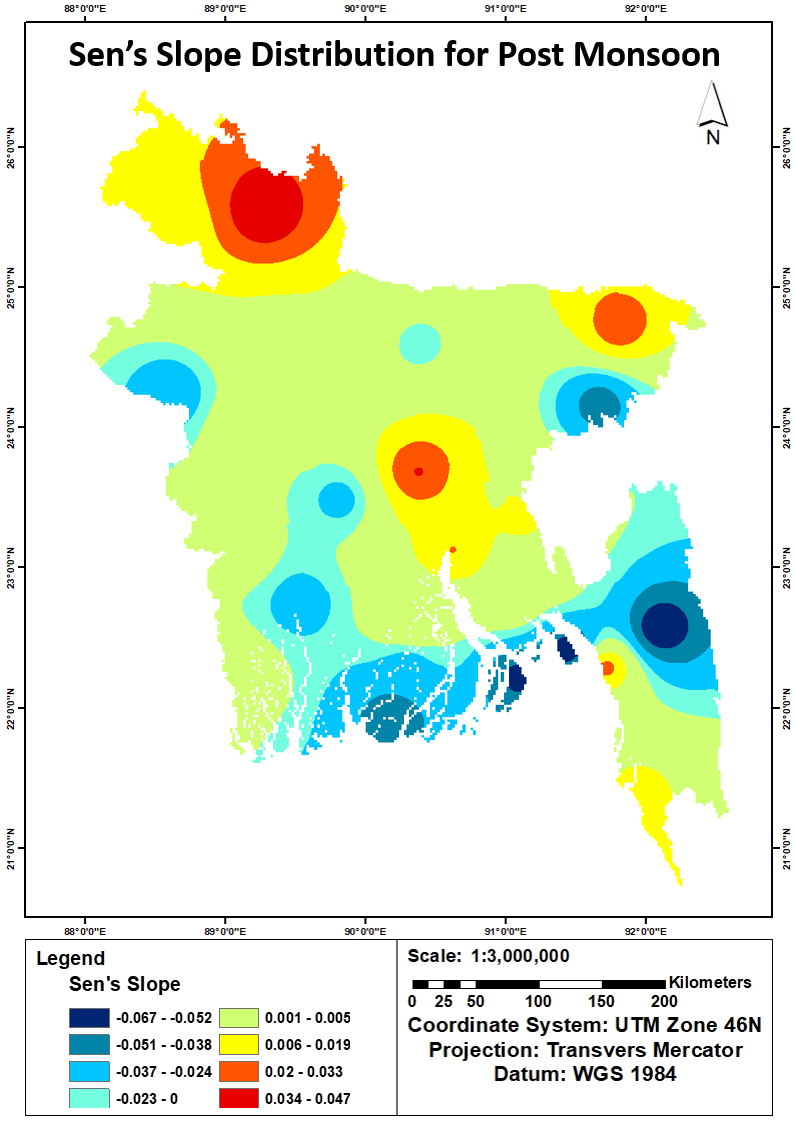 | 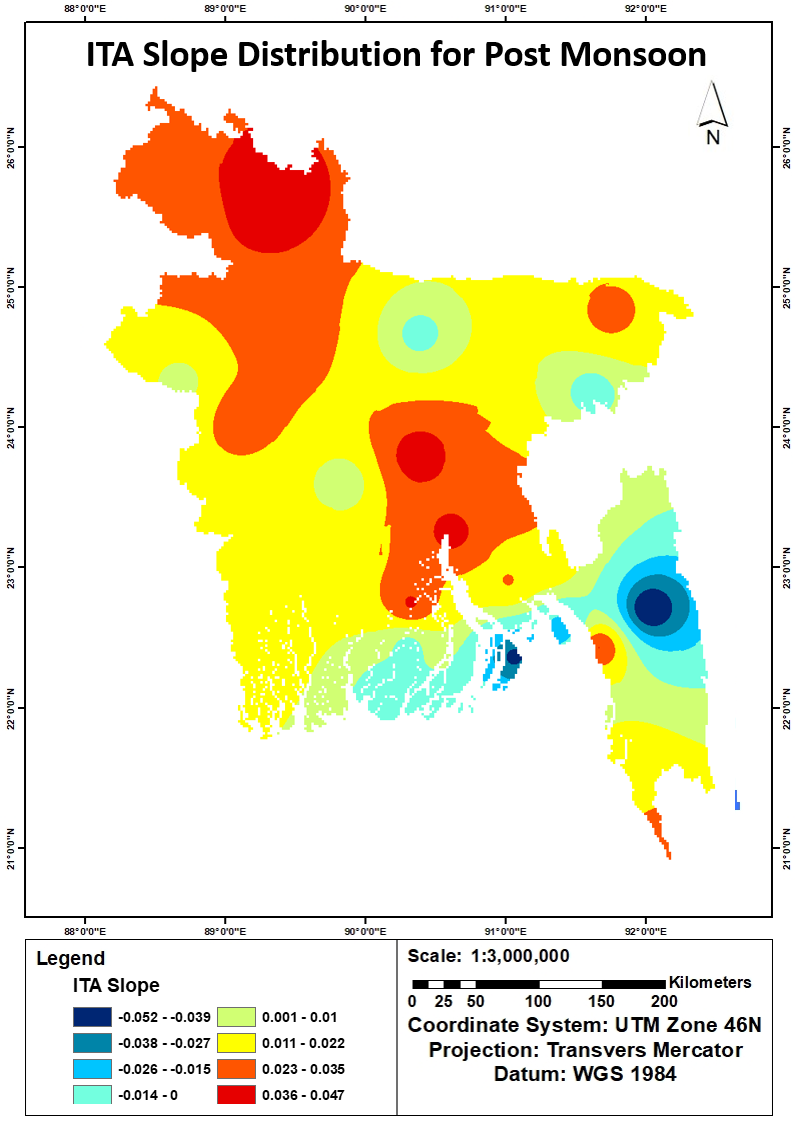 |
| **Post-monsoon** | | | |
| 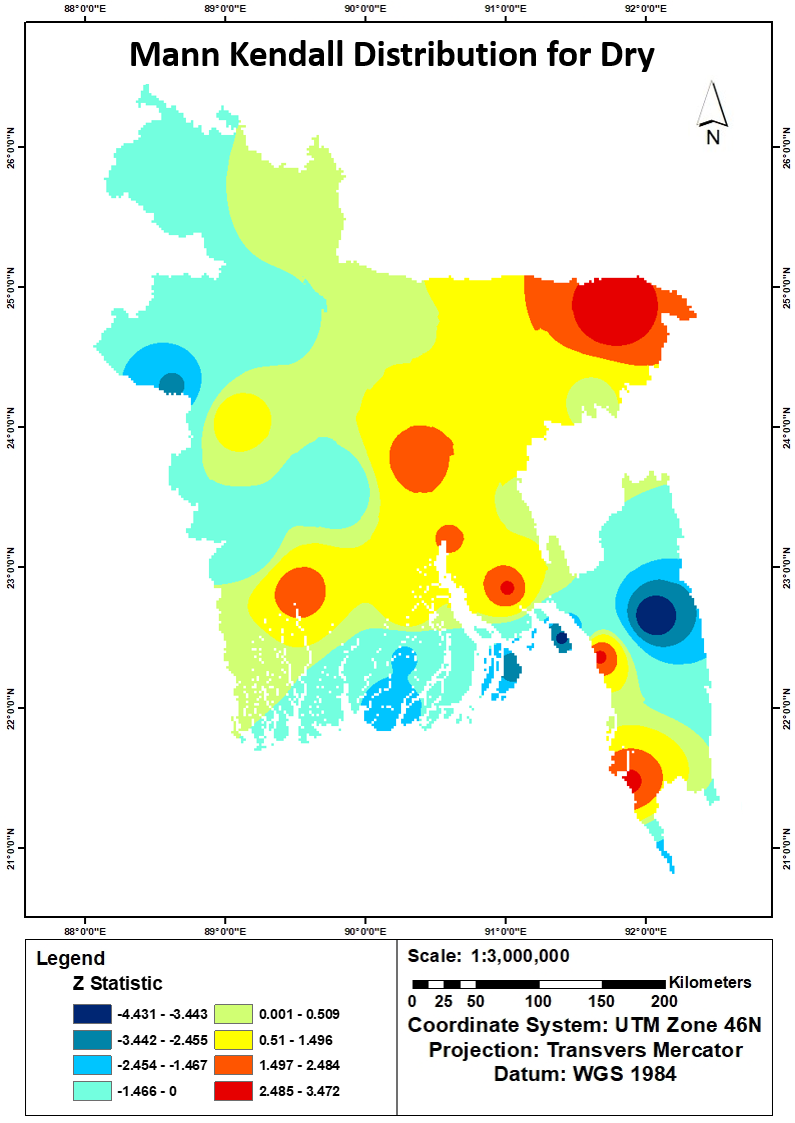 | 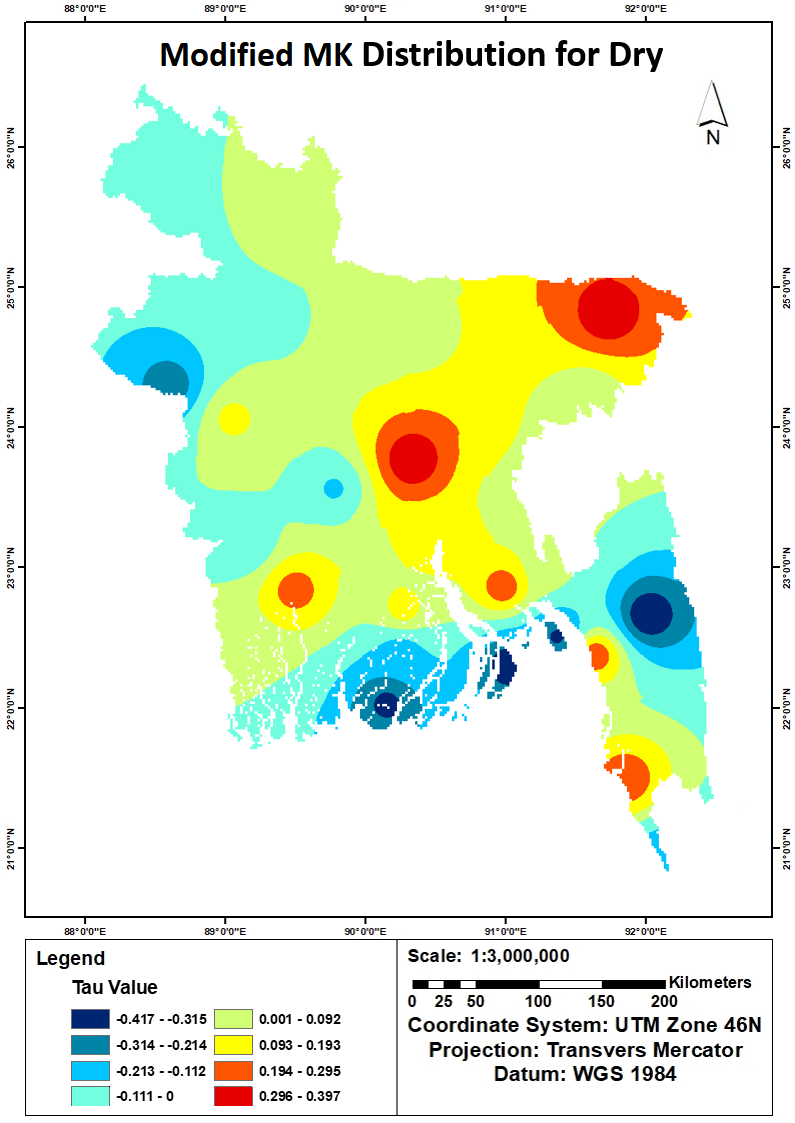 | 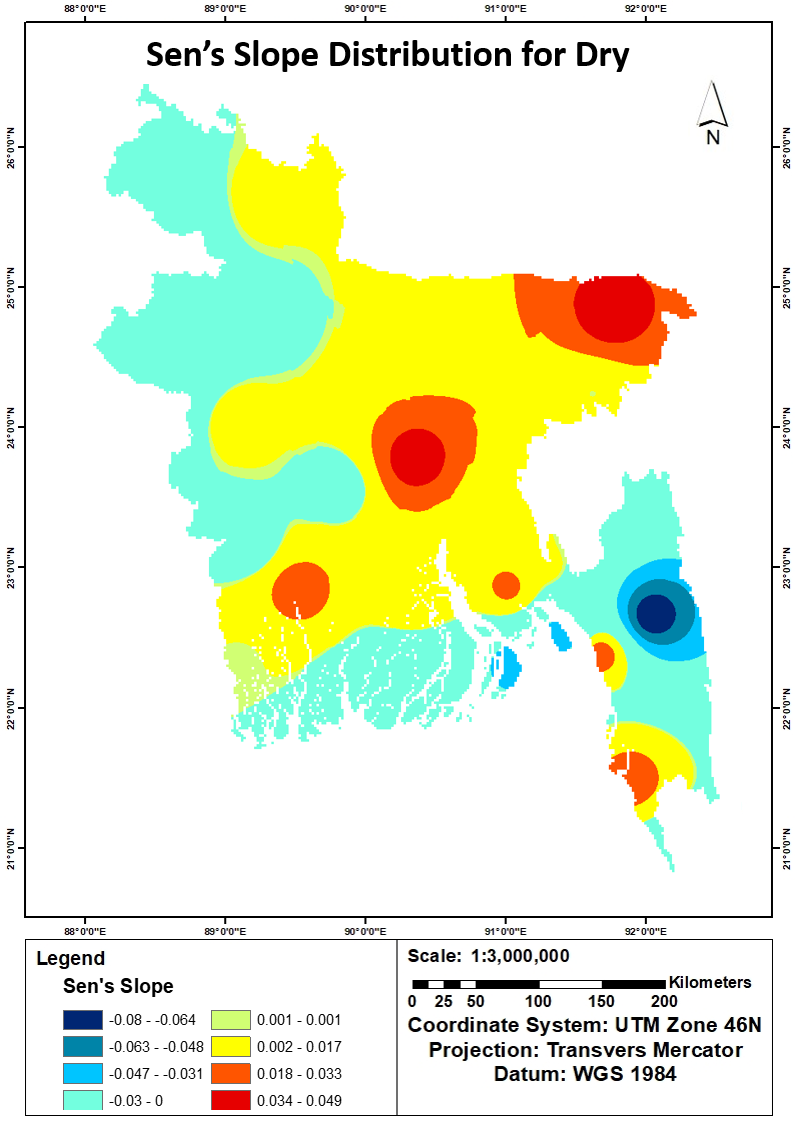 | 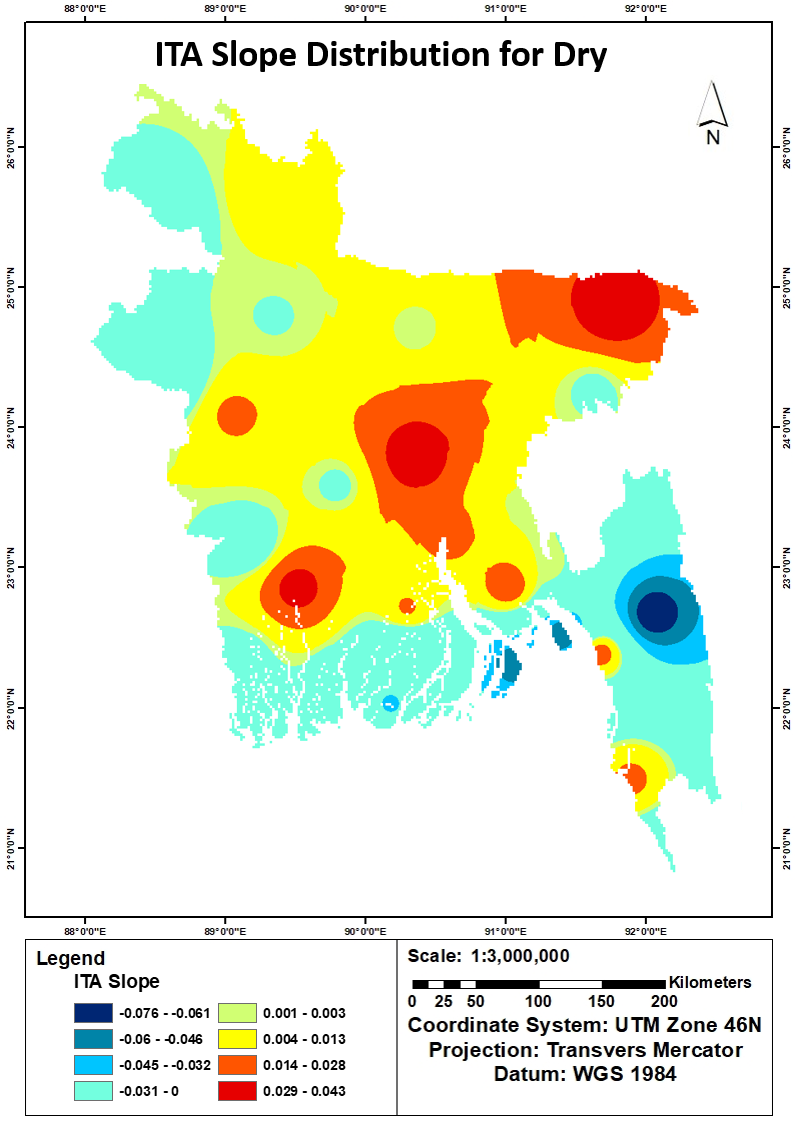 |
| **Dry** | | | |

**Figure S5: Spatial distribution of (a) Annual, (b) Pre-monsoon, (c) Monsoon, (d) Post-monsoon and (e) Dry annual minimum temperature trend using MK (1st column), mMK (2nd column), ITA (3rd column) and Sen’s slope (4th column) analysis**

| 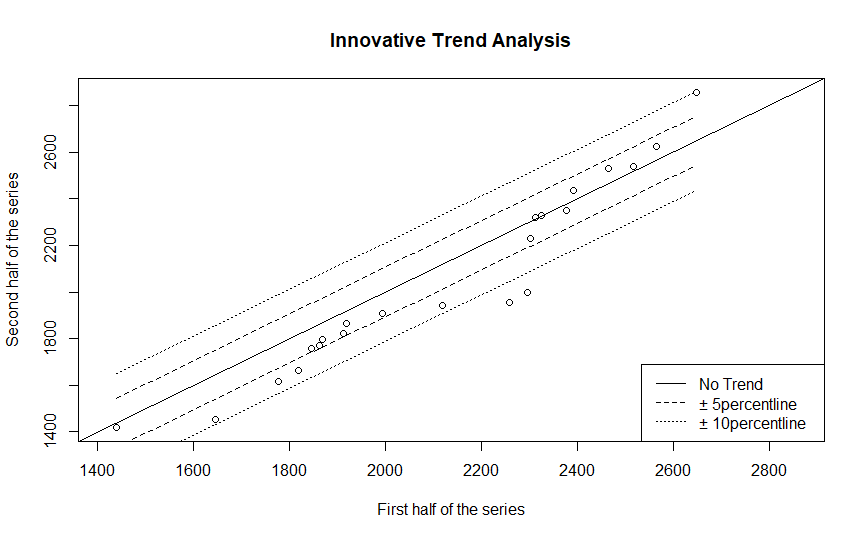 | 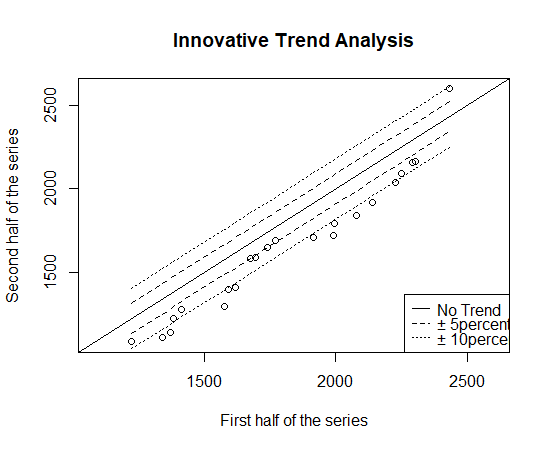 | 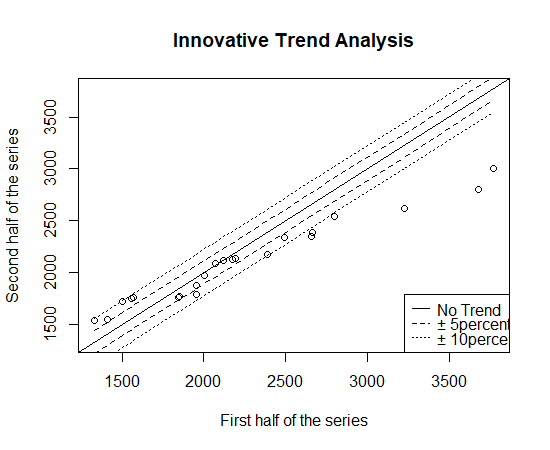 | 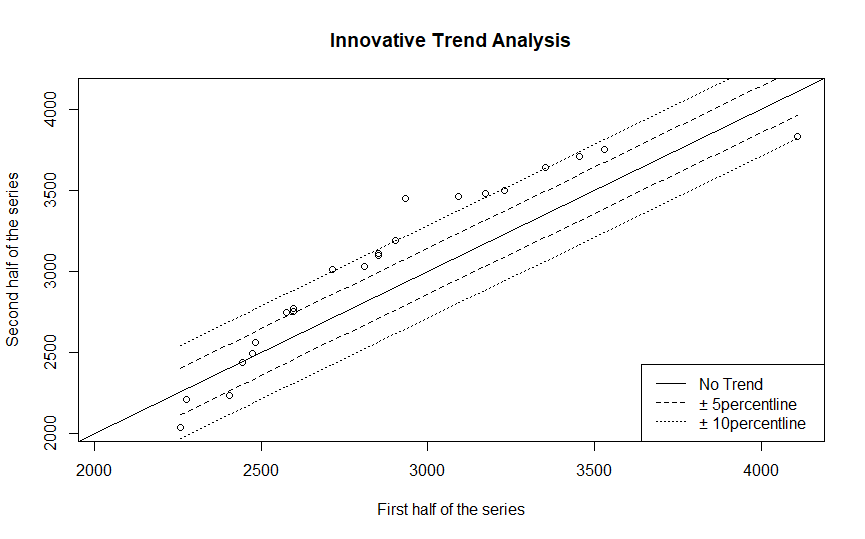 |
| --- | --- | --- | --- |
| (a) Barisal | (b) Bogra | (c) Chandpur | (d) Chittagong |
| 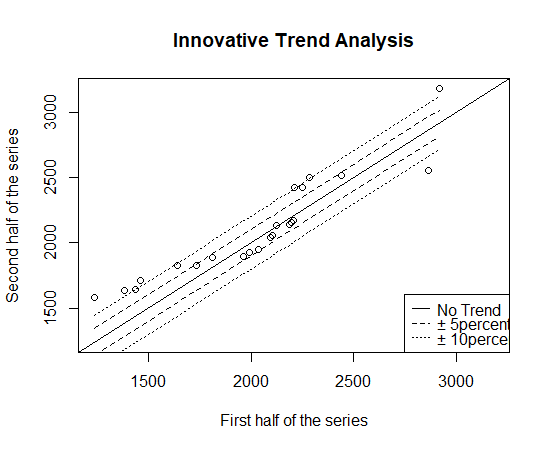 | 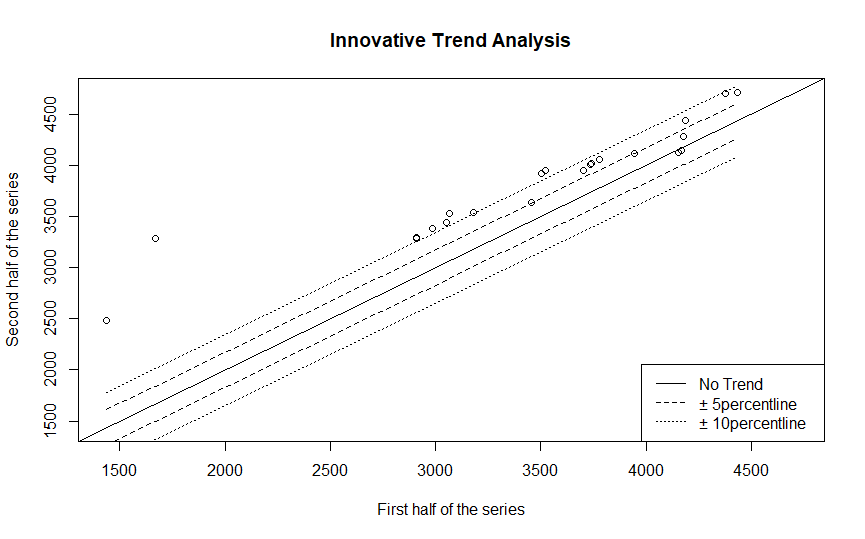 | 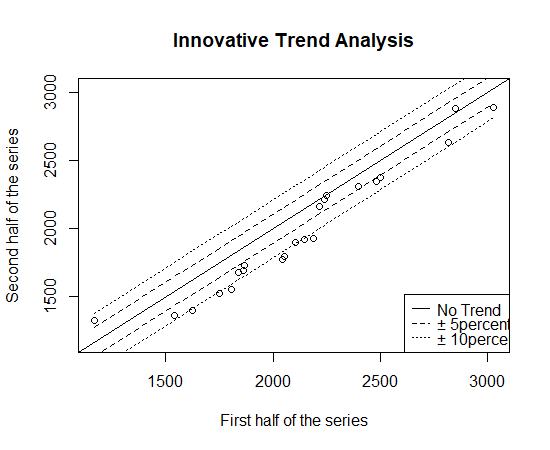 | 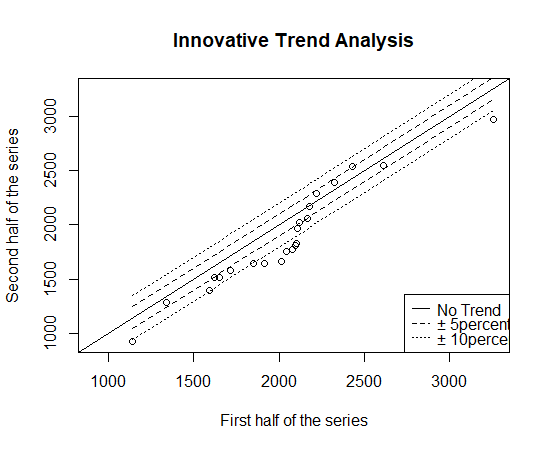 |
| (e) Comilla | (f) Cox’s Bazar | (g) Dhaka | (h) Dinajpur |
| 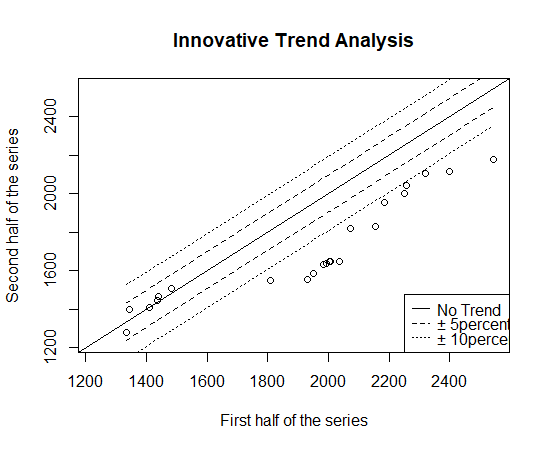 | 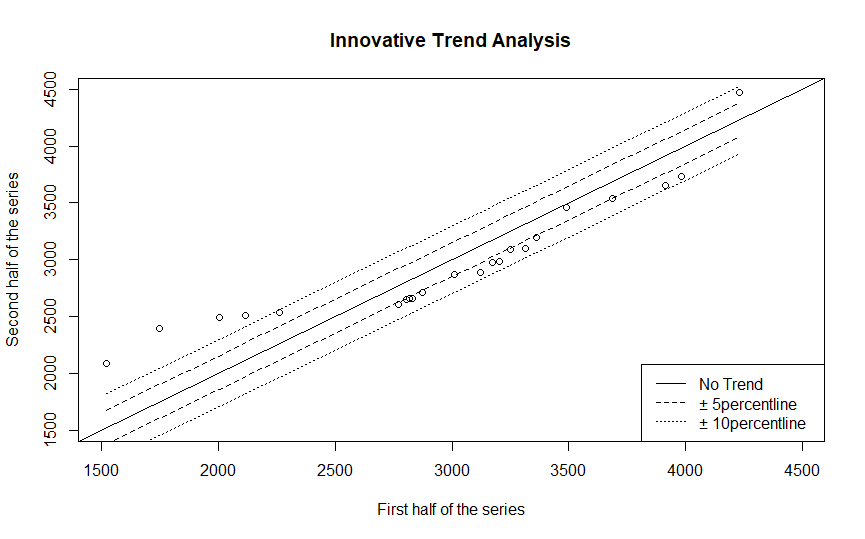 | 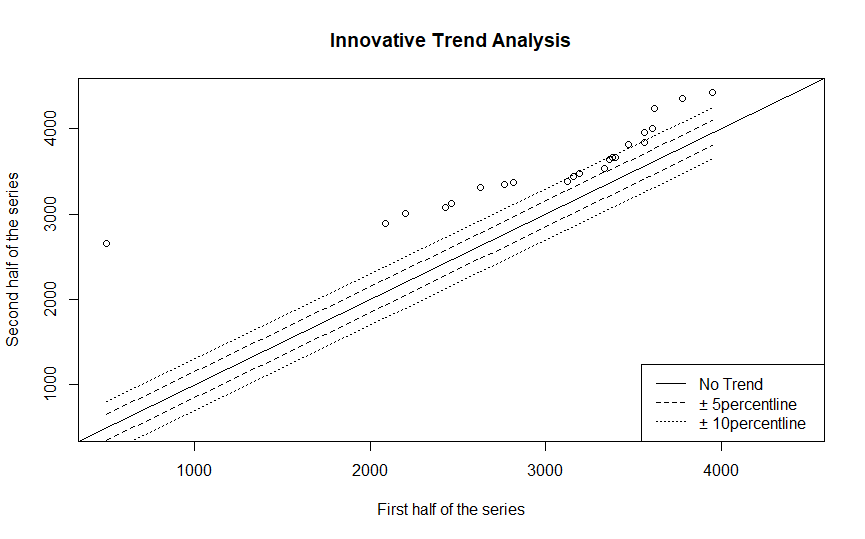 | 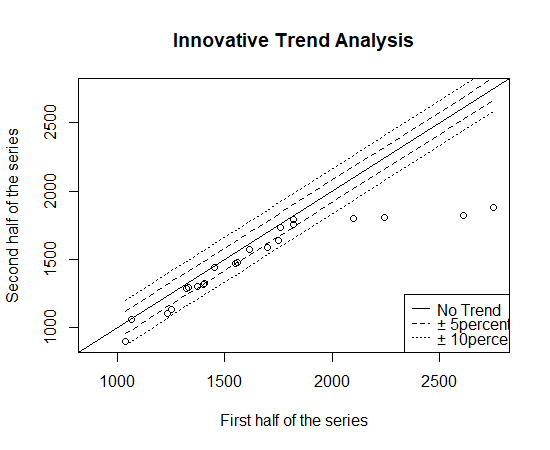 |
| (i) Faridpur | (j) Feni | (k) Hatiya | (l) Ishurdi |
| 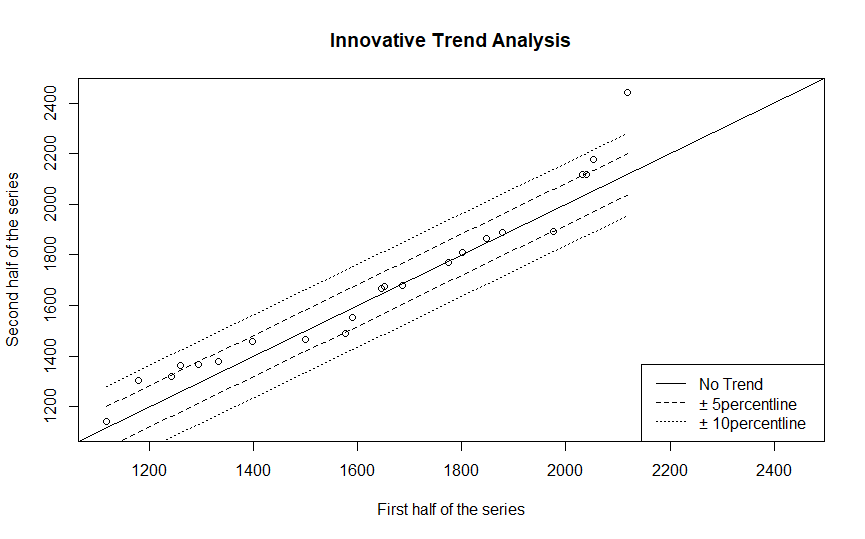 | 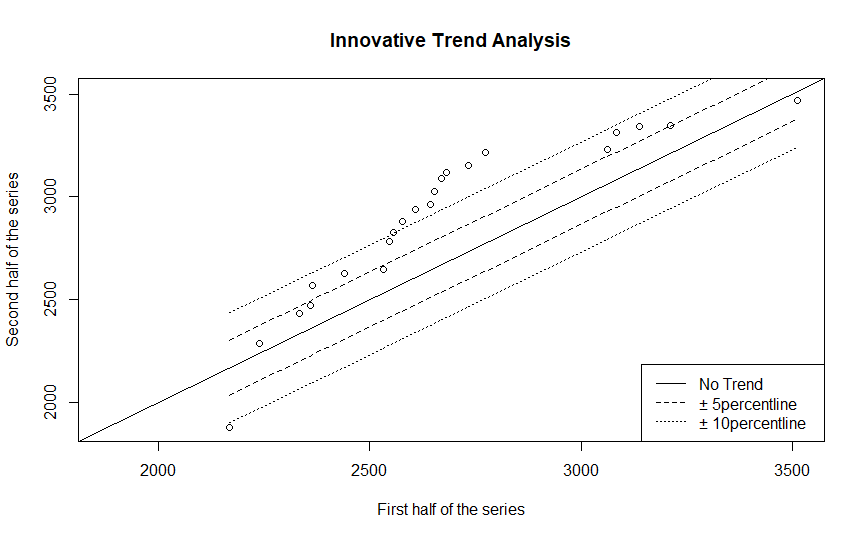 | 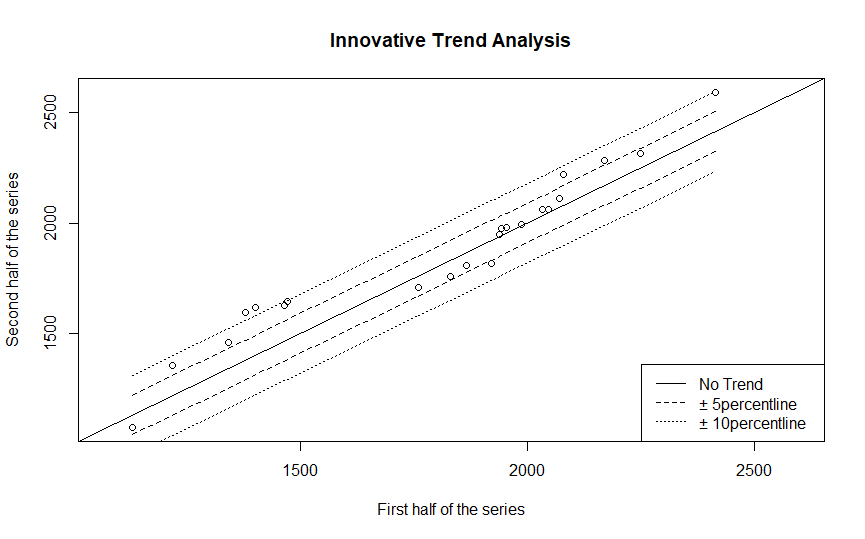 | 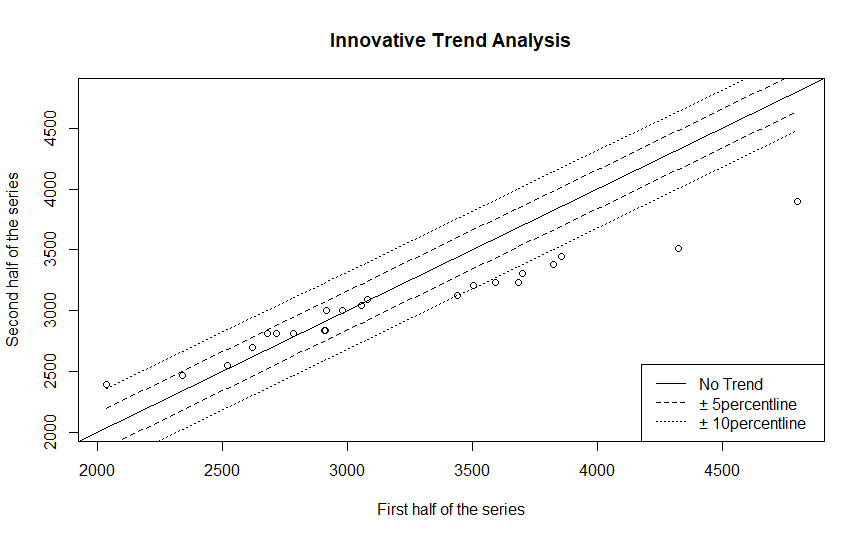 |
| (m) Jessore | (n) Khepupara | (o) Khulna | (p) M. Court |
| 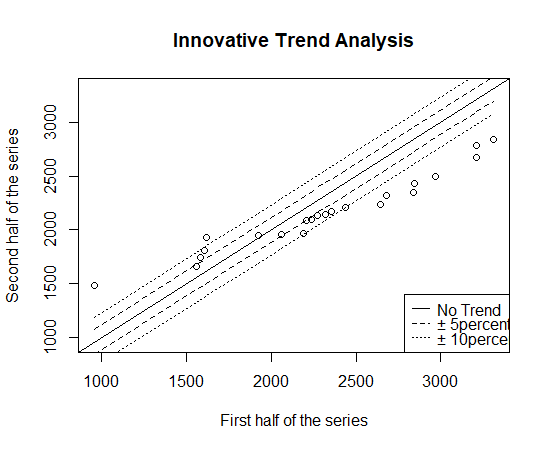 | 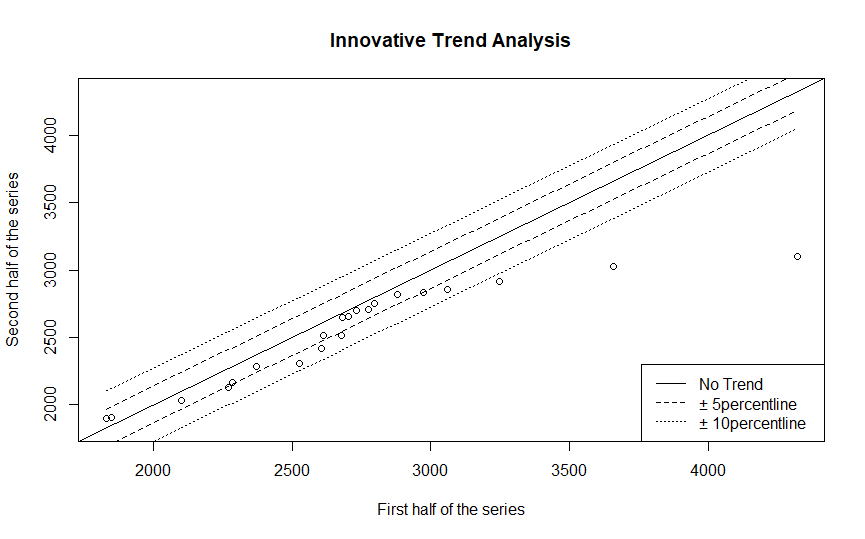 | 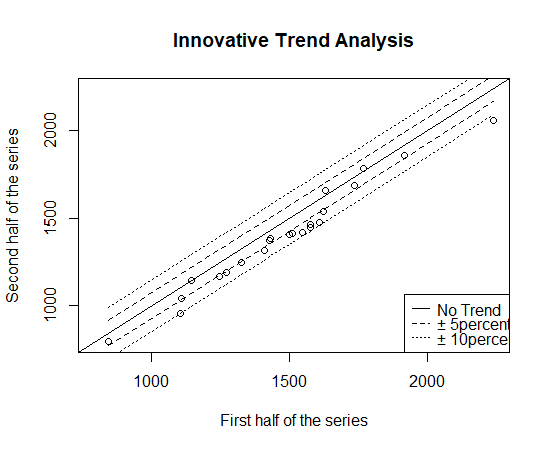 | 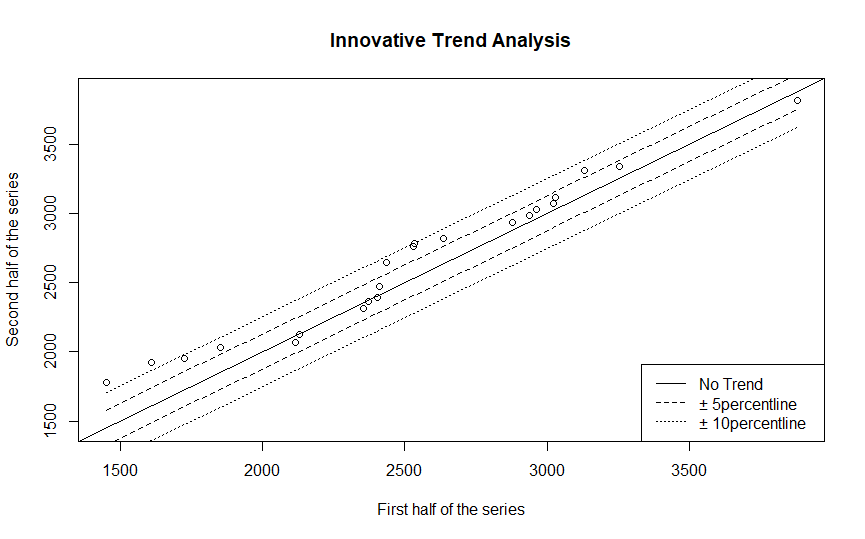 |
| (q) Mymensingh | (r) Patuakhali | (s) Rajshahi | (t) Rangamati |
| 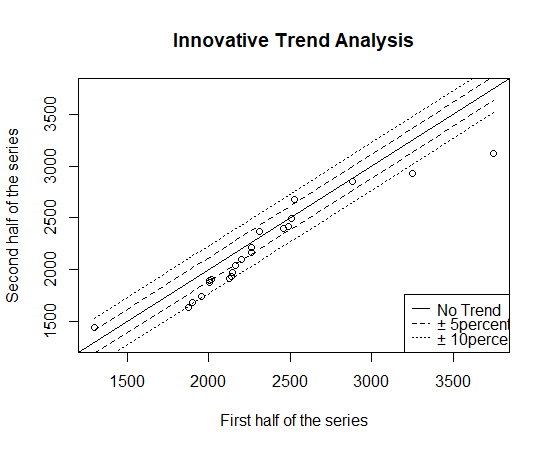 | 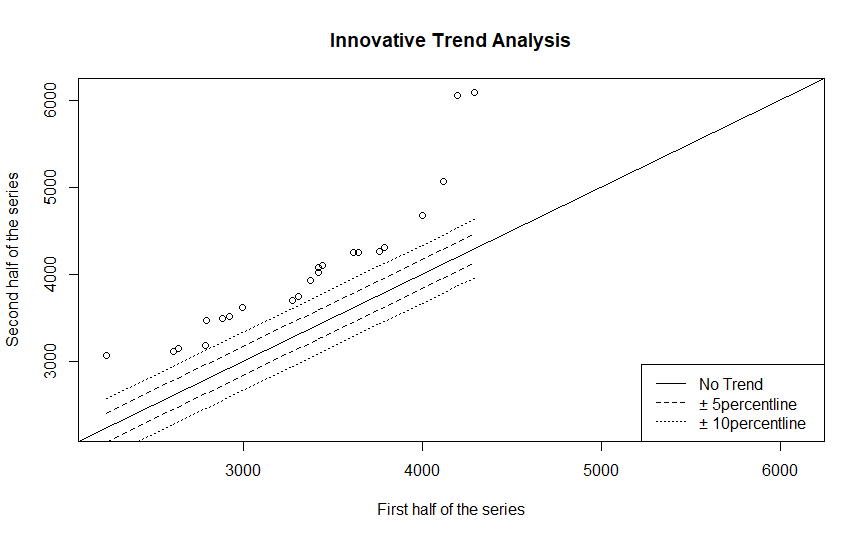 | 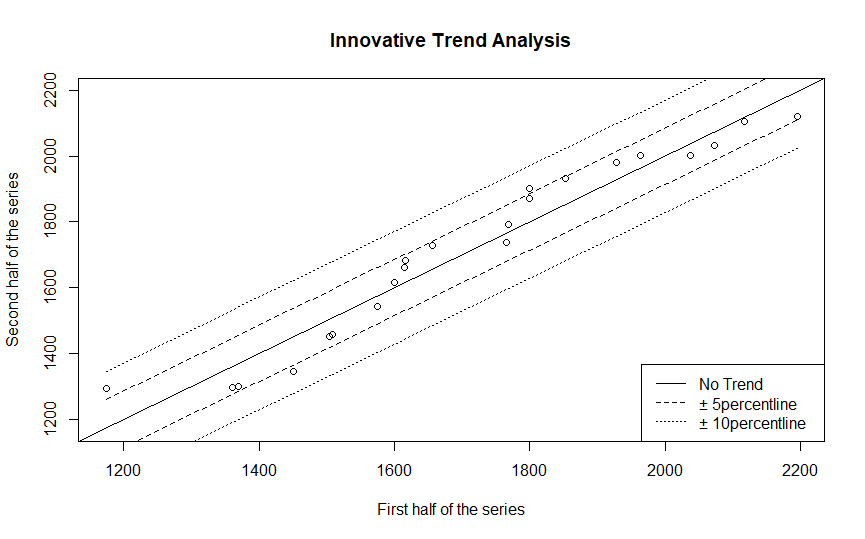 | 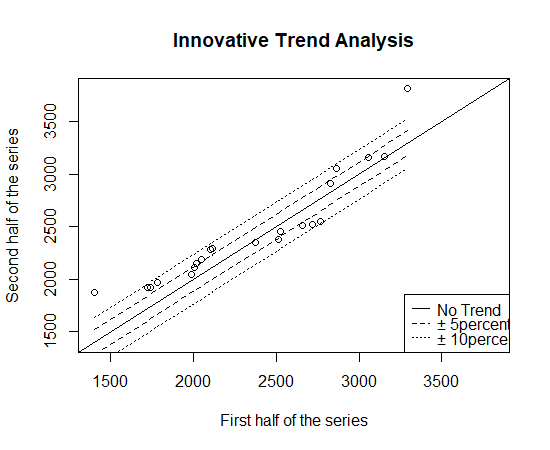 |
| (u) Rangpur | (v) Sandwip | (w) Satkhira | (x) Srimangal |
| 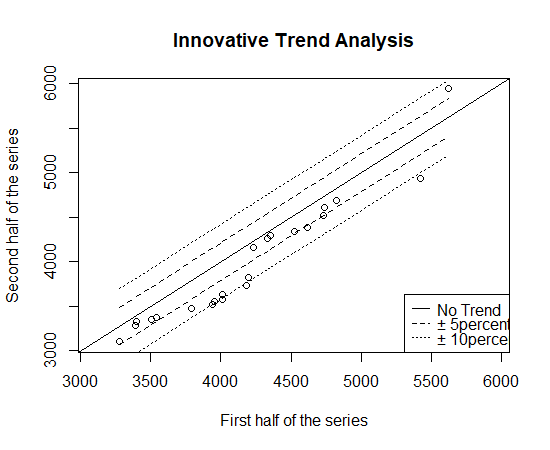 | | 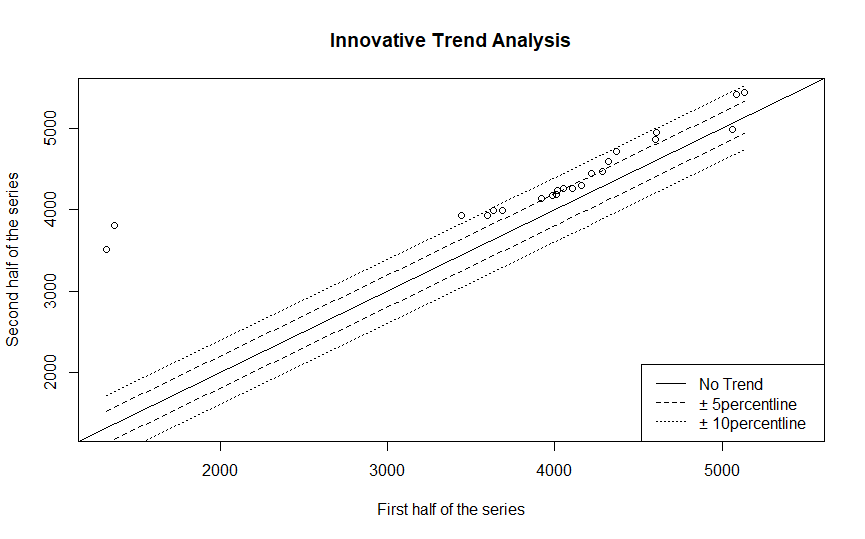 | |
| (y) Sylhet | | (z) Teknaf | |

Figure S6: ITA plots for annual rainfall series from 1975–2019 of (a) Barisal, (b) Bogra, (c) Chandpur, (d) Chittagong, (e) Comilla, (f) Cox's Bazar, (g) Dhaka, (h) Dinajpur, (i) Faridpur, (j) Feni, (k) Hatiya, (l) Ishurdi, (m) Jessore, (n) Khepupara, (o) Khulna, (p) M. Court, (q) Mymensingh, (r) Patuakhali, (s) Rajshahi, (t) Rangamati, (u) Rangpur, (v) Sandwip, (w) Satkhira, (x) Srimangal, (y) Sylhet and (z) Teknaf

| 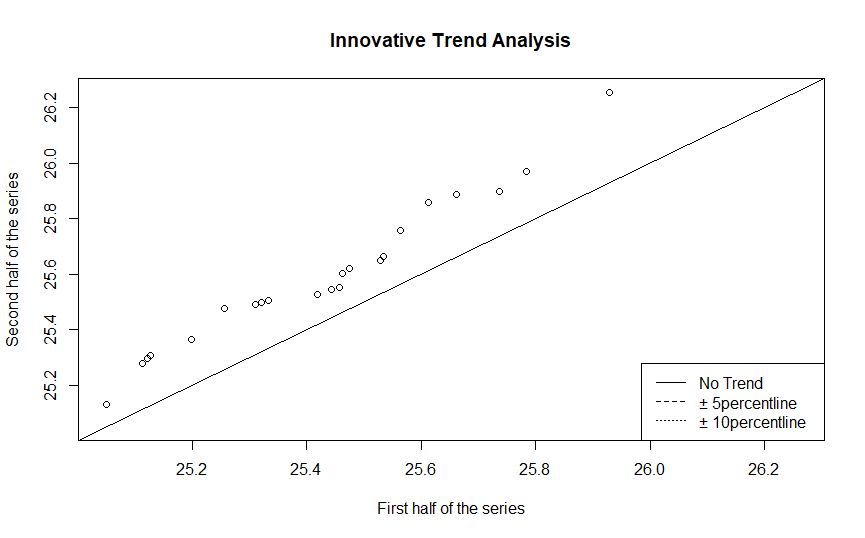 | 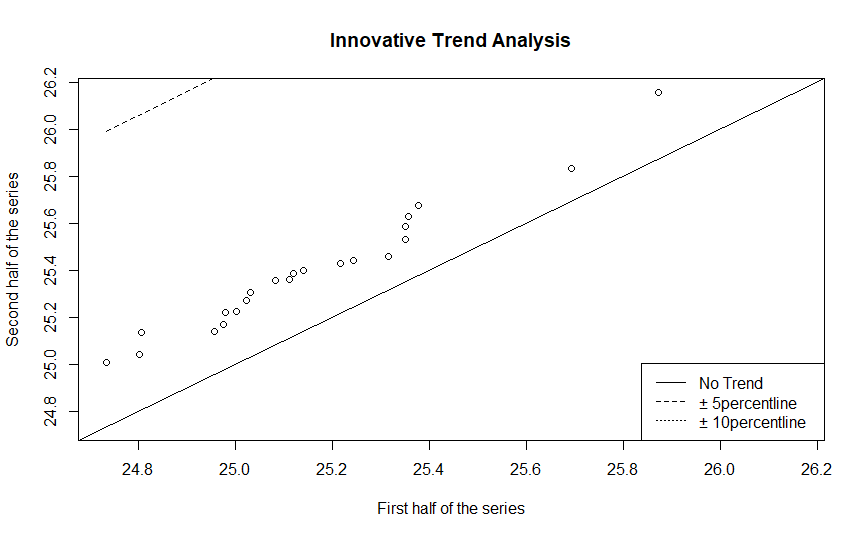 | 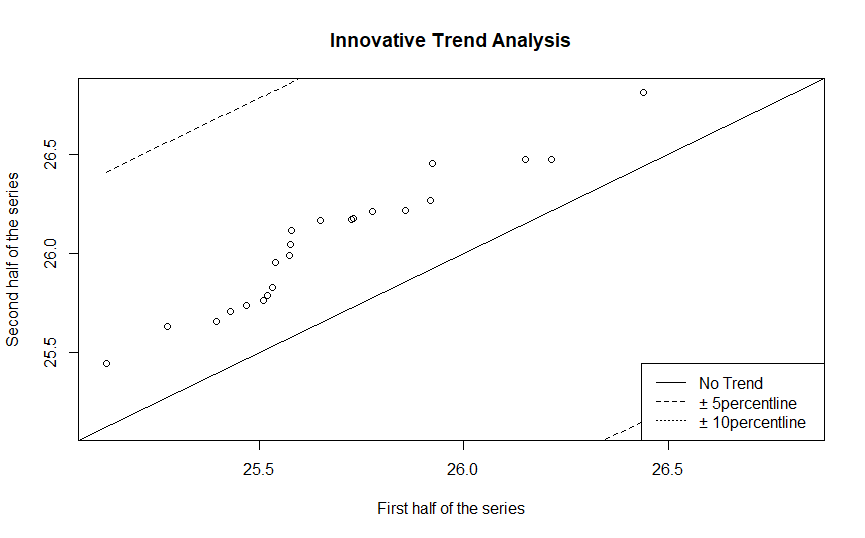 | 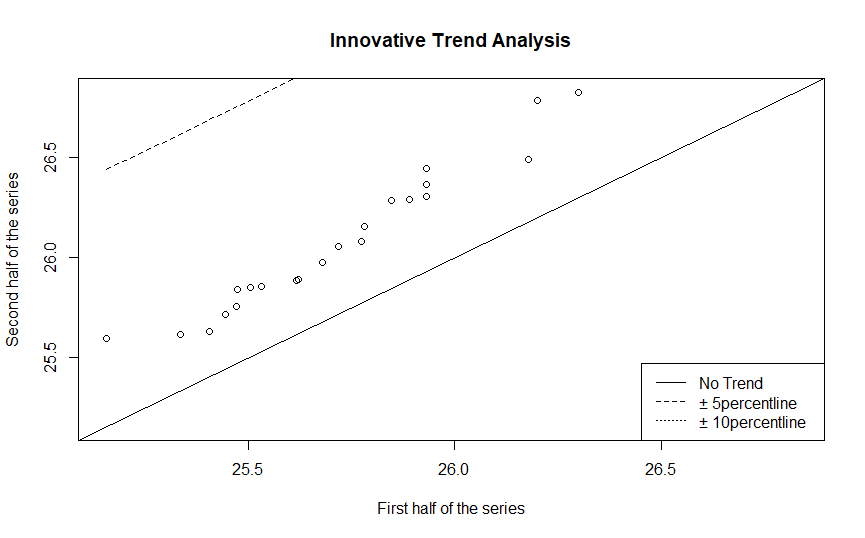 |
| --- | --- | --- | --- |
| (a) Barisal | (b) Bogra | (c) Chandpur | (d) Chittagong |
| 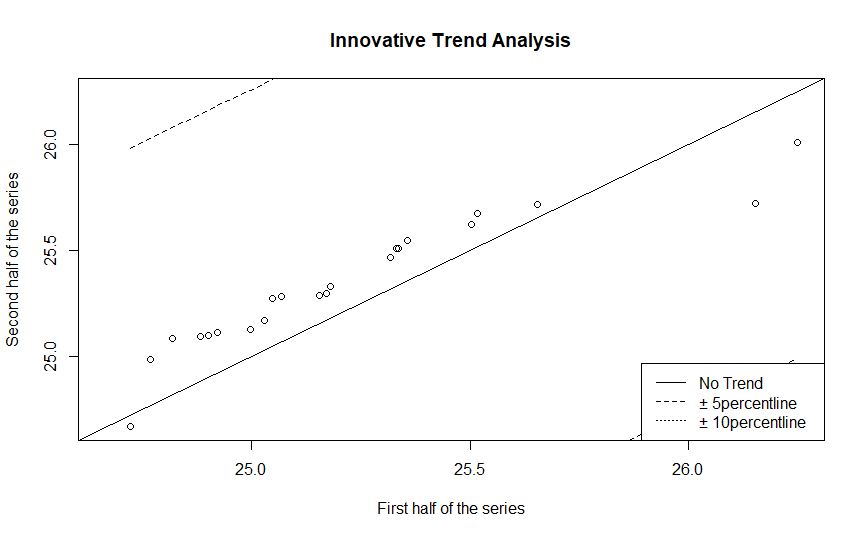 | 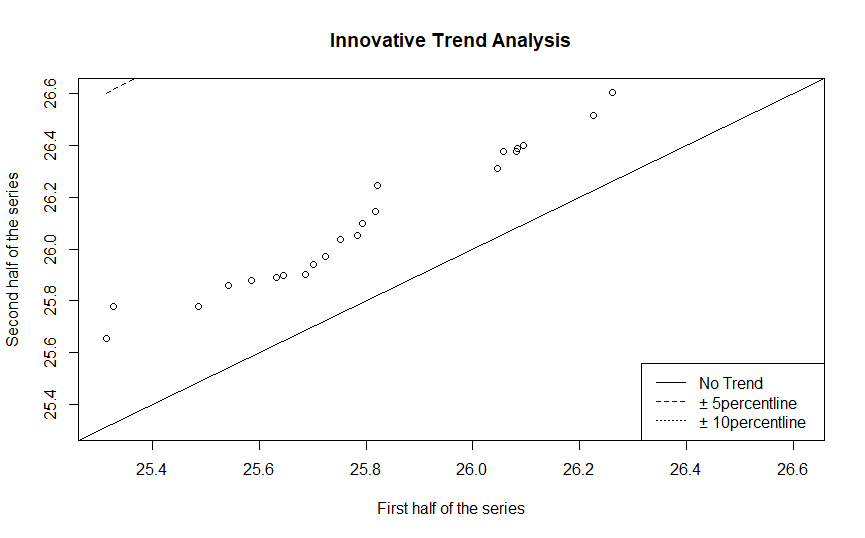 | 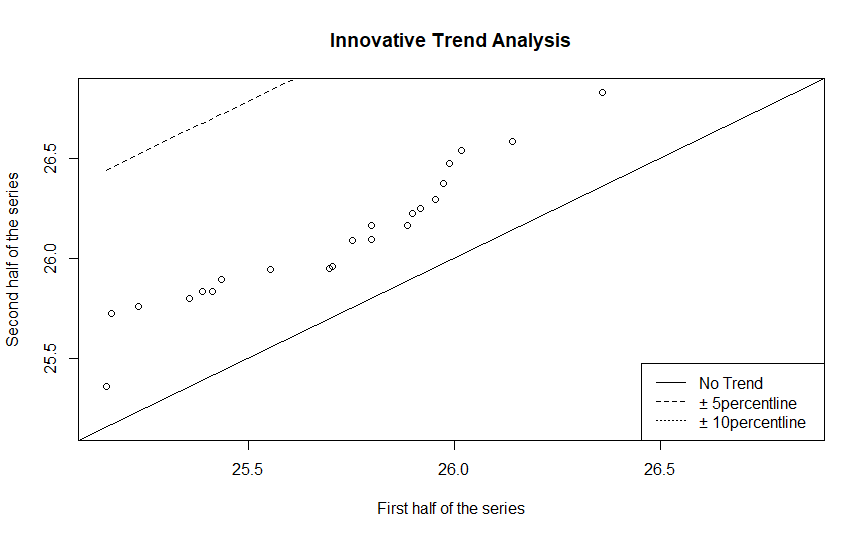 | 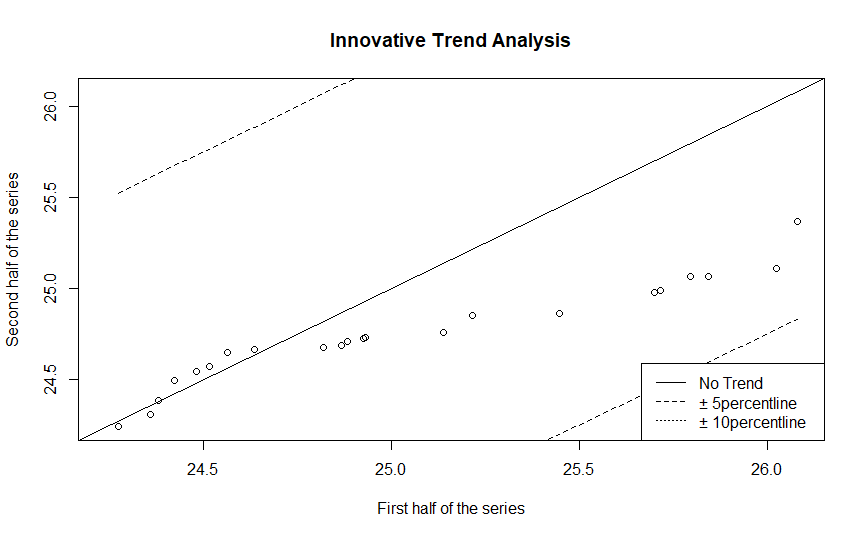 |
| (e) Comilla | (f) Cox’s Bazar | (g) Dhaka | (h) Dinajpur |
| 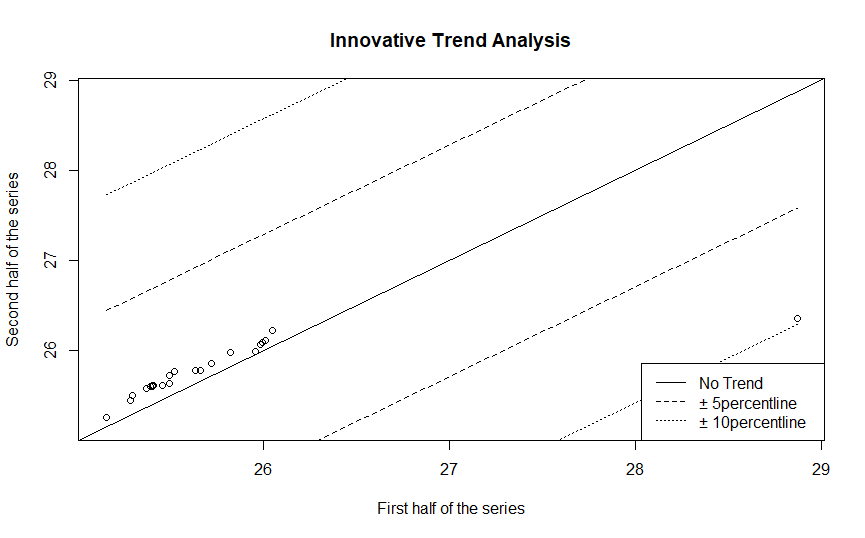 | 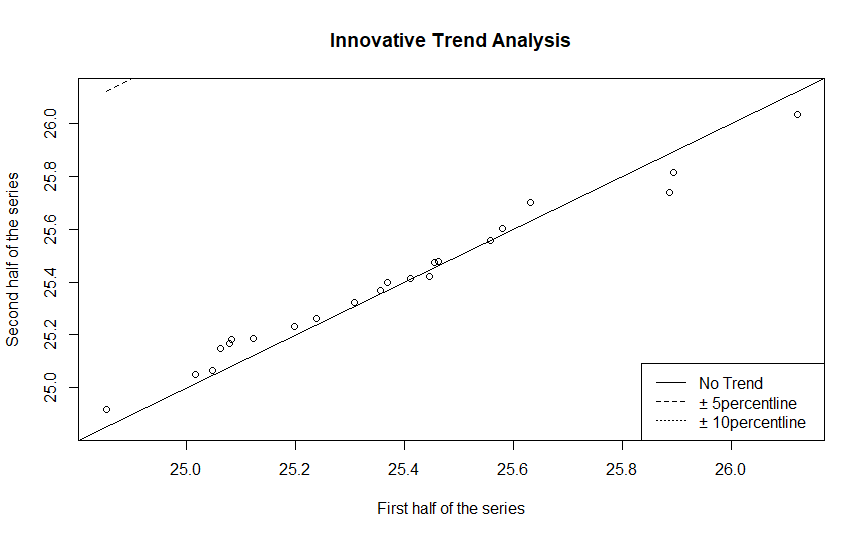 | 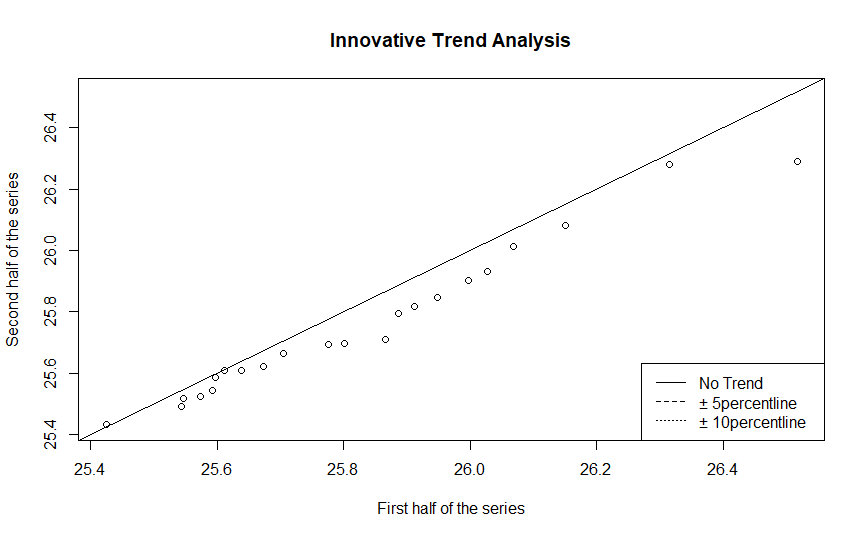 | 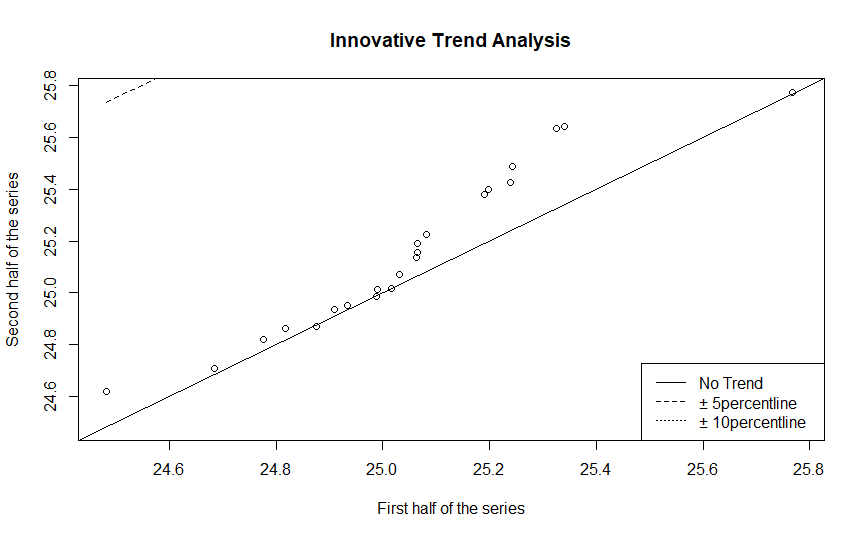 |
| (i) Faridpur | (j) Feni | (k) Hatiya | (l) Ishurdi |
| 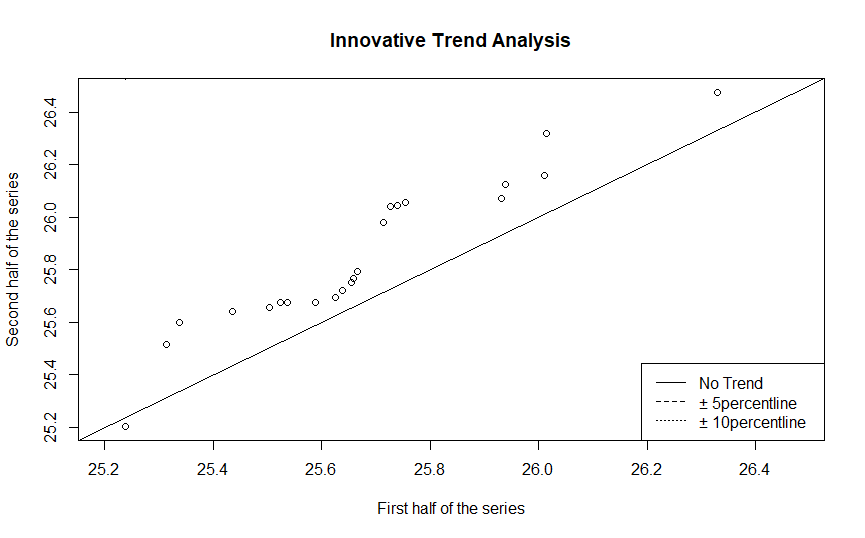 |  |  |  |
| (m) Jessore | (n) Khepupara | (o) Khulna | (p) M. Court |
|  |  |  |  |
| (q) Mymensingh | (r) Patuakhali | (s) Rajshahi | (t) Rangamati |
|  |  |  |  |
| (u) Rangpur | (v) Sandwip | (w) Satkhira | (x) Srimangal |
|  | |  | |
| (y) Sylhet | | (z) Teknaf | |

Figure S7: ITA plots for annual average temperature from 1975–2019 of (a) Barisal, (b) Bogra, (c) Chandpur, (d) Chittagong, (e) Comilla, (f) Cox's Bazar, (g) Dhaka, (h) Dinajpur, (i) Faridpur, (j) Feni, (k) Hatiya, (l) Ishurdi, (m) Jessore, (n) Khepupara, (o) Khulna, (p) M. Court, (q) Mymensingh, (r) Patuakhali, (s) Rajshahi, (t) Rangamati, (u) Rangpur, (v) Sandwip, (w) Satkhira, (x) Srimangal, (y) Sylhet and (z) Teknaf

|  |  |  |  |
| --- | --- | --- | --- |
| (a) Barisal | (b) Bogra | (c) Chandpur | (d) Chittagong |
|  |  |  |  |
| (e) Comilla | (f) Cox’s Bazar | (g) Dhaka | (h) Dinajpur |
|  |  |  |  |
| (i) Faridpur | (j) Feni | (k) Hatiya | (l) Ishurdi |
|  |  |  |  |
| (m) Jessore | (n) Khepupara | (o) Khulna | (p) M. Court |
|  |  |  |  |
| (q) Mymensingh | (r) Patuakhali | (s) Rajshahi | (t) Rangamati |
|  |  |  |  |
| (u) Rangpur | (v) Sandwip | (w) Satkhira | (x) Srimangal |
|  | |  | |
| (y) Sylhet | | (z) Teknaf | |

Figure S8: Standard anomaly index of average temperature series from 1975–2019 of (a) Barisal, (b) Bogra, (c) Chandpur, (d) Chittagong, (e) Comilla, (f) Cox's Bazar, (g) Dhaka, (h) Dinajpur, (i) Faridpur, (j) Feni, (k) Hatiya, (l) Ishurdi, (m) Jessore, (n) Khepupara, (o) Khulna, (p) M. Court, (q) Mymensingh, (r) Patuakhali, (s) Rajshahi, (t) Rangamati, (u) Rangpur, (v) Sandwip, (w) Satkhira, (x) Srimangal, (y) Sylhet and (z) Teknaf
